# Indenyl-thiazole and indenyl-formazan derivatives: Synthesis, anticancer screening studies, molecular-docking, and pharmacokinetic/ molin-spiration properties

**DOI:** 10.1371/journal.pone.0274459

**Published:** 2023-03-01

**Authors:** Ghaidaa H. Alfaifi, Thoraya A. Farghaly, Magda H. Abdellattif

**Affiliations:** 1 Chemistry Department, Faculty of Applied Sciences, Umm Al-Qura University, Makkah, Saudi Arabia; 2 Department of Chemistry, College of Science, Taif University, Taif, Saudi Arabia; Vignan Pharmacy College, INDIA

## Abstract

Two new series of thiazole and formazan linked to 5-Bromo-indan were synthesized, and their structures were assured based on all possible analytical techniques. The size of the tested derivatives was calculated from the XRD technique and found five derivatives **3**, **10a**, **14a, 15**, and **16** on the nanosized scale. The two series were tested for their efficacy and toxicity as anti-colon and stomach cancers. Derivative **10d** showed activity more than the two reference drugs used in the case of SNU-16. Surpislly, in the case of COLO205, five derivatives **4**, **6c**, **6d**, **6e**, and **10a** are better than the two benchmarks used, and two derivatives, **14a** and **14b** more potent than cisplatin. All potent derivatives showed a strong fit with the active site of the two tested proteins (gastric cancer (PDB = **2BID**) and colon cancer (PDB = **2A4L**)) in the molecular docking study. The Pharmacophore and ADME studies of the new derivatives showed that most derivatives revealed promising bioactivity, which indicates the drug-likeness properties against kinase inhibitors, protease, and enzyme inhibitors. In addition, the ProTox-II showed that the four compounds **10d**, **16**, **6d**, and **10a** are predicted to have oral LD_50_ values ranging from 335 to 3500 mg/kg in a rat model with (1 s,4 s)-Eucalyptol bearing the highest values and quercetin holding the lowest one.

## 1. Introduction

Different types of cancer are considered the most dangerous and deadly diseases globally [1, 2]. Numerous attempts have been made to find suitable ways to treat cancer. Chemotherapy is the primary approach in cancer treatment, where various natural and synthetic compounds are used to destroy cancer cells [3]. Despite the rapid advances in pharmacology and chemotherapeutic agents, the treatment of cancers remains a serious problem due to the toxicity, resistance, and lack selectivity of currently available anticancer drugs [4]. So far, the search is still going on for new compounds that are suitable and effective in treating different types of cancer [5–8]. On the other hand, nanoscale synthesis of heterocyclic materials is a scientific achievement due to its amazing applications in all medical and industrial fields [9, 10]. Heterocyclic-nanocomposites have effective biological activities due to their small size, allowing easy penetration of viral or microbial cell membranes [11, 12]. By browsing the heterocyclic compounds, we noted that thiazole derivatives spread in natural and industrial medicines, as they are a major part of cancer, histamine, microbes, and pressure medicines [13–15]. Several thiazoles with antiviral [16], antioxidant [17], antimicrobial [18], anticonvulsant [19], anti-inflammatory [20], and neuroprotective activities [21] are reported.

**Fig 1:**
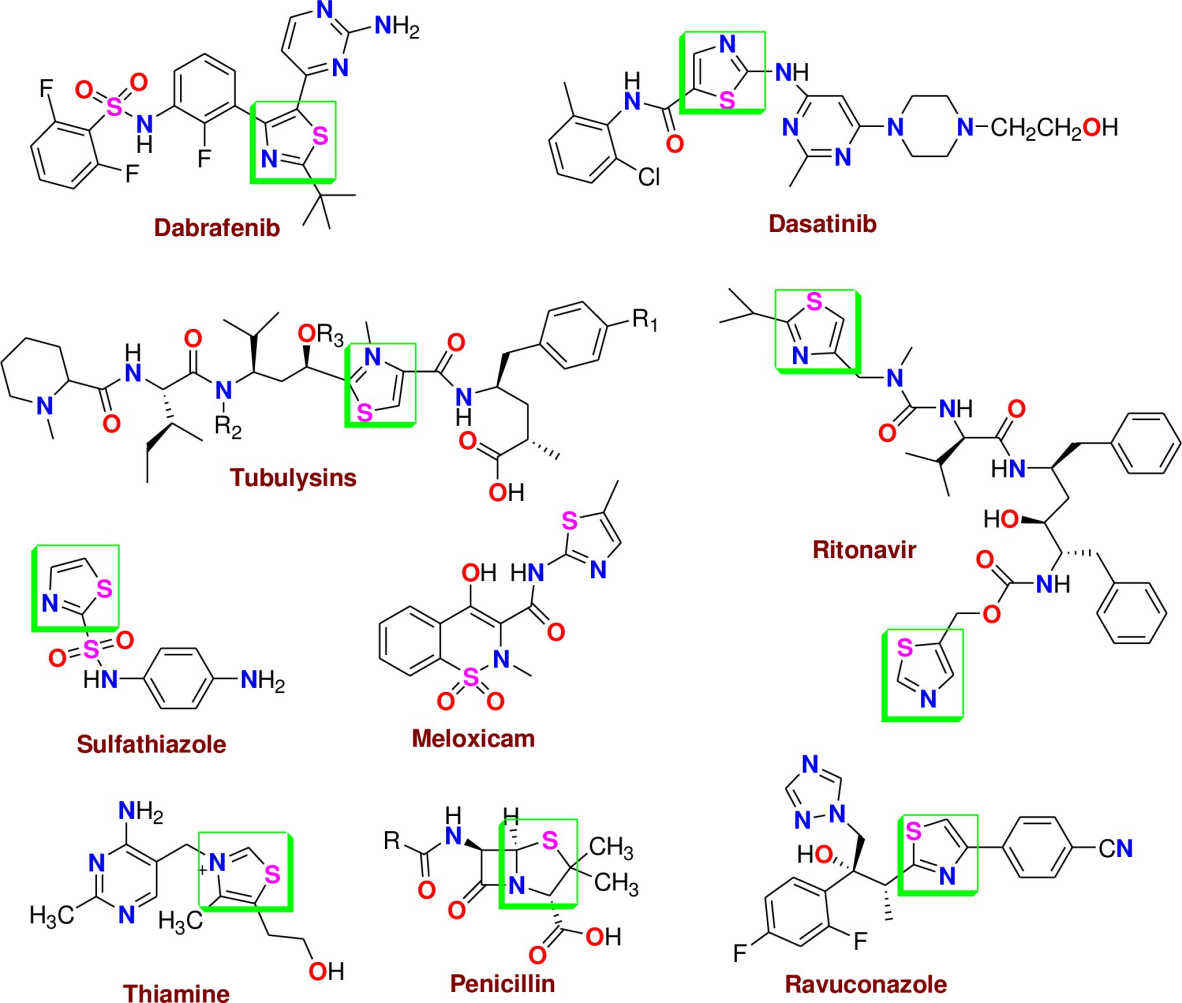
The natural compounds and marketing drugs containing thiazole moiety.

Fig 1 collected some drugs containing thiazole moiety. Another treasure of organic compounds is formazan derivatives with the general formula (-N = NC = NN -) [22] have been widely studied due to their numerous applications in the health and industrial field [23]. Multiple studies of the synthesis and applications of formazan derivatives showed that they have potent effects as antiparkinsonian [24], antiviral [25], antibacterial [26], antioxidant [27], anti-inflammatory [28], and anticancer activities [29]. Cyclic formazan derivatives are good indicators, especially for lithium-ion in blood [30]. Combining all the above findings with our research project concerning the synthesis of bioactive heterocyclic compounds [31–35], we focused on synthesizing a new series of thiazole and formazan derivatives and then investigating their anti-gastric and anti-colon cancer activities.

Furthermore, several previous studies of compounds containing thiazole moiety proved the effectiveness of these compounds as anticancer drugs [36–40]. The scenario of cancer is one of the most important in medicinal chemistry, while thiazoles are very important in treating cancers. It was reported the importance of the thiazole ring as a scaffold present in a wide range of therapeutic agents, the medicinal chemists have been encouraged to synthesize a large number of novel antitumors bearing this heterocycle, which furnish extensive synthetic possibilities due to the presence of several reaction sites [41–45].

By the way, the insilico-studies gave a chance to check the activities of these compounds, molecular docking studies with MOE and pharmacokinetics studies by molinspiration are good ways to discover and confirm the biological activities of the compounds under investigation [46–48].

## 2. Experimental

### 2.1. Chemistry

**Instruments** (See S1 File)

### 2.2. Synthesis of 2-(5-Bromo-2,3-dihydro-1H-inden-1-ylidene)hydrazine-1-carbothioamide (3)

In a 50 mL round Q.F. flask, we added 0.01 mole of 5-Bromo-indan-1-one **1** (2.11 g) with the same number of moles of thiosemicarbazide **2** (≈1g) in absolute ethanol (30 mL). The mixture was heated under reflux to dissolve the thiosemicarbazide; then, 1mL of concentrated HCl was added, and the reflux was completed to 2 h. The solid thiosemicarbazone 3 was filtered after the solution cold and crystallized from ethanol/dioxane to afford compound **3** as pale yellow, yield: 93%; M.p: 227–230°C; IR: 3430, 3366, 3147 (NH_2_, NH), 2917 (sp^3^-CH), 1593 (C = N), 1464, 1289, 1171 cm^-1^, ^1^H NMR (850 MHz, CDCl_3_): 2.84 (d, J = 8.5 *Hz*, 2H, CH_2_), 3.15–3.20 (m, 2H, CH_2_), 6.44 (s, 1H, NH), 7.33 (s, 1H, NH), 7.44 (d, J = 8.5 *Hz*, 1H, ArH), 7.54(s, 1H, ArH), 7.74 (d, J = 8.5 *Hz*, 1H, ArH), 8.69 (s, 1H, NH). ^13^C NMR (CDCl_3_): 26.7 (CH_2_), 28.3 (CH_2_), 122.9, 125.7, 128.9, 130.8, 135.8, 150.4, 156.5, 178.7 (C = S). MS(m/z): 285 (M^+^ +2, 29), 284 (M^+^ +1, 6), 283 (M^+^, 27), 268 (15), 266 (14), 129 (10), 115 (67), 102 (31), 90 (8), 89 (42), 76 (39), 60 (100). Elemental Analysis (%): (C_10_H_10_BrN_3_S; Mwt: 284.18) Calc. (Found): C, 42.27 (42.18); H, 3.55 (3.45); N, 14.79 (14.59)%.

### 2.3. Synthesis of (5-Bromo-indan-1-ylidene)-hydrazine (4)

In a 25 mL round Q.F. flask, we added 0.005 moles of 5-Bromo-indan-1-one **1** (1.06 g) in 20 mL EtOH and 1mL of NH_2_NH_2_.H_2_O were added, followed by refluxing the whale mixture for 5h. After the reaction (TLC mentoring), the pale-yellow solid was collected and washed with methanol to afford pale yellow crystals, yielding 78%; M.p: 120°C. IR: 3344, 3250 (NH_2_), 2918 (sp^3^-CH), 1469, 1351, 1286, 1169, 1107, 1055 cm^-1^. ^1^H NMR (850 MHz, CDCl_3_) ^1^H NMR(CDCl_3_): 2.8–2.7 (m, 2H, CH_2_), 3.0–3.11 (m, 2H, CH_2_), 5.20 (s, 2H, NH_2_), 7.37(d, J = 8.5 *Hz*, 1H, ArH), 7.45 (s, 1H, ArH), 7.5 (d, J = 8.5 *Hz*, 1H, ArH). ^13^C NMR (CDCl_3_): 24.9 (CH_2_), 28.1(CH_2_), 122.0, 123.1, 128.5, 130.3, 137.7, 148.6, 155.5. MS(m/z): 225 (M^+^, 2), 208 (13), 143 (7), 129 (37), 115 (100), 113 (10), 102 (40), 89 (20), 83 (14), 77 (15), 75 (17), 71 (20), 63 (24), 57 (32), 55 (27). Elemental Analysis (%): (C_9_H_9_BrN_2_; Mwt: 225.09) Calc. (Found): C, 48.02 (47.91); H, 4.03 (4.10); N, 12.45 (12.32)%.

**The reaction of 2-(5-Bromo-2,3-dihydro-1H-inden-1-ylidene)hydrazine-1-carbothioamide (3) or (5-Bromo-indan-1-ylidene)-hydrazine (4) with hydrazonoyl chlorides 5 or 13 or 13 and phenacyl bromide derivatives 9**

**Fig 2:**
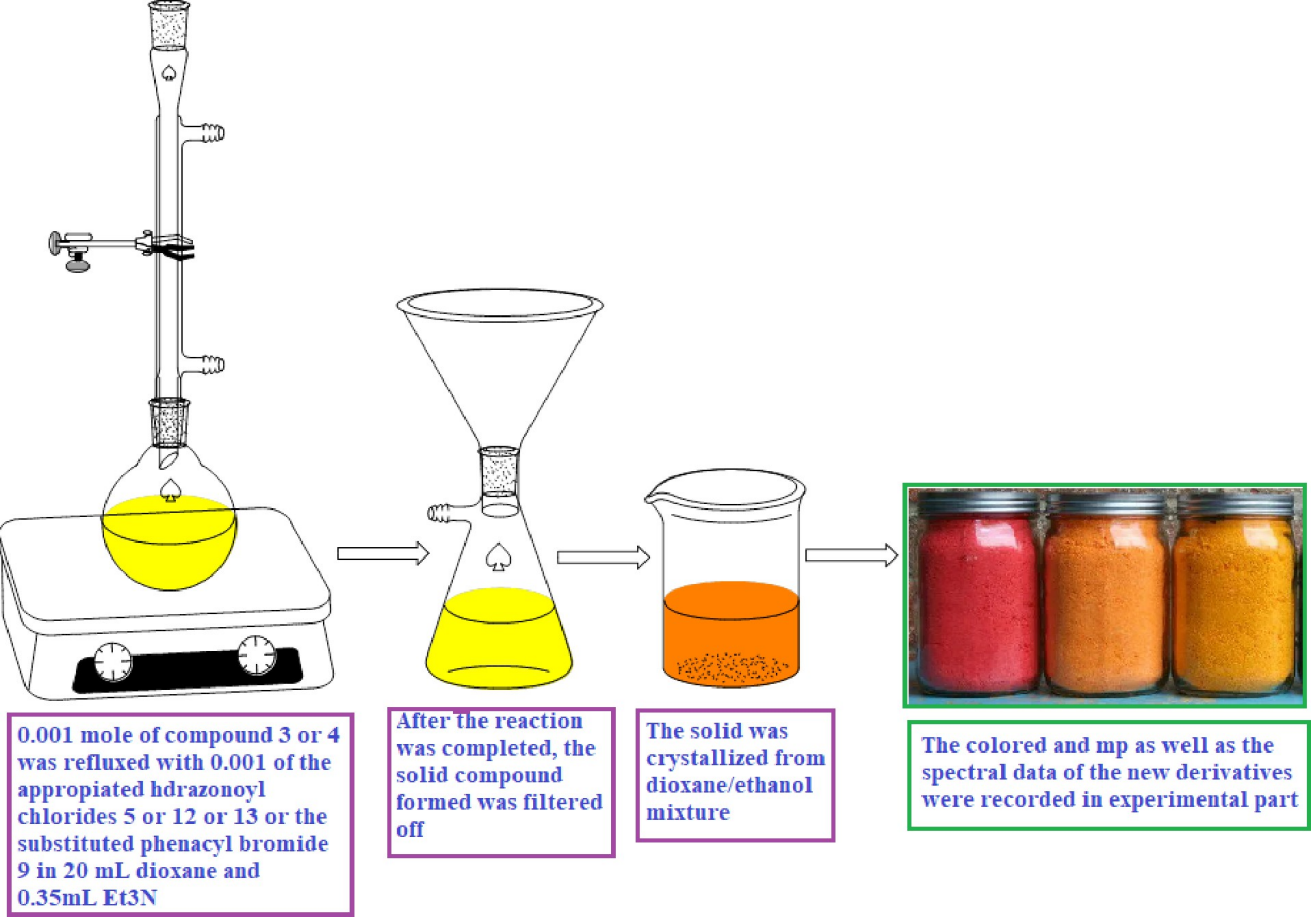
The method for synthesis of derivatives 6a-e, 10a-d, 14a, b, 15 and 16.

The method for synthesis of derivatives **6a-e**, **10a-d**, **14a, b**, **15,** and **16** as illustrated in Fig 2.

**2-(2h4(5-Bromo-2,3-dihydro-1H-inden-1-ylidene)hydrazineyl)-4-methyl-5-(phenyldiazenyl)-thiazole (6a)**

Orange solid, Yield: 88%; M.p: 220–222°C; IR: 3113 (NH), 3036 (sp^2^-CH), 2918 (sp^3^-CH), 1615 (C = N), 1546, 1410, 1378, 1252, 1169, 1117 cm^-1^. ^1^H NMR (850 MHz, CDCl_3_) 2.69 (s, 3H, CH_3_), 3.12–3.21 (m, 4H, 2CH_2_), 7.06 (t, J = 7.65Hz, 1H, ArH), 7.23 (d, J = 8.5 Hz, 2H, ArH), 7.37 (t, J = 6.8 Hz, 2H, ArH), 7.44 (s, 1H, ArH), 7.48 (d, J = 7.65 Hz, 1H, ArH), 7.57 (s, 1H, NH), 7.82 (d, J = 7.65 Hz, 1H, ArH). MS(m/z): 427 (M^+^ +1, 29), 426 (M^+^, 11), 425 (29), 285 (4), 129 (27), 115 (71), 112 (10), 102 (24), 92 (19), 77 (100), 65 (29). Elemental Analysis (%): (C_19_H_16_BrN_5_S; Mwt: 426.34) Calc. (Found): C, 53.53 (53.29); H, 3.78 (3.69); N, 16.43 (16.37)%.

**2-(2-(5-Bromo-2,3-dihydro-1H-inden-1-ylidene)hydrazineyl)-4-methyl-5-(m-tolyldiazenyl)-thiazole (6b)**

Green solid, Yield: 85%; M.p: 128–129°C; IR: 3400 (NH), 3048 (sp^2^-CH), 2918 (sp^3^-CH), 1595 (C = N), 1506, 1266, 1189, 1057 cm^-1^. ^1^H NMR(850 MHz, CDCl_3_): 2.36 (s, 3H, CH_3_), 2.39 (s, 3H, CH_3_), 2.66–3.16 (m, 4H, 2CH_2_), 6.87–7.57 (m, 7H, ArH), 7.62 (s, 1H, NH). ^13^C NMR (CDCl_3_): 18.4 (CH_3_), 21.5 (CH_3_), 28.4 (CH_2_), 29.6 (CH_2_), 111.4, 114.7, 124.1, 124.9, 129.0, 129.3, 130.0, 130.5, 130.9, 134.1, 139.5, 143.2, 156.7, 170.0, 174.1. 177.5. MS(m/z): 440 (M^+^, 3), 439 (5), 129 (19), 115 (53), 106 (18), 102 (20), 91 (100), 78 (10), 77 (27), 65 (24), 51 (7). Elemental Analysis (%): (C_20_H_18_BrN_5_S; Mwt 440.36) Calc. (Found): C, 54.55 (54.36); H, 4.12 (4.04); N, 15.90 (15.82)%.

**2-(2-(5-Bromo-2,3-dihydro-1H-inden-1-ylidene)hydrazineyl)-4-methyl-5-(p-tolyldiazenyl)thiazole (6c)**

Orange solid, Yield: 87%; M.p: 130–132°C; IR: 3213 (NH), 3181 (sp^2^-CH), 2917 (sp^3^-CH), 1612 (C = N), 1500, 1281, 1171, 1117 cm^-1^. ^1^H NMR(850 MHz, CDCl_3_): 2.35 (s, 3H, CH_3_), 2.39 (s,3H, CH_3_), 2.67–3.19 (m, 4H, 2CH2), 7.12–7.48 (m, 6H, ArH), 7.56 (s,1H, NH), 7.81 (d, J = 8.5Hz, 1H, ArH). ^13^C NMR (CDCl_3_): 16.7 (CH_3_), 22.7 (CH_3_), 28.5 (CH_2_), 29.7 (CH_2_), 114.1, 124.1, 126.5, 128.1, 129.0, 130.0, 132.6, 136.7, 140.2, 152.7, 174.1, 177.4, (two carbons overlapped). MS(m/z): 440 (M^+^, 3), 135 (5), 129 (22), 115 (58), 106 (18), 102 (20), 91 (100), 77(26), 65 (20), 51 (8). Elemental Analysis (%): (C_20_H_18_BrN_5_S; Mwt: 440.36) Calc. (Found): C, 54.55 (54.42); H, 4.12 (4.10); N, 15.90 (15.79)%.

**2-(2-(5-Bromo-2,3-dihydro-1H-inden-1-ylidene)hydrazineyl)-5-((3-chlorophenyl)-diazenyl)-4-methylthiazole (6d)**

Red solid, Yield: 89%; M.p: 180°C; IR: 3400 (br. NH), 1600 (C = N), 1508, 1468, 1374, 1242,1173, 1081. ^1^H NMR (DMSO-d_6_) 2.56 (s, 3H, CH_3_), 3.02–3.09 (m, 4H, 2CH_2_), 6.99–7.62 (m, 7H, ArH), 7.64 (s, 1H, NH). ^13^C NMR (DMSO-d_6_): 16.9 (CH_3_), 28.5 (CH_2_), 29.5 (CH_2_), 114.1, 123.9, 122.0, 123.9, 125.0, 126.1, 129.6, 130.5, 130.9, 131.0, 131.3, 134.3, 136.4, 136.8, 153.6, 158.0. MS(m/z): 461 (M^+^+2, 4), 460 (M^+^+1, 2), 459 (M+, 3), 183 (5), 139 (6), 129 (34), 115 (100), 111 (92), 102 (37), 99 (29), 95 (5), 89 (20), 77 (14), 75 (36), 67 (37), 63 (27), 57 (7), 55 (7), 51 (11). Elemental Analysis (%): (C_19_H_15_BrClN_5_S; Mwt: 460.78) Calc. (Found): C, 49.53 (49.48); H, 3.28 (3.19); N, 15.20 (15.08)%.

**2-(2-(5-Bromo-2,3-dihydro-1H-inden-1-ylidene)hydrazineyl)-5-((4-chlorophenyl)diazenyl)-4-methylthiazole (6e)**

Red solid, Yield: 83%; M.p: 177–178°C; IR: 3305 (NH), 3174 (sp^2^-CH), 2919 (sp^3^-CH), 1598 (C = N), 1487, 1299, 1169, 1024 cm^-1^. ^1^H NMR(850 MHz, CDCl_3_): 1.26–1.36 (m, 2H, CH_2_), 2.68(s, 3H, CH_3_), 3.13–3.20 (m, 2H, CH_2_), 7.16 (d, J = 8.5 Hz, 2H, ArH), 7.32 (d, J = 8.5Hz, 1H, ArH), 7.40 (s, 1H, ArH), 7.47 (d, J = 8.5 Hz, 2H, ArH), 7.57 (s, 1H, NH), 7.80 (d, J = 8.5Hz, 1H, ArH). ^13^C NMR (CDCl_3_): 16.7 (CH_3_), 27.7 (CH_2_), 28.5(CH_2_), 115.2, 124.1, 126.7, 127.7, 129.0, 129.5, 130.6, 136.6, 141.2, 141.6, 152.8, 169.5, 174.6, 177.5. MS(m/z): 460 (M^+^, 1), 459 (2), 210 (3), 141 (5), 129 (34), 115 (96), 111 (100), 102 (38), 99 (29), 89 (19), 77 (11), 75 (33), 71 (14), 67 (32), 63(22), 51(9). Elemental Analysis (%): (C_19_H_15_BrClN_5_S; Mwt: 460.78). Calc C, 49.53; H, 3.28; Br, 17.34; Cl, 7.69; N, 15.20; S, 6.96.

**N-(5-Bromo-indan-1-ylidene)-N’-(4-phenyl-thiazol-2-yl)-hydrazine (10a)**

Gray solid, Yield: 89%; M.p: 247–248°C; IR: 3400 (NH), 3052 (sp^2^-CH), 1616 (C = N), 1512, 1435, 1370, 1208, 1117, 1016 cm^-1^. ^1^H NMR (850 MHz, DMSO-d_6_): 2.88 (Br.s, 2H,CH_2_), 3.09 (Br.s, 2H,CH_2_), 7.32 (s, 1H, thiazole-H), 7.39–7.53 (m, 7H, ArH), 7.60 (s, 1H, NH), 7.88 (d, J = 8.5Hz, 1H, ArH). ^13^C NMR (DMSO-d_6_): 28.2 (CH_2_), 28.5 (CH_2_), 108.1, 122.8, 123.8, 126.0, 128.0, 128.6, 128.8, 129.2, 130.6, 133.4, 137.3, 150.9, 156.3, 165.8. MS(m/z): 384 (M^+^, 12), 383 (24), 210 (4), 176 (45), 148 (9), 134 (100), 121 (12), 115 (24), 102 (30), 89 (17), 77 (20), 63 (8), 51 (9). Elemental Analysis (%): (C_18_H_14_BrN_3_S; Mwt: 384.30). Calc. (Found) C, 56.26 (56.09); H, 3.67 (3.56); N, 10.93 (10.84)%.

**N-(5-Bromo-indan-1-ylidene)-N’-(4-p-tolyl-thiazol-2-yl)-hydrazine (10b)**

Gray solid, Yield: 90%; M.p: 233–234°C; IR: 3200 (NH), 3065 (sp^2^-CH), 2917 (sp^3^-CH), 1613 (C = N), 1508, 1468, 1205, 1172, 1060, 1016 cm^-1^. ^1^H NMR (850 MHz, DMSO-d_6_): 2.32 (s, 3H, CH_3_), 2.89–3.11 (m, 4H, 2CH₂), 7. 22–7.77 (m, 9H, ArH, thiazole-H and NH). ^13^C NMR (DMSO-d_6_): 21.2 (CH_3_), 28.0 (CH₂), 28.5 (CH₂), 103.4, 122.7, 123.5, 125.9, 128.0, 1 29.1, 129.4 129.6, 130.6, 130.7, 137.3, 137.6, 150.7, 169.6. Mass (m/z): 400 (M^+^ +2, 22), 398 (M^+^, 44), 190(51), 162(10), 148(100), 129(29), 115(52), 102(39), 89(20), 75(10). MS(m/z): 399(M^+^ +2, 88), 397 (M^+^+1, 87), 339 (M+, 4), 289(5), 190(51), 174(9), 162(10), 148(100), 135(10), 129(29), 115(53), 102(38), 91(25), 77(18), 63(15), 51(11). Elemental Analysis (%): (C_19_H_16_BrN_3_S; Mwt: 398.32). Calc. (Found): C, 57.29 (57.16); H, 4.05 (3.93); N, 10.55 (10.43)%.

**N-(5-Bromo-indan-1-ylidene)-N’-[4-(4-bromo-phenyl)-thiazol-2-yl]-hydrazine (10c)**

Gray solid, Yield: 91%; M.p: 230–232°C; IR: 3200 (NH), 3048 (sp^2^-CH), 2919 (sp^3^-CH), 1610 (C = N), 1491, 1368, 1205, 1104, 1001, 824 cm^-1^. ^1^H NMR (850 MHz, DMSO-d_6_): 2.88 (t, J = 8.5Hz, 2H₁ CH₂), 3.10 (t, J = 8.5 Hz, 2H, CH₂), 7.41 (s, 1H, thiazole-H) 7.5–7.83 (m, 8H, Ar-H and NH).^13^C NMR (DMSO-d_6_): 28.0 (CH₂), 28.4 (CH₂), 105.2, 120.9, 122.7, 123.5, 128.0, 129.1, 1 30.6, 132.0 134.4, 137.6, 150.7, 155.3, 169.9. MS(m/z): 463 (M^+^, 25), 461 (15), 256 (50), 210 (17), 182 (5), 174 (100), 155 (5), 146 (25), 133 (17), 129 (74), 120 (25), 115 (79), 102 (87), 95 (5), 89 (44), 75 (32), 63 (22), 57 (10), 51 (20). Elemental Analysis (%): (C_18_H_13_Br_2_N_3_S; Mwt: 463.19). Calc. (Found): C, 46.68 (46.52); H, 2.83 (2.78); N, 9.07 (9.01)%.

**N-(5-Bromo-indan-1-ylidene)-N’-[4-(4-chloro-phenyl)-thiazol-2-yl]-hydrazine (10d)**

Gray solid, Yield: 88%; M.p: 255–256°C; IR: 3250 (NH), 3050 (sp^2^ CH), 2922 (sp^3^ CH), 1616 (C = N), 1491, 1371, 1272, 1208, 1172, 1094, 1010 cm^-1^. ^1^H NMR (850 MHz, DMSO-d_6_): 2.88 (t, J = 8.5Hz, 2H, CH₂), 3.08 (t, J = 8.5 Hz, 2H, 2H₂), 7.39 (s, 1H, thiazole-H), 7.46 (t, J = 8.5 Hz, 2H, ArH), 7.52–7.55 (m, 3H, ArH), 7.60 (s, 1H, NH), 7.88–7.91 (m, 2H, ArH). ^13^C NMR (DMSO-d_6_): 28.0 (CH₂), 28.4 (CH₂), 105.1, 122.8, 123.5, 127.7, 129.0, 129.1 130.6, 132.3, 137.5, 150.7, 155.4, 156.5, 165.9, 169.9. MS(m/z): 419 (M^+^ +2,100), 418 (M^+^+1,43), 417(M+, 76), 210 (67), 174 (21), 168 (19), 146 (5), 136(5), 129 (30), 115 (42), 111 (12), 102 (59), 89 (25), 75 (21), 63 (10), 51 (5). Elemental Analysis (%): (C_18_H_13_BrClN_3_S; Mwt: 418.74). Calc. (Found) C, 51.63 (51.54); H, 3.13 (3.08); N, 10.03 (9.98)%.

**N’-(5-bromo-2,3-dihydro-1H-inden-1-ylidene)-2-oxo-N’’-phenylpropanehydrazonhydrazide (Formazan, 14a)**

Yellow solid, Yield: 89%; M.p: 190°C; IR: 3294, 3205 (2NH), 3025 (sp^2^ CH), 1667 (C = O), 1600 (C = N), 1485, 1247, 1148 cm^-1^. ^1^H NMR(850 MHz, CDCl_3_): 2.56 (s, 3H, CH_3_), 2.78 (t, J = 8.5 Hz, 2H, CH_2_), 3.16 (t, J = 8.5Hz, 2H, CH₂), 6.93 (t, J = 8.5Hz,1H, ArH), 7.17 (d, J = 8.5 Hz, 2H, ArH), 7.33 (t, J = 8.5Hz, 2H, ArH), 7.45–7.50 (m, 3H, ArH), 8.47 (s, 1H, NH), 11.62 (s, 1H, NH). MS(m/z): 384 (M^+^, 3), 212 (5), 210 (8), 176 (14), 115 (26), 105 (34), 102 (17), 92 (25), 77 (100), 70 (12), 65 (30). Elemental Analysis (%): (C_18_H_17_BrN_4_O; Mwt: 385.27). Calc. (Found) C, 56.12 (56.03); H, 4.45 (4.31); N, 14.54 (14.42)%.

**N’-(5-bromo-2,3-dihydro-1H-inden-1-ylidene)-N’’-(4-chlorophenyl)-2-oxopropanehydrazon-hydrazide (Formazan, 14b)**

Yellow solid, Yield: 87%; M.p: 197–198°C; IR: 3331, 3318 (2NH), 1670(C = O), 1596(C = N), 1361, 1243, 1159 cm^-1^. ^1^H NMR(850 MHz, CDCl_3_): 2.56 (s, 3H, CH_3_), 2.83 (t, J = 8.5Hz, 2H, CH₂), 3.19 (t, J = 8.5Hz, 2H, CH₂), 7.10 (d, J = 8.5Hz, 2H, ArH), 7.3 (s, 1H, ArH), 7.45–7.53 (m, 4H, ArH), 8.50 (s, 1H, NH), 11.67 (s, 1H, NH). ^13^C NMR (CDCl_3_): 23.5(CH_3_), 25.1(CH₂), 28.2(CH₂), 114.1, 121.5, 124.2, 125.1, 128.9, 129.3, 130.7, 132.2, 136.4, 142.6, 149.2, 152.6, 192.9 (C = O). MS(m/z): 420 (M^+^+1, 3), 419 (M^+^, 4), 210 (76), 166 (8), 141 (63), 129 (45), 115 (59), 111 (100), 99 (45), 89 (16), 75 (40), 70 (27), 63 (23), 51 (10). Elemental Analysis (%): (C_18_H_16_BrClN_4_O; Mwt: 419.71). Calc. (Found) C, 51.51 (51.42); H, 3.84 (3.75); N, 13.35 (13.29)%.

**Ethyl 2-(2-(5-bromo-2,3-dihydro-1H-inden-1-ylidene)hydrazineyl)-2-(2-phenyl-hydrazineylidene)acetate (Formazan, 15)**

Red solid, Yield: 87%; M.p: 174–176°C IR: 3329, 3252 (2NH), 3050 (sp^2^ CH), 2984 (sp^3^ CH), 1693 (C = O), 1602 (C = N), 1489, 1370, 1247, 1165 cm^-1^. ^1^H NMR(850 MHz, CDCl_3_): 1.44 (t, J = 8.5Hz, 3H, CH_3_), 2.78 (t, J = 8.5Hz, 2H, CH₂), 3.17 (t, J = 8.5Hz, 2H, CH_2_), 4.36 (q, J = 8.5 Hz, 2H, CH₂), 6.89 (t, J = 8.5Hz, 1H, ArH), 7.15 (d, J = 8.5Hz, 2H, ArH), 7.30 (t, J = 8.5Hz, 2H, ArH), 7.48 (d, J = 8.5Hz, 2H, ArH), 7.50 (s, 1H, ArH), 8.37 (s, 1H, NH), 11.46 (s, 1H, NH). ^13^C NMR (CDCl_3_): 14.2 (CH_3_), 25.0 (CH₂), 28.2 (CH₂), 62.3 (CH₂), 112.9, 120.1, 121.6, 124.1, 124.6, 128.9, 129.2, 130.7, 136.4, 144.2, 149.1, 152.1, 162.6 (C = O). MS(m/z): 416 (M^+^ +2, 5), 415 (M^+^+1, 5), 414 (M^+^, 7), 206 (5), 129 (11), 115 (21), 105 (30), 92 (17), 89 (5), 77 (100), 65 (20), 51 (9). Elemental Analysis (%): (C_19_H_19_BrN_4_O_2_; Mwt: 415.29). Calc. (Found): C, 54.95 (54.81); H, 4.61 (4.53); N, 13.49 (13.29)%.

**2-(2-(5-Bromo-2,3-dihydro-1H-inden-1-ylidene)hydrazineyl)-N-phenyl-2-(2-phenyl-hydrazineylidene)acetamide (Formazan, 16)**

Yellow solid, Yield: 82%; M.p: 230–232°C IR: 3356, 3326, 3247 (3NH), 3052 (sp^2^ CH), 1662 (C = O), 1602 (C = N), 1539, 1488, 1444, 1226, 1168 cm^-1^. ^1^H NMR(850 MHz, CDCl_3_): 2.83 (m, 2H, CH₂), 3.19 (t, J = 8.5Hz, 2H, CH₂), 6.92–7.66 (m, 13H, ArH), 8.81 (s, 1H, NH), 9.13(s, 1H, NH), 11.30 (s, 1H, NH). ^13^C NMR (CDCl_3_): 28.3 (CH₂), 29.7 (CH₂), 112.5 119.5, 119.9, 121.6, 124.2, 124.4, 128.9, 129.1, 129.4, 130.7, 136.4, 137.2, 144.2, 149.3, 153.1, 159.1 (C = O). MS(m/z): 463 (M^+^ +2, 1), 462 (M^+^+1, 1), 461 (M^+^,1), 147 (7), 129 (9), 120 (24), 115 (14), 107 (23), 102 (10), 92 (31), 77 (100), 65 (23), 51 (12). Elemental Analysis (%): (C_23_H_20_BrN_5_O; Mwt: 462.35). Calc. (Found): C, 59.75 (59.63); H, 4.36 (4.29); N, 15.15 (15.05)%.

### 2.4. Biological studies

#### Anticancer screening

*A- Cell culture*. The cells were obtained from the Egyptian Holding Company for Biological Products & Vaccines (VACSERA), Giza, Egypt, then maintained in the tissue culture unit. The cells were grown in RBMI‐1640 medium, supplemented with 10% heat-inactivated FBS, 50 units/mL of penicillin, and 50 mg/mL of streptomycin, and maintained at 23 in a humidified atmosphere containing 5% CO_2_. The cells were maintained as monolayer culture by serial sub‐culturing. Cell culture reagents were obtained from Lonza (Basel, Switzerland). The anticancer activity of the rested compounds was evaluated against Gastric (SNU-16) Patch number (ATCC CRL-5822) (Gastric cancer) and COLO205 cells (colon cancer) [47, 49].

#### The sulforhodamine B (SRB) cytotoxicity assay

Sulforhodamine B(SRB) assay method was used to evaluate cytotoxicity [50, 51]. Exponentially growing cells were collected using 0.25% Trypsin‐EDTA seeded in 96‐well plates at 1000‐2000 cells/well in RBMI‐1640 supplemented medium. After 24 h, cells were incubated for 72 h with various concentrations of the tested compounds. Following 72 h treatments, the cells would be fixed with 10% trichloroacetic acid for 1 h at 4°C. Wells were stained for 10 minutes at room temperature with 0.4% SRBC (Sulphorhodamine B) dissolved in 1% acetic acid. After that, the plates were air-dried for 24 h. At the same time, the dye was dissolved in Tris‐HCl and left for 5 min on the shaker at 1600 rpm. Each well’s optical density (OD) was measured spectrophotometrically at 564 nm; by using an ELISA microplate reader (ChroMate‐4300, FL, USA). The IC_50_ values were calculated according to the equation for Boltzman sigmoidal concentration-response curve using the nonlinear regression fitting models (Graph Pad, Prism Version 9).

### 2.5. In-Silico ADME study

#### A-Molecular docking

The docking analyses were characterized by the Molecular Environment (MOE) software. Chemdraw compound preparation, Chem 3d structures, Chem 3D 16 (Molecular Modeling and Analysis; Cambridge Soft Corporation) software, to dock the complexes toward the Colon cancer (PDB = 2A4L), Gastric cancer (PDB = PDB = 2BID). The crystal structure was downloaded from the PDB at www.rcsb.org. The following procedure was applied the water molecules, co-ligand, and metal ions were removed, and the final form was obtained after 3D protonation and the correction process. The MOE site finder generated the active binding sites to create the dummy sites as the binding pocket. The default docking parameters were triangle matcher for replacing the molecule and London dG for rescoring the docking scores. The higher negative values of the docking scores were presented along with 2D and 3D structures [52–54].

#### B-Pharmacophore and ADME studies

A computational study of indane derivatives based on the thiazole nucleus was performed to predict ADME properties by the QikProp3.2 tool available in Schrödinger 9.0 version (USA) and Molinspiration online property calculation toolkit to get an idea of whether the compound has optimum pharmacokinetic properties to enter higher phases of the drug development process or not. Molinspiration strategy may be described as a complex balance of various molecular properties and structural features which conclude whether the appropriate molecule is related to the known drugs [55, 56].

### 2.6. Assessment of the safety profile

The ProTox-II software predicts different toxicity endpoints, such as acute toxicity, hepatotoxicity, carcinogenicity, and mutagenicity [50, 56]. The Pred-hERG (human Ether-a-go-go-Related Gene) software was used to assess cardiotoxicity. It depends on statistically significant and externally predictive quantitative structure-activity relationship (QSAR) models of hERG blockage closely associated with severe and potentially fatal cardiac dysrhythmia. The SDF (structure data file) and SMILES (simplified molecular-input line-entry system) strings were used throughout the generation process [45].

## 3. Results and discussion

### 3.1 Chemistry

**Scheme 1:**
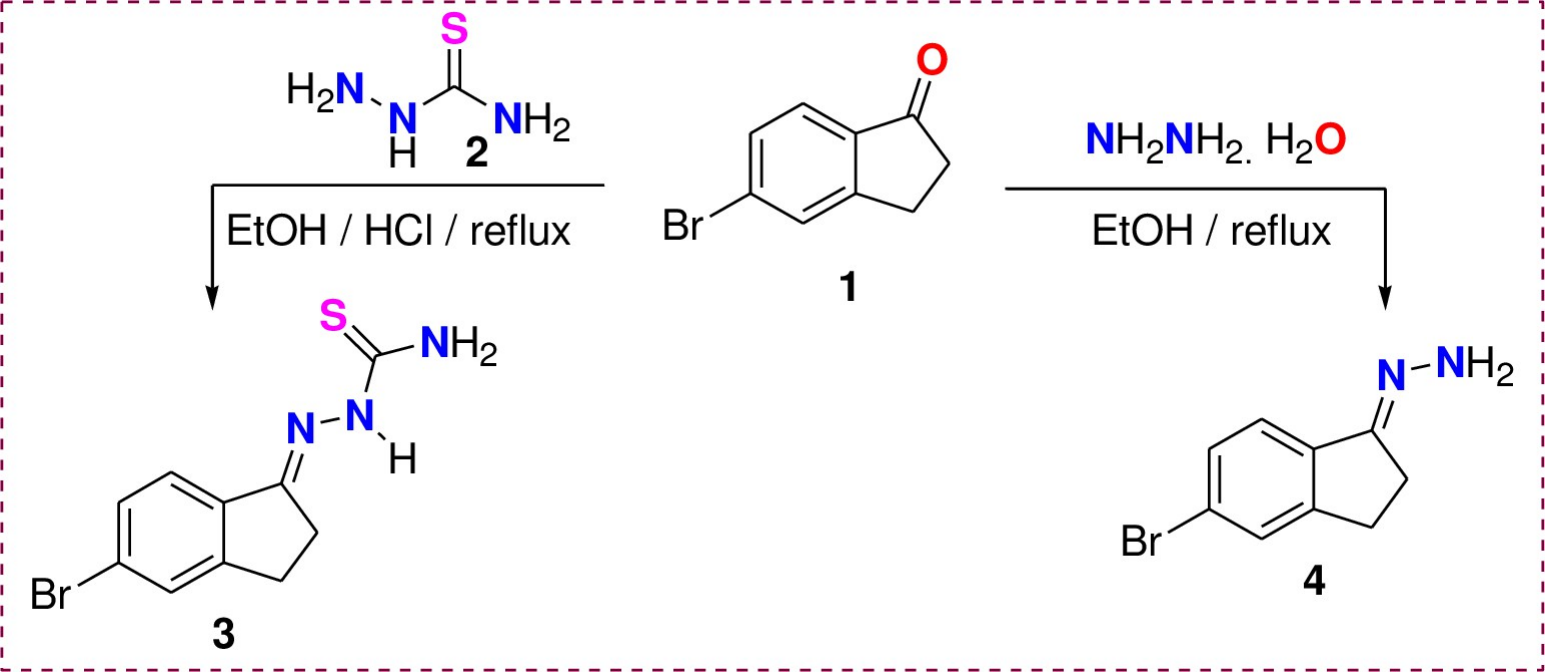
Synthesis of compounds 3 and 4.

This research article synthesized the targeted two starting compounds, thiosemicarbazone, and hydrazone of 5-Bromo-indan-1-one 3 and 4, as indicated in Scheme 1 from the condensation reaction **1** with each of thiosemicarbazone **2** and hydrazine hydrate in refluxing acidified ethanol or pure ethanol, respectively. The structure of derivatives **3** and **4** were confirmed based on their ^1^H NMR, IR, Mass, and EA (elemental analysis) data. For instance, the ^1^H NMR spectrum of derivatives **3** revealed all expected signals as follows: δ = 2.84, 3.15 (two CH_2_), 6.44, 7.33, 8.69 (three NH), and 7.44–7.74 (three H_Ar_) ppm. The appearance of three NH singlet signals in ^1^H NMR of **3** is attributed to the two protons of the amino group being non-equivalent as one NH contributed to the H-bond with the C = N group. In addition, ^13^C NMR of the same compound 3 showed ten carbon signals at δ = 26.7 (CH_2_), 28.3 (CH_2_), 122.9, 125.7, 128.9, 130.8, 135.8, 150.4, 156.5 and 178.7 (C = S) ppm.

**Fig 3:**
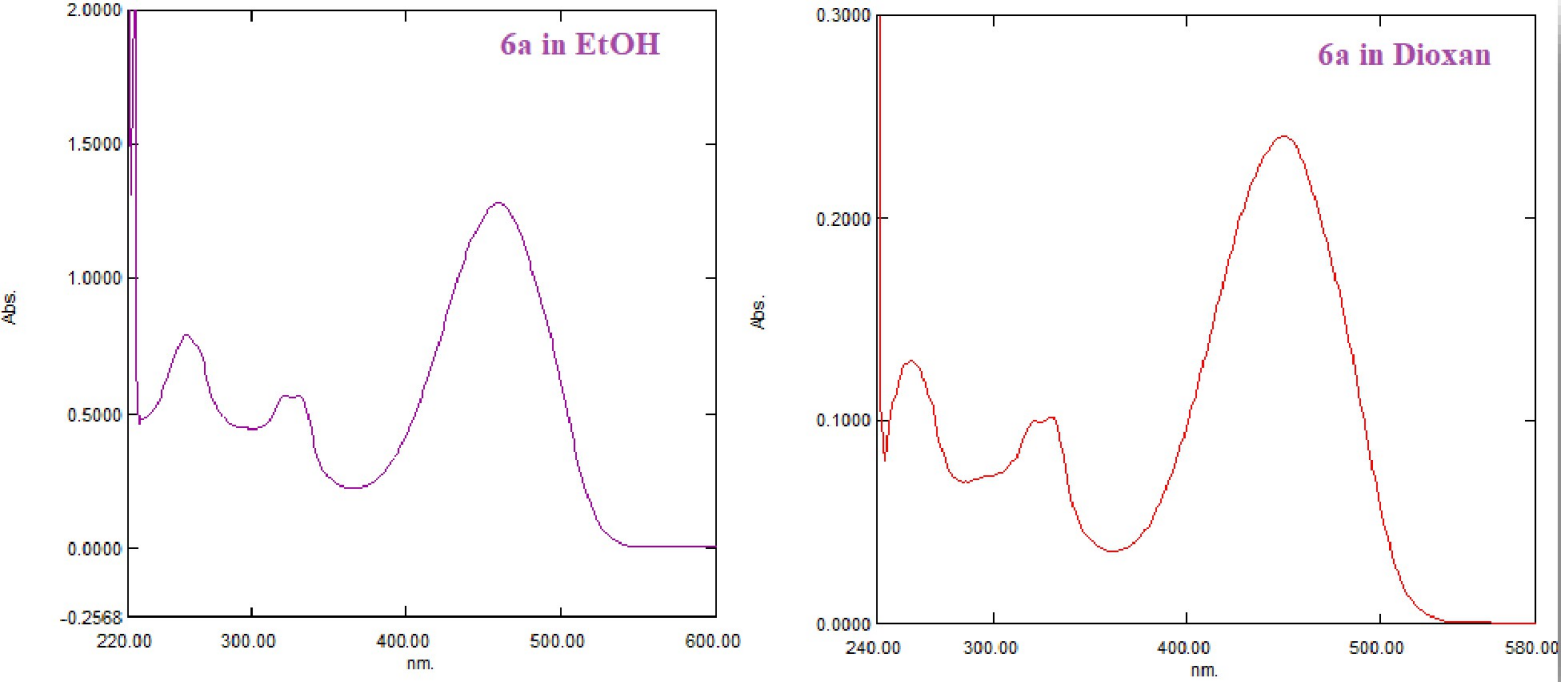
The UV spectra of thiazole derivative 6a in ethanol and dioxane.

**Scheme 2:**
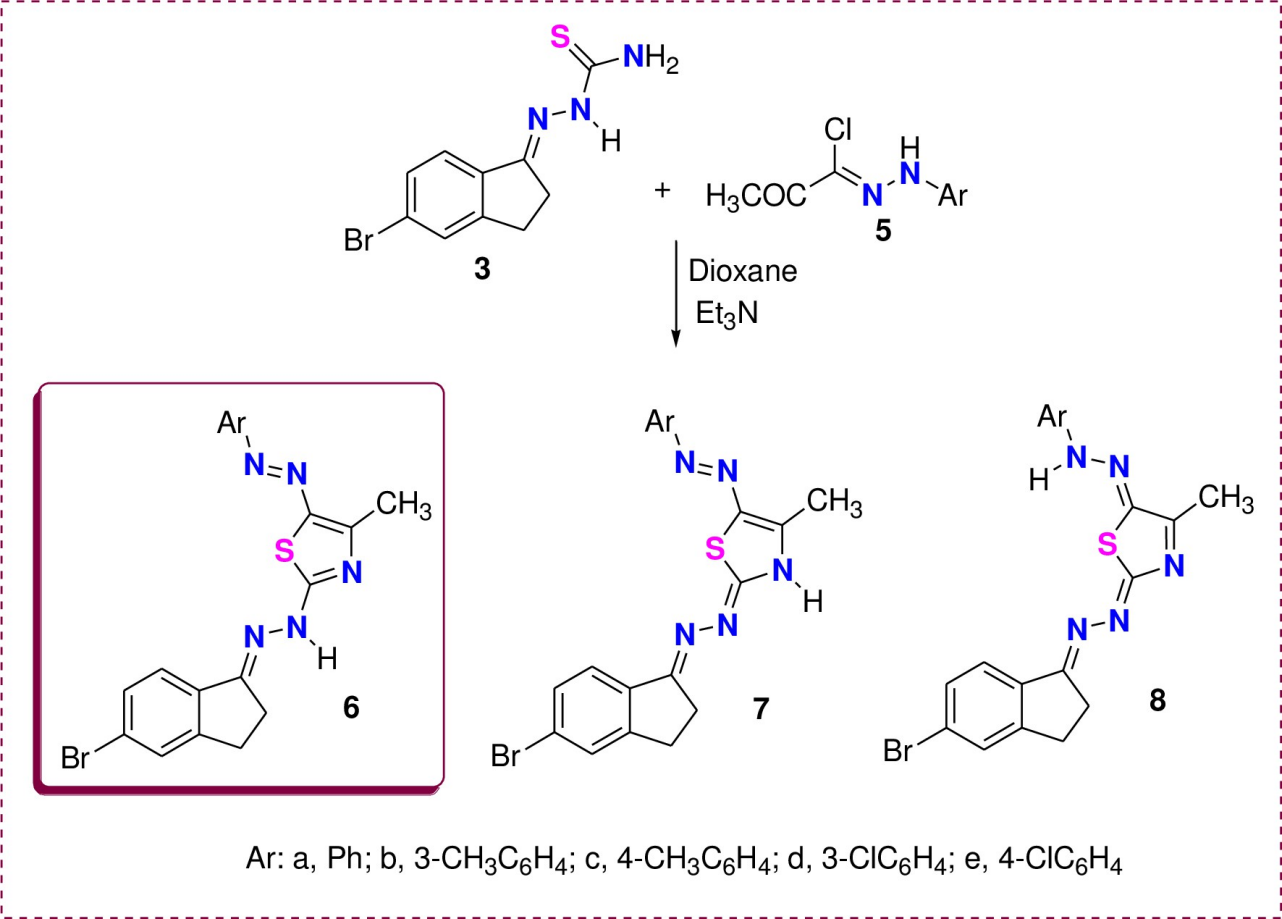
Synthesis of thiazole derivatives 6a-e.

To synthesize a new series of thiazole rings combined with indene, we subjected the thiosemicarbazone derivative **3** to react with hydrazonoyl chloride **5a-e** in dioxane/Et_3_N under reflux (Scheme 2). Such reaction products can be found in one of the three tautomeric forms 6–8. Based on the wavelength (λ_max_) of the product in dioxane revealed for all derivatives, three absorption bands at 458–431, 331–323, and 259–241 nm (Table 1 & Fig 3). The appearance of λ_max_ at 458-431nm excluded the hydrazone tautomer 8 and proved the existence of one of the two azo-form **6** or **7** [32]. Double irradiation of the CH_3_ group of thiazole derivative **6a** or **7a** at δ = 2.69 ppm in the NOE difference experiment revealed no enhancement for the NH signal, which means there is no electronic interaction between the protons of CH_3_ and NH groups [57]. This result confirmed the tautomeric form **6** rather than structure **7**. Recording the electronic absorption of the unsubstituted thiazole derivative **6a** in different solvents with different polarities (Table 1), we noted no change in the absorption bands in three solvents (ethanol, acetone, and acetonitrile). Still, there is a strong bathochromic shift in the case of DMF and DMSO. This shift indicated the derivative 6a in two tautomers, **6a** and **7a,** in the high dielectric solvents DMF and DMSO.

**Table 1:** UV Spectral data of compounds 6a-e in dioxane and thiazole 6a in different solvents.

Compd. No.	λ_max_ (log ε)
**6a**	450 (4.18); 330 (3.80); 241 (4.65)
**6b**	453 (4.71); 323 (4.44); 258 (4.53)
**6c**	458 (4.81); 326 (4.44); 244 (4.42)
**6d**	431 (4.54); 329 (4.31); 255 (4.47)
**6e**	450 (5.04); 331 (4.64); 259 (4.77)
**6a (different solvents)**	Acetone: 449 (4.85); 327 (4.45);
	EtOH: 460 (4.90); 331 (4.55); 258 (4.69);
	CH_3_CN: 449 (4.86); 269 (499);
	DMF: 592 (4.41); 458 (5.04); 332 (4.54);
	DMSO: 592 (4.28); 463 (4.73); 334 (4.41)

**Fig 4:**
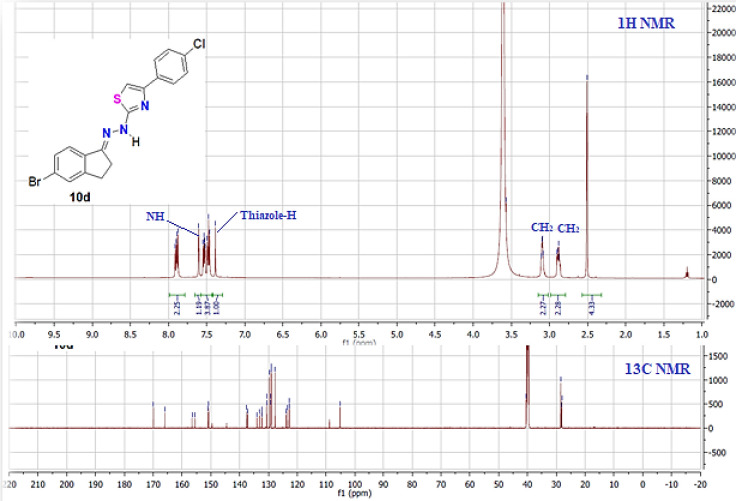
The ^1^H & ^13^C NMR spectra of thiazole derivative 10d.

**Scheme 3:**
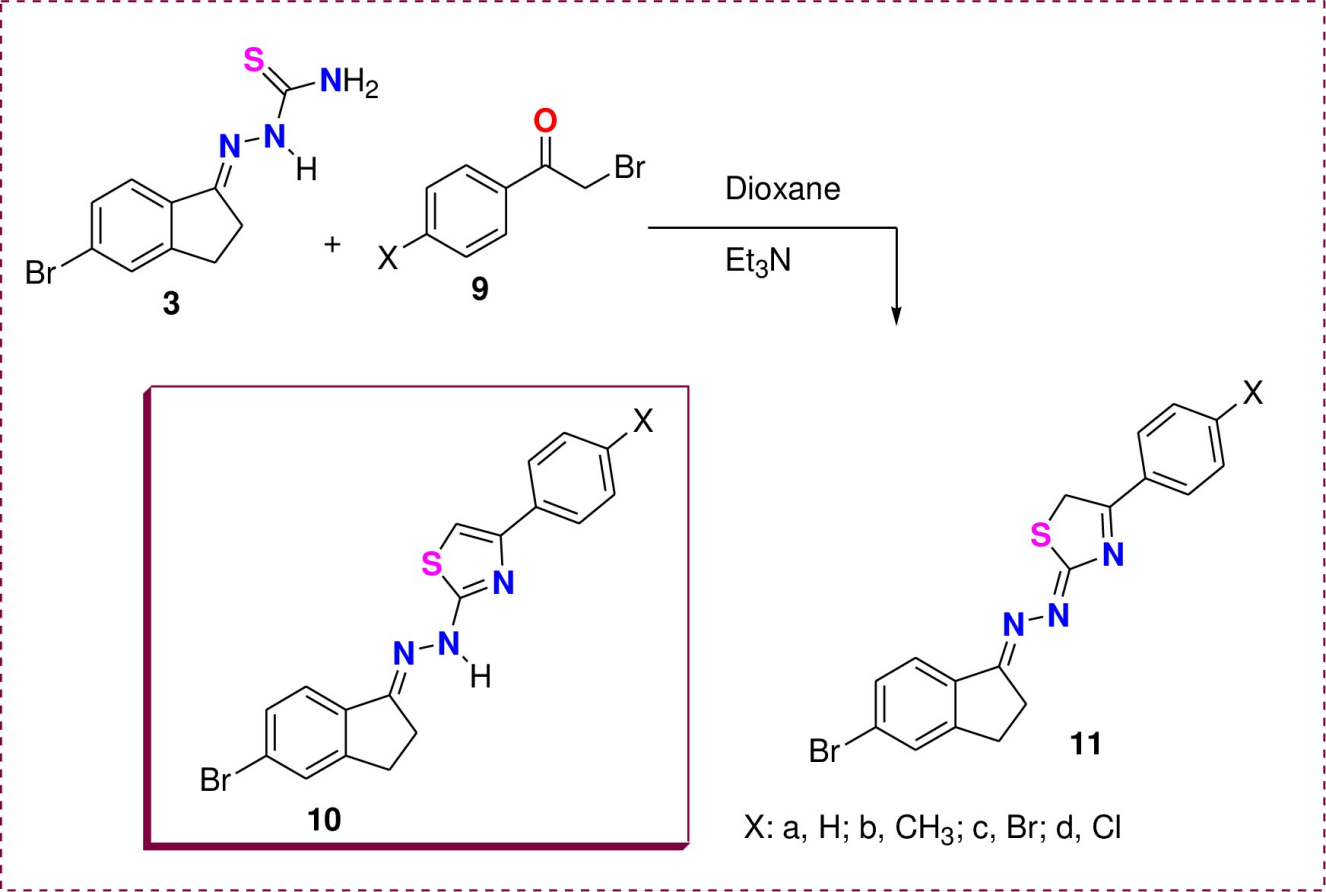
Synthesis of thiazole derivatives 10a-d.

Similarly, phenacyl bromide derivatives **9a-d** were reacted with thiosemicarbazone derivative **3** to afford the thiazole derivatives **10** or **11**, as illustrated in Scheme 3. However, spectral analysis data proved the isolated product’s structure as **10** rather than **11**. This conclusion was observed from the ^1^H & ^13^C NMR data (Fig 4). For instance, the ^1^H NMR spectra of thiazole derivative **10d** showed the singlet signal for the thiazole-H rather than the CH_2_ of thiazole in isomer **11**. In addition, the ^13^C NMR (Fig 4) was completely devoid of thiazole-CH_2_ in the aliphatic region, but it revealed 14 carbon signals located in the aromatic region from 105 to 170 ppm.

**Scheme 4:**
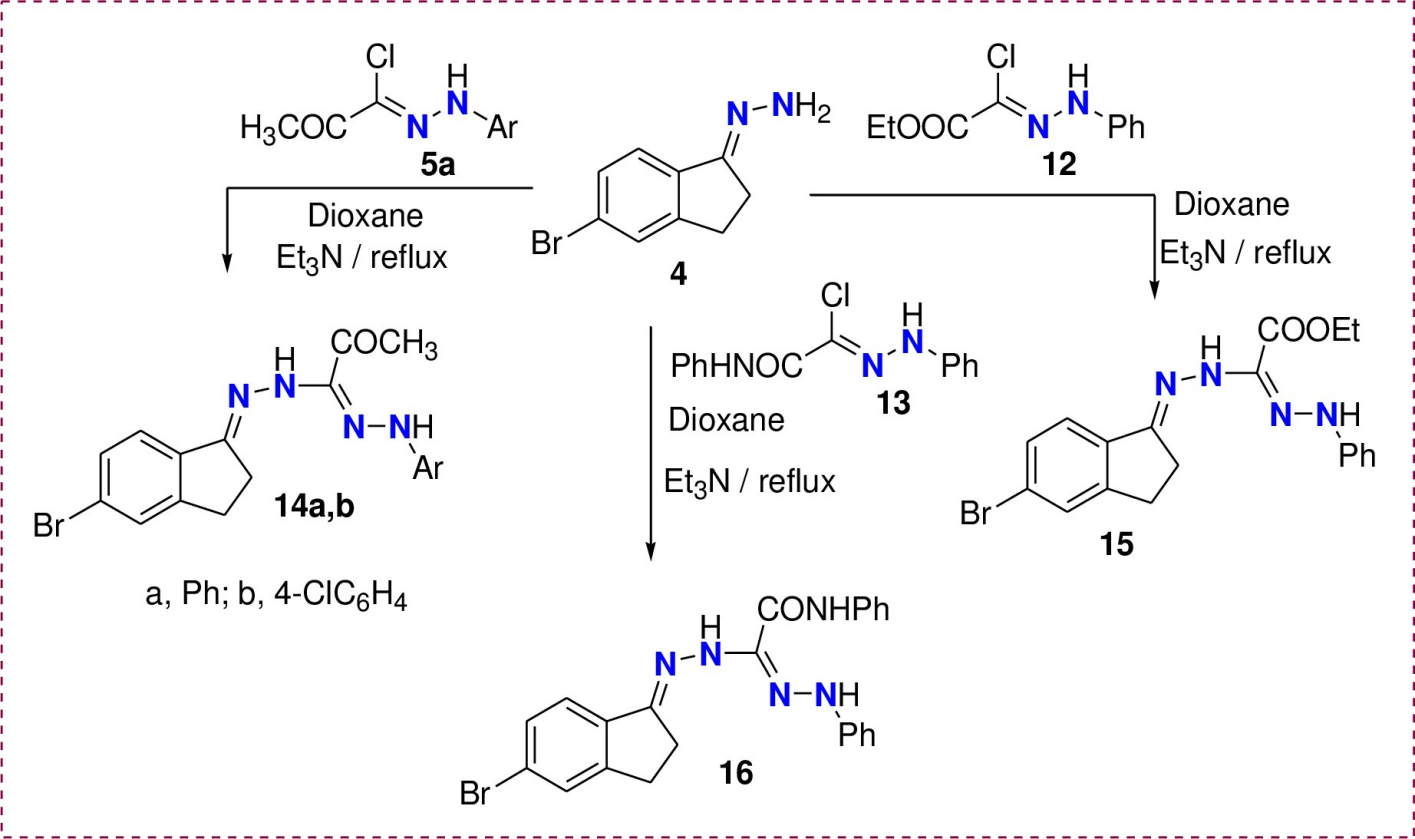
Synthesis of Formazan derivatives 14a, b, 15 and 16.

Finally, conversion of the (5-Bromo-indan-1-ylidene)-hydrazine (**4**) to the formazan derivatives was achieved via the reaction of **4** with hydrazonoyl chloride **5a**, **12,** and **13** in dioxane/Et_3_N under reflux for 5h (Scheme 4). As a result, the formed formazan derivatives **14–16** were assured based on all possible spectral techniques (See Experimental part).

### 3.2. XRD analysis

**Fig 5:**
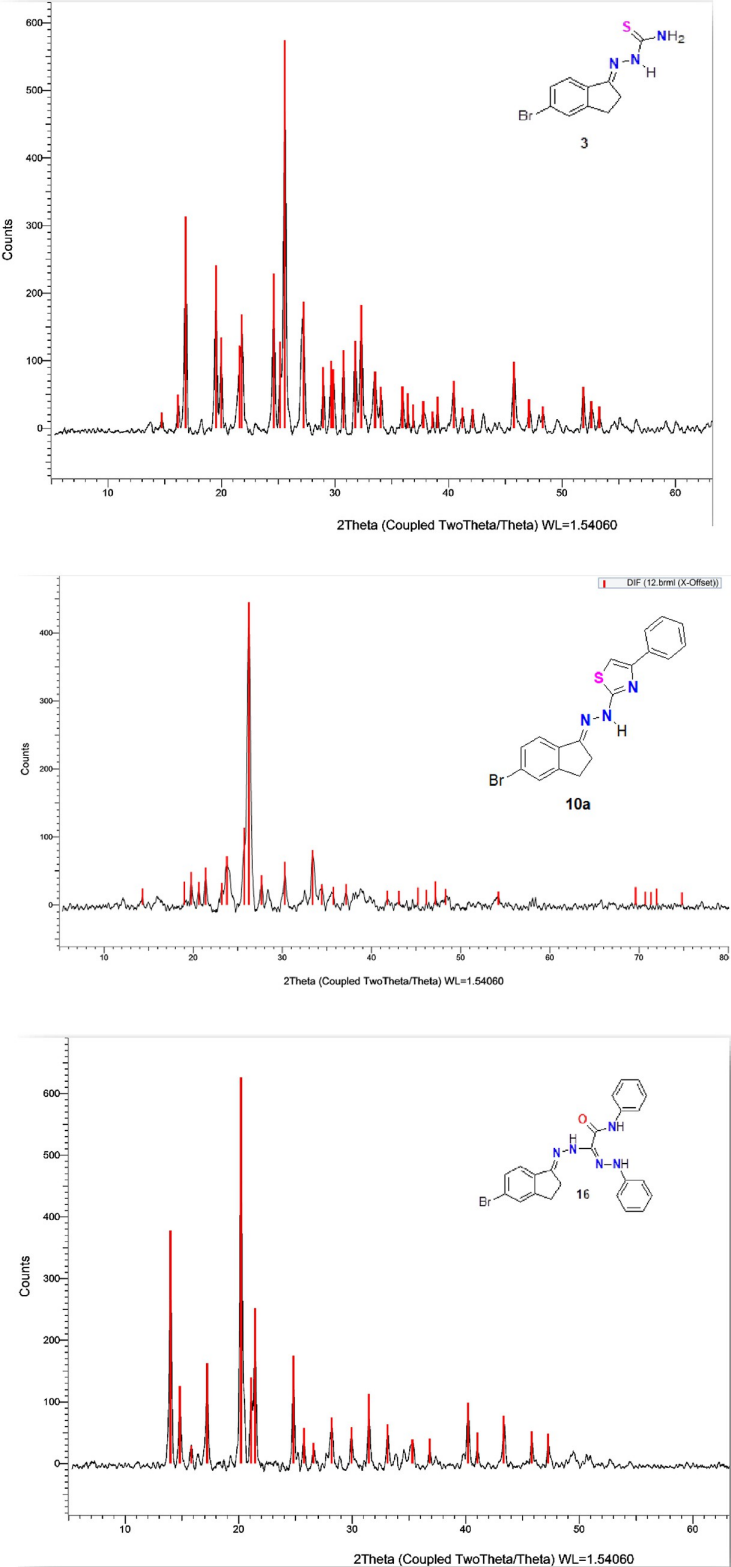
The XRD chart for compound 3. **a**. **4b**. The XRD chart for compound **10a. 4c**. The XRD chart for compound **16.**

The XRD of all synthesized thiosemicarbazone derivative **3**, hydrazone **4**, thiazole derivatives **6a-e, 10a-d**, and formazan derivatives **14a, b, 15,** and **16** were recorded over 10° *<* 2θ *<* 80° range to evaluate their crystallographic features (Fig 5A–5C). Only five derivatives, **3**, **10a**, **14a**, **15**, and **16**, have crystalline shapes due to the appearance of sharp peaks in their XRD charts. In contrast, the other derivatives were amorphous. Debye–Scherrer equation [58] was used to calculate the size of the crystals of the five derivatives **3**, **10a**, **14a**, **15,** and **16,** and the date was listed in Table 2. The results indicated that all five derivatives were synthesized on the nanometre scale.

**Table 2:** XRD parameters for nano-crystalline 3, 10a, 14a, 15 and 16 derivatives.

Compounds	Size (nm)	2θ	Intensity	FWHM
**3**	7.766	25.5	565	0.1023
**10a**	3.372	26.1	440	0.2356
**14a**	3.10	26.8	240	0.2562
**15**	8.0	27.0	515	0.0998
**16**	7.71	20.2	620	0.1030

### 3.3 Biological studies

#### Anticancer screening

The scenario of cancer disease is one of the most challenging diseases around the world significantly. Colon and gastric cancer are familiar types in the middle east. Gastric cancer is the third deadliest tumor malignancy worldwide. A new series of substituted thiazoles were tested against two cell lines, **SNU-16** for gastric carcinoma and **Colo205** for colon carcinoma, with two benchmarks, cis-Pt and Sunitinib. Sunitinib is an effective drug for treating both colon and gastric cancer. The mechanism of action of cisplatin has been linked to its ability to crosslink with the purine bases on the DNA, interfering with DNA repair mechanisms, causing DNA damage, and subsequently inducing apoptosis in cancer cells [44].

**Fig 6:**
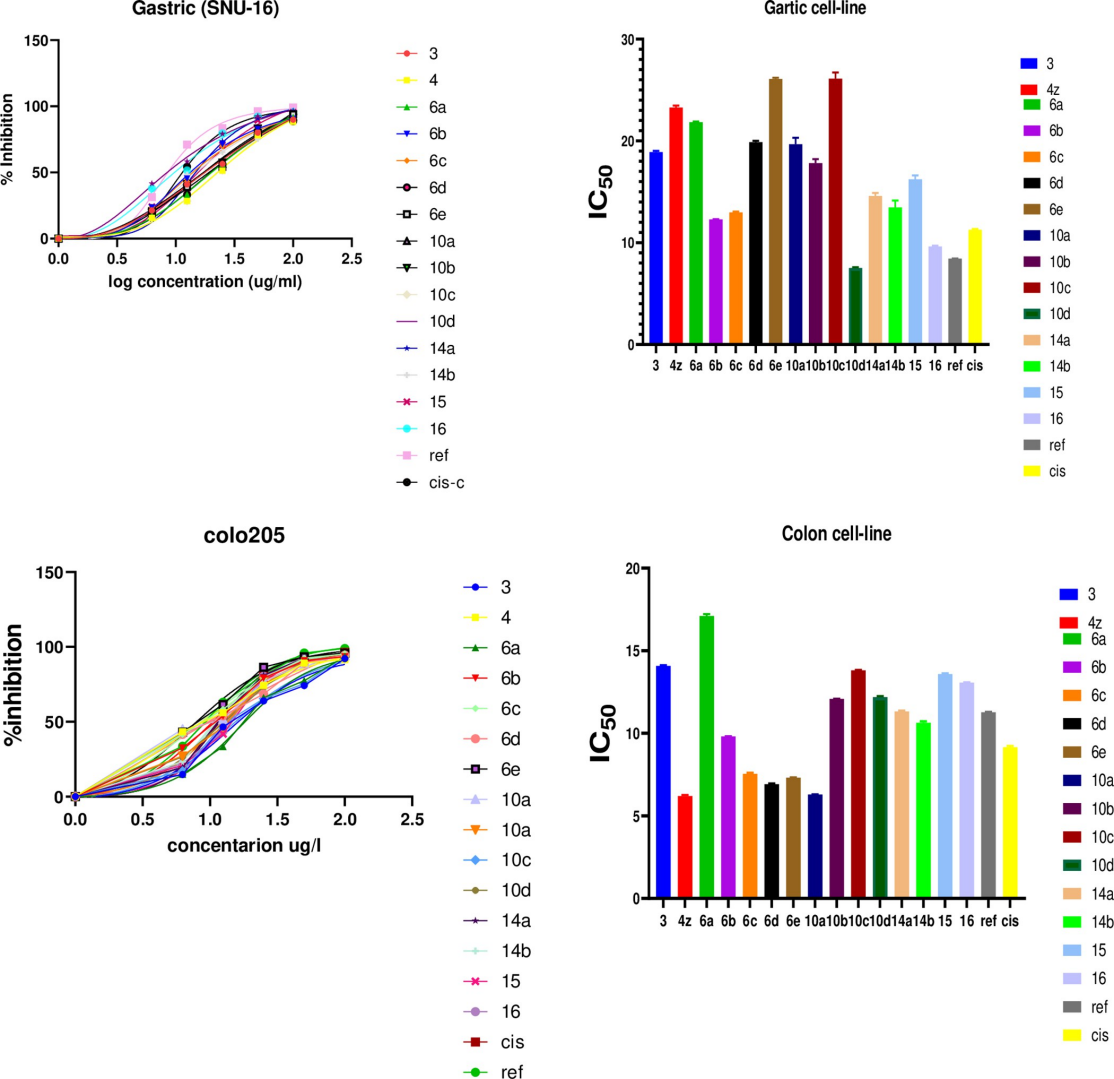
Anti-gastric and anti-colon activities for the tested derivatives.

For SNU-16, from the results obtained in Table 3 & Fig 6, compound 10d represented an excellent IC50 result better than the two benchmarks used, while compound **16** showed IC_50_ better than cis-platin and near Sunitinib. For Colo205, IC_50_ values of compounds **14a** and **14b** are better than cis-pt values, while IC_50_ for compounds **4**, **6c**, **6d**, **6e**, and **10a** are better than the two benchmarks used. The results obtained can be explained in detail and proved by pharmacophore studies. Apoptosis is an essential phenomenon in cytotoxicity induced by anticancer drugs. It is apparent that the molecular mechanisms by which anticancer drugs induce apoptosis are mediated by death receptor-dependent and -independent pathways, which are related to the release of cytochrome c through voltage-dependent anion channels in the mitochondrial inner membrane. The release of cytochrome c is the central gate in turning on/off apoptosis. It is regulated by the interaction of proapoptotic proteins, including Bid, Bax, and Bak, and anti-apoptotic proteins, including Bcl-2 and Bcl-XL, and a specific class of inhibitors of apoptosis proteins (IAPs) including Akt, survivin, and heat-shock proteins. Drug sensitivity can be enhanced by the introduction of proapoptotic genes and the inhibition of anti-apoptotic proteins. The signal transduction pathways triggered by the central gate in mitochondria play a critical role in anticancer drug-induced apoptosis. The modulation of signal transduction pathways targeting the proteins involved in these signal transduction pathways using antisense IAPs, and growth factor antibodies may be a good strategy for enhancing the therapeutic efficacy of anticancer drugs in cancer chemotherapy [46–48].

**Table 3:** The IC_50_ of the anti-gastric and anti-colon cancer activities of the synthesized derivatives 3, 4, 6a-e, 10a-d, 14a, b, 15 and 16.

Sample	IC_50_ uM Gastric (SNU-16) Patch number (ATCC CRL-5822)	IC_50_ uM Colon / (COLO205)	IC_50_ uM	SI
**3**	19.01 ± 0.133	14.10 ± 0.04	> 100	---
**4**	23.20 ± 0.204	8.167 ± 0.07	> 100	---
**6a**	21.79 ±0.076	17.14 ± 0.12	> 100	---
**6b**	12.28 ± 0.03	9.813 ± 0.005	> 100	---
**6c**	12.89 ± 0.076	7.576 ± 0.07	> 100	---
**6d**	19.79 ±0.129	6.958±0.04	605.27 ± 35.21	86.98
**6e**	26.19 ± 0.12	7.276 ± 0.03	> 100	---
**10a**	19.96 ± 0.64	6.301±0.008	484.61 ± 7.89	76.82
**10b**	18.27 ± 0.39	12.08 ± 0.006	> 100	---
**10c**	26.80 ± 0.6	13.81 ± 0.025	> 100	---
**10d**	7.576±0.075	12.16 ± 0.06	563.07 ± 44.19	74.32
**14a**	14.90 ± 0.3	11.27 ± 0.046	> 100	---
**14b**	13.81 ± 0.69	10.61 ± 0.09	> 100	---
**15**	16.64 ± 0.38	13.63 ± 0.04	> 100	---
**16**	9.699±0.0726	13.04 ± 0.025	524.93± 36.18	54.13
**sunitinib**	8.450 ± 0.023	11.27 ± 0.033	378.50 ± 31.84	---
**Blank ref Cisplatin**	11.27± 0.076	9.130 ± 0.07	548.13 ± 61.18	---

The compounds show good selectivity toward cancerous cells, represented by the “selectivity index.” The selectivity index was previously used to measure selective cytotoxic activity [50]. The selectivity index (SI) can be calculated from the ratio between IC50 of the compound on a normal cell and the compound on a cancer cell line. For example, the SI of compounds 6d, 10a, 10d, and 16 are 86.98, 76.82, 74.32, and 54.13, respectively.

### 3.4 Computational Insilico-studies

#### A-Molecular docking

**Fig 7:**
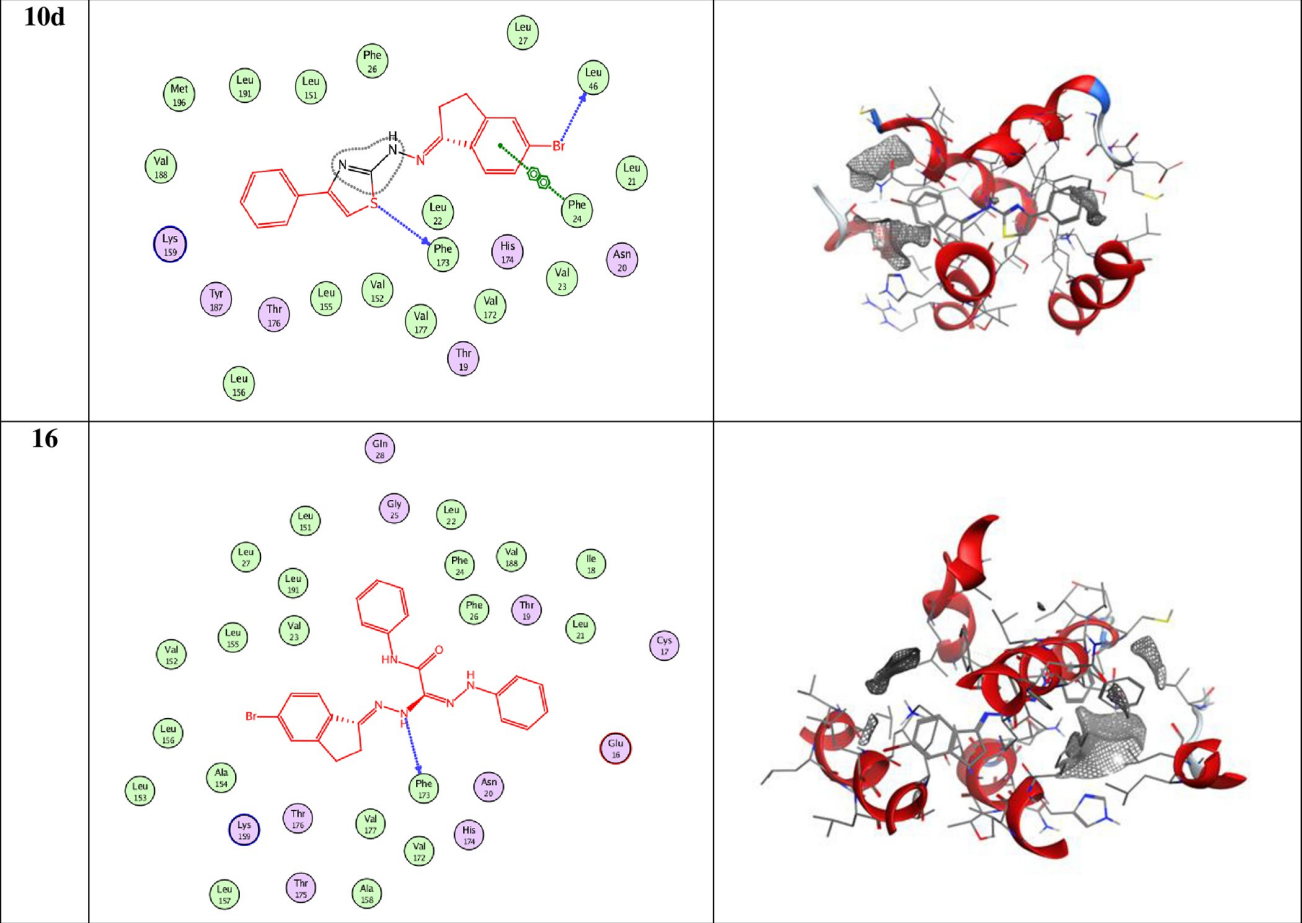
2D and 3D snapshots showing the hydrophilicity interaction to Gastric (SNU-16) Gastric cancer (2BID) receptor.

Colon and gastric carcinoma are now the most widely spread types of cancer. Therefore, molecular docking studies were performed for selected compounds. According to the results obtained from biological studies, compounds **10d** and **16** have been chosen to carry out molecular docking studies on gastric cancer (PDB = **2BID**) and colon cancer (PDB = **2A4L**). According to the literature, the two proteins were selected [46, 47, 49]. Fig 7 explains 2D and 3D snapshots of the hydrophilicity interaction to **PDB = 2BID** and the results obtained for Gastric cancer (**PDB = 2BID**) receptor. The docking score energies for compounds **10d** and **16** are -5.95 and -6.446 kcal/mol, respectively (Table 4). Furthermore, compound **10d** interacts from BR18 with O-LEU46(A) by hydrogen donor. Also, it interacted by S26 with OPHE173(A) by hydrogen donor, and finally by 6-ring with 6-ring PHE24(A) by pi-pi interactions (Table 5).

**Table 4:** Docking score and energies of compounds with Gastric (SNU-16) Gastric cancer (2BID) receptor.

Comp.	S	rmsd_refine	E_conf	E_place	E_score1	E_refine	E_score2
**10d**	-5.95	3.27	58.18	-42.83	-8.820	-23.632	-5.95
	-5.75	2.47	55.72	-57.78	-9.173	-29.642	-5.75
	-5.65	1.15	65.94	-51.168	-8.379	-21.93	-5.65
	-5.48	1.71	56.706	-27.76	-8.405	-18.86	-5.48
	-5.34	1.69	67.41	-44.112	-8.521	-23.63	-5.34
**16**	-6.446	1.692	106.16	-69.134	-9.549	-30.241	-6.446
	-6.397	1.592	99.397	-63.030	-8.276	-33.228	-6.397
	-6.238	1.631	103.751	-58.854	-8.854	-35.050	-6.238
	-6.232	2.120	101.603	-65.155	-9.013	-33.661	-6.232
	-6.083	2.375	102.335	-34.123	-8.674	-29.870	-6.083

**Table 5:** Docking interaction of all compounds Gastric (SNU-16) Gastric cancer (2BID) receptor.

Compound	Ligand	Receptor	Interaction	Distance E	(kcal/mol)
**10d**	BR 18	O LEU 46 (A)	H-donor	2.88	-3.3
	S 26	O PHE 173 (A)	H-donor	3.71	-1.2
	6-ring	6-ring PHE 24 (A)	pi-pi	3.96	-0.0
**16**	N 19	O PHE 173 (A)	H-donor	3.44 -	-1.1
	N 28	6-ring PHE 26 (A)	H-pi	3.38	-0.5

**Fig 8:**
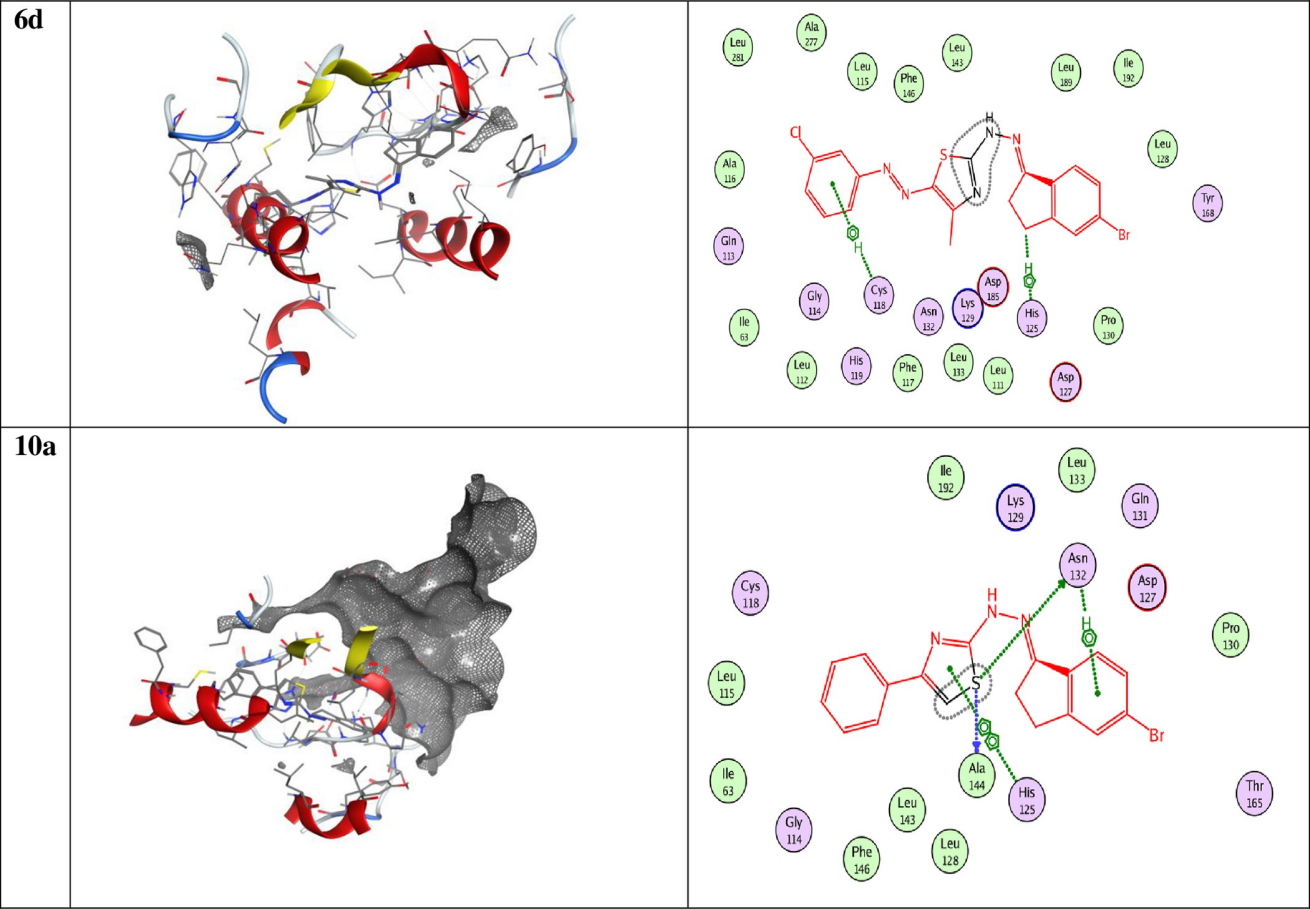
2D and 3D snapshots showing the hydrophilicity interaction to colo205 (PDB = 2A4L).

Colon cancer from biological studies, compounds **6d** and **10a** have chosen to carry out molecular docking studies on colon cancer (PDB = **2A4L**), (Fig 8) explain 2D and 3D snapshot of the hydrophilicity interaction to **PDB = 2A4L** the results obtained for Colon cancer (**PDB = 2A4L**) receptor. The docking score energies for compounds **6d** and **10a** are -7.279 and -6.63 kcal/mol, respectively (Table 6). In addition, compound **6d** interacts from C10 with 5-ring HIS 125 (A) by H-pi. It was also interacted by 5-ring HIS 125 (A)N CYS 118 (A) by pi-H. At the same time, compound 10a interacted from 6-ring at N-ASN132 (A) by pi-H and from 5-ring at 5-ring HIS 125 (A) by pi-pi (Table 7).

**Table 6:** Docking score and energies of compounds with Colo205 (PDB = 2A4L).

Comp.	S	rmsd_refine	E_conf	E_place	E_score1	E_refine	E_score2
**6d**	‐7.279	1.447	82.160	‐61.66	‐9.826	‐41.907	‐7.279
	‐6.962	0.956	78.173	‐72.401	‐11.326	‐36.515	‐6.962
	‐6.890	2.030	47.680	‐92.803	‐10.254	‐39.582	‐6.890
	‐6.746	1.430	80.166	‐71.021	‐10.049	‐29.935	‐6.746
	‐6.697	1.558	53.510	‐78.718	‐10.571	‐41.219	‐6.697
**10a**	‐6.613	3.038	65.314	‐63.873	‐10.547	‐33.843	‐6.613
	‐6.475	1.508	58.529	‐58.031	‐10.020	‐27.089	‐6.475
	‐6.452	1.384	65.491	‐78.712	‐10.125	‐32.785	‐6.452
	‐6.245	1.425	55.781	‐67.577	‐9.897	‐32.404	‐6.245
	‐6.234	1.533	63.883	‐63.923	‐10.094	‐32.176	‐6.234

**Table 7:** Docking interaction of all compounds Gastric (SNU-16) Gastric cancer (2BID) receptor.

Compound	Ligand	Receptor	Interaction	Distance E	(kcal/mol)
**6a**	C 10	5-ring HIS 125 (A)	H-Pi	3.60	-1.0
	5-ring	N CYS 118 (A)	Pi-H	4.22	-1.1
**10a**	6-ring	N ASN 132 (A)	pi-H	4.30	-1.3
	5-ring	5-ring HIS 125 (A)	pi-pi	3.65	-0.0

#### B-Pharmacophore and ADME studies

The predicted pharmacokinetic/Molinspiration properties of the new starting thiosemicarbazone, hydrazone, thiazole, and formazan derivatives **3–16**, are given in Tables 8 & 9. With the help of Molinspiration virtual screening, most of the tested compounds showed good bioactivity as indicated from docking studies in Tables 4–7, which indicates the drug-likeness properties against kinase inhibitor, protease, and enzyme inhibitors.

**Table 8:** Physicochemical properties of the synthesized compounds.

Compd.	miLogP	TPSA	n-atoms	MW	nON	nOHNH	n-violations	nrotb	Volume
**3**	2.47	50.41	15	284.18	3	3	0	2	199.50
**4**	2.28	38.39	12	225.09	2	2	0	2	159.24
**6a**	5.94	62.01	26	426.34	5	1	1	4	325.39
**6b**	6.37	62.01	27	440.37	5	1	1	4	341. 95
**6c**	6.39	62.01	27	440.37	5	1	1	4	341. 95
**6d**	6.59	62.01	27	460.79	5	1	1	4	338.93
**6e**	6.62	62.01	27	460.79	5	1	1	4	338.93
**10a**	5.08	37.28	23	384.30	3	1	1	3	289.33
**10b**	5.53	37.28	24	398.33	3	1	1	3	306.29
**10c**	5.88	37.28	24	463.2	3	1	1	3	307.61
**10d**	5.75	37.28	24	418.75	3	1	1	3	303.26
**14a**	5.11	65.85	25	385.26	5	2	1	5	309.7
**14b**	5.79	65.85	25	419.71	5	2	1	5	316.5
**15**	5.80	75.09	26	415.29	6	2	1	7	328.76
**16**	6.37	77.88	30	462.35	6	3	1	6	370.22

**Table 9:** Physicochemical Molinspiration bioactivity score.

Compound	GPCR ligand	Ion channel modulator	Kinase inhibitor	Nuclear receptor ligand	Protease inhibitor	Enzyme inhibitor
**3**	-1.13	-0.91	-1.15	-1.43	-1.11	-0.29
**4**	-0.75	-0.94	-1.02	-1.04	-1.57	-0.34
**5**	-0.47	-0.67	-0.25	-1.03	-0.78	-0.19
**6a**	-0.49	-0.70	-0.27	-1.02	-0.80	-0.24
**6b**	-0.48	-0.69	-0.27	-1.02	-0.80	-0.22
**6c**	-0.48	-0.69	-0.27	-1.02	-0.80	-0.22
**6d**	-0.47	-0.65	-0.26	-1.02	-0.83	-0.23
**6e**	-0.46	-0.65	-0.26	-1.01	-0.80	-0.21
**10a**	-0.38	-0.65	-0.23	-0.79	-0.69	-0.07
**10b**	-0.41	-0.7	-0.26	-0.78	-0.71	-0.31
**10c**	-0.36	-0.62	-0.22	-0.75	-0.65	-0.07
**10d**	-0.37	-0.63	-0.24	-0.77	-0.69	-0.10
**14a**	-0.21	-0.54	-0.60	-0.70	-0.56	-0.27
**14b**	-0.20	-0.52	-0.60	-0.69	-0.58	-0.29
**15**	-0.28	-0.52	-0.56	-0.66	-0.53	-0.30
**16**	-0.18	-0.44	-0.37	-0.62	-0.42	-0.23

The Calculated distribution of activity scores (version 2011.06) for GPCR ligands, kinase inhibitors, ion channel modulators, nuclear receptor ligands, protease inhibitors, and other enzyme targets compared with scores for about 100’000 average drug-like molecules. The score allows efficient separation of active and inactive molecules [52–57].

**Fig 9:**
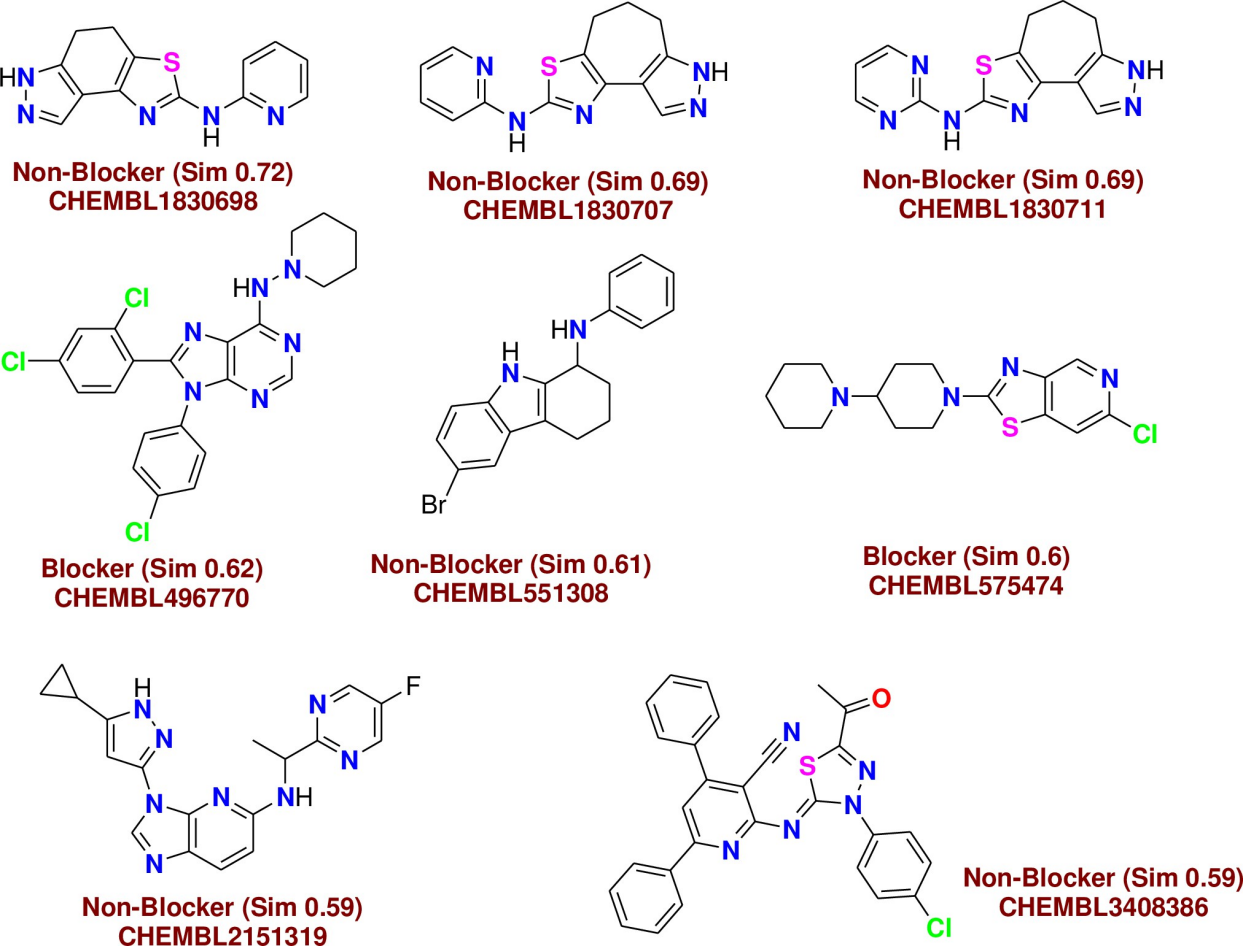
Similar off-target compounds of compound 10d.

**Fig 10:**
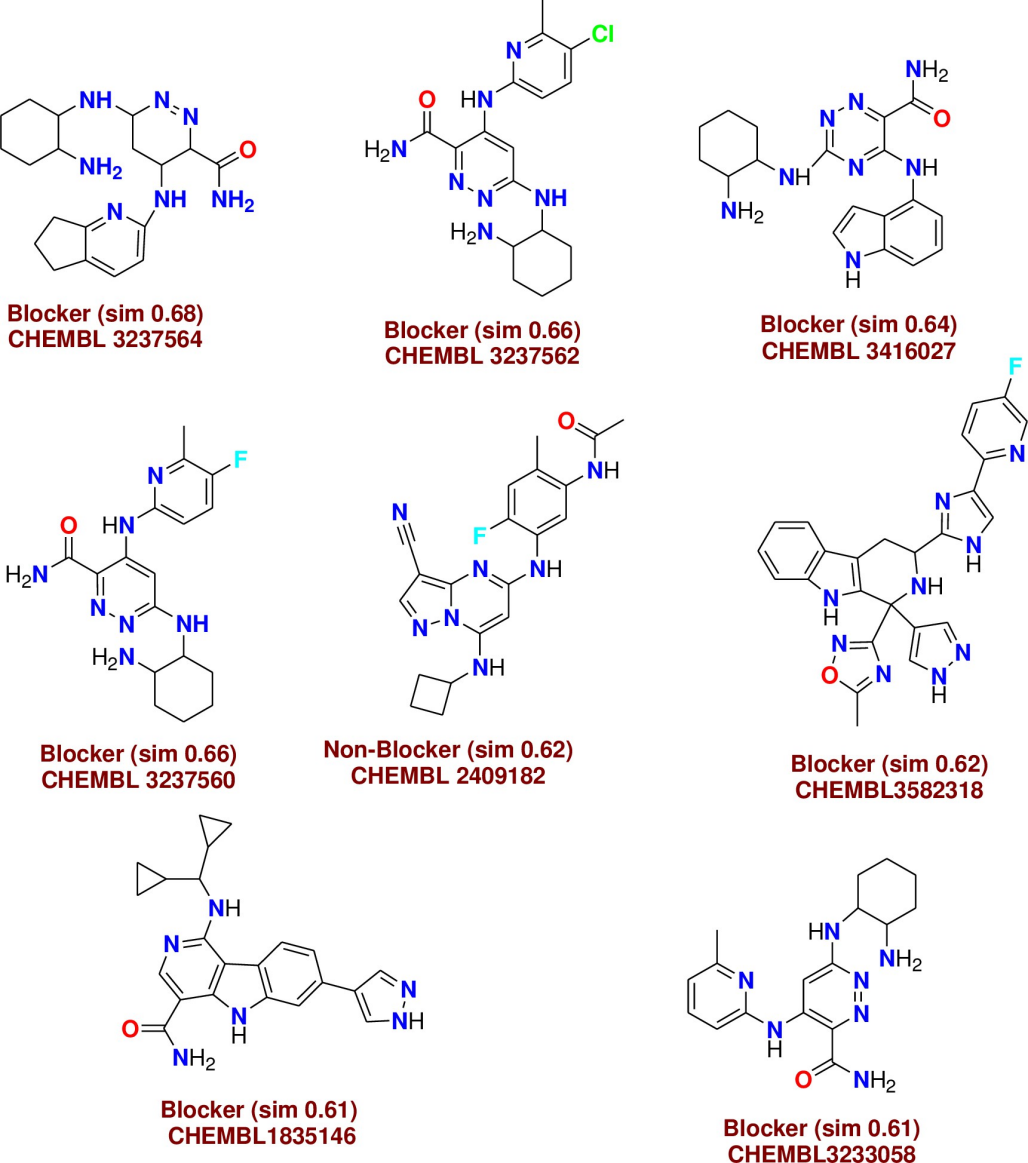
Similar off-target compounds of compound 16.

**Fig 11:**
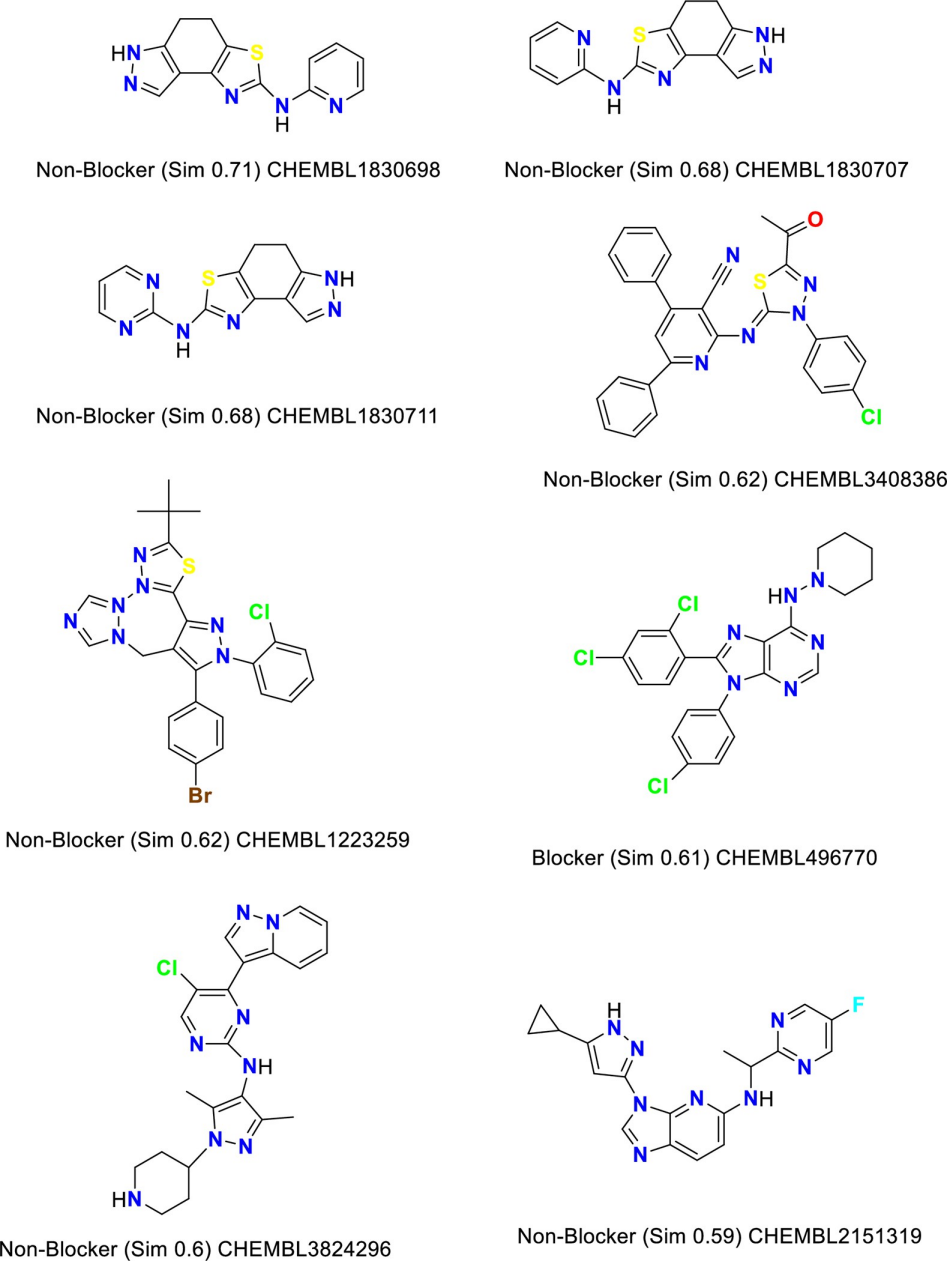
Similar off-target compounds of compound 6d.

**Fig 12:**
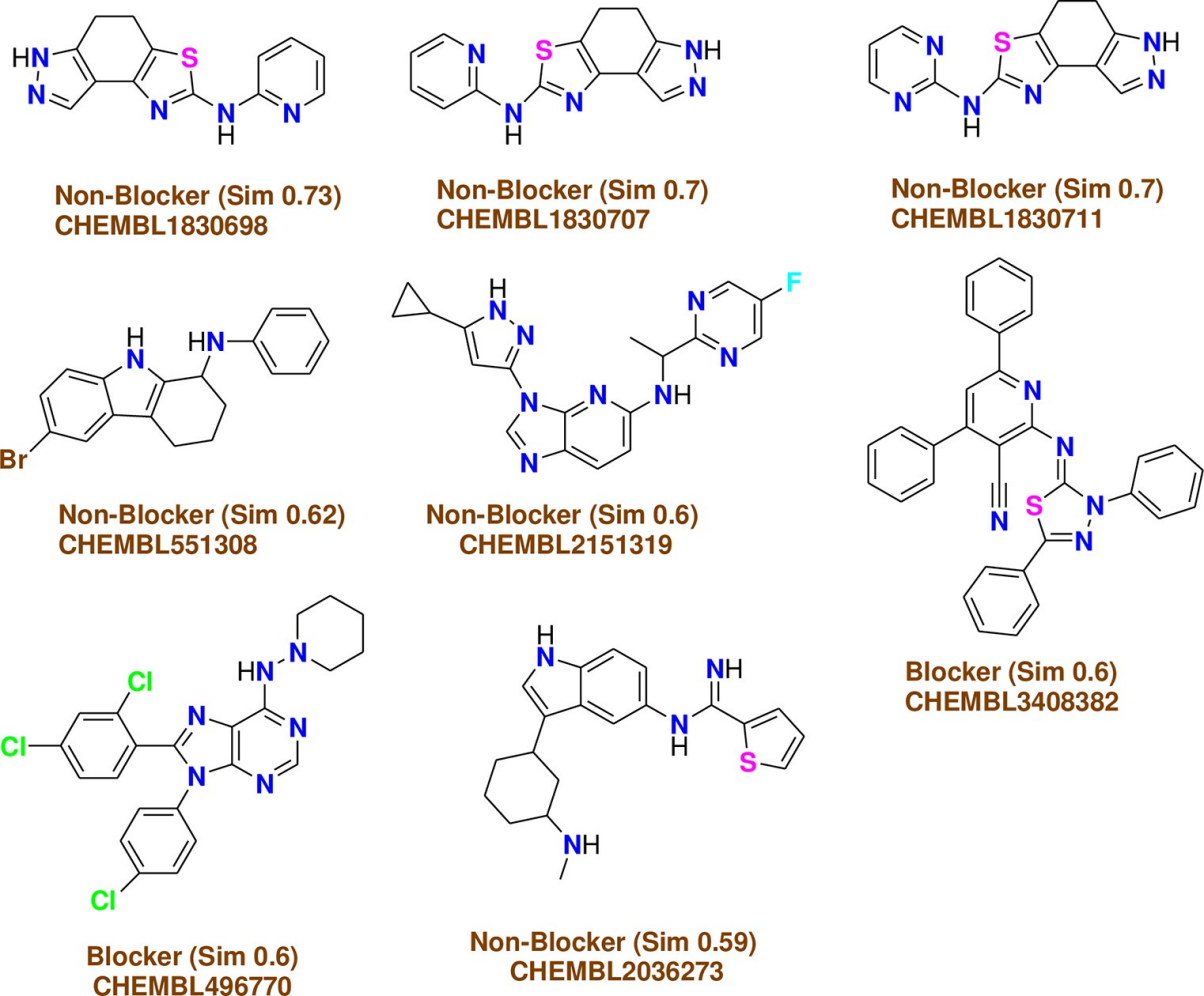
Similar off-target compounds of compound 10a.

**Fig 13:**
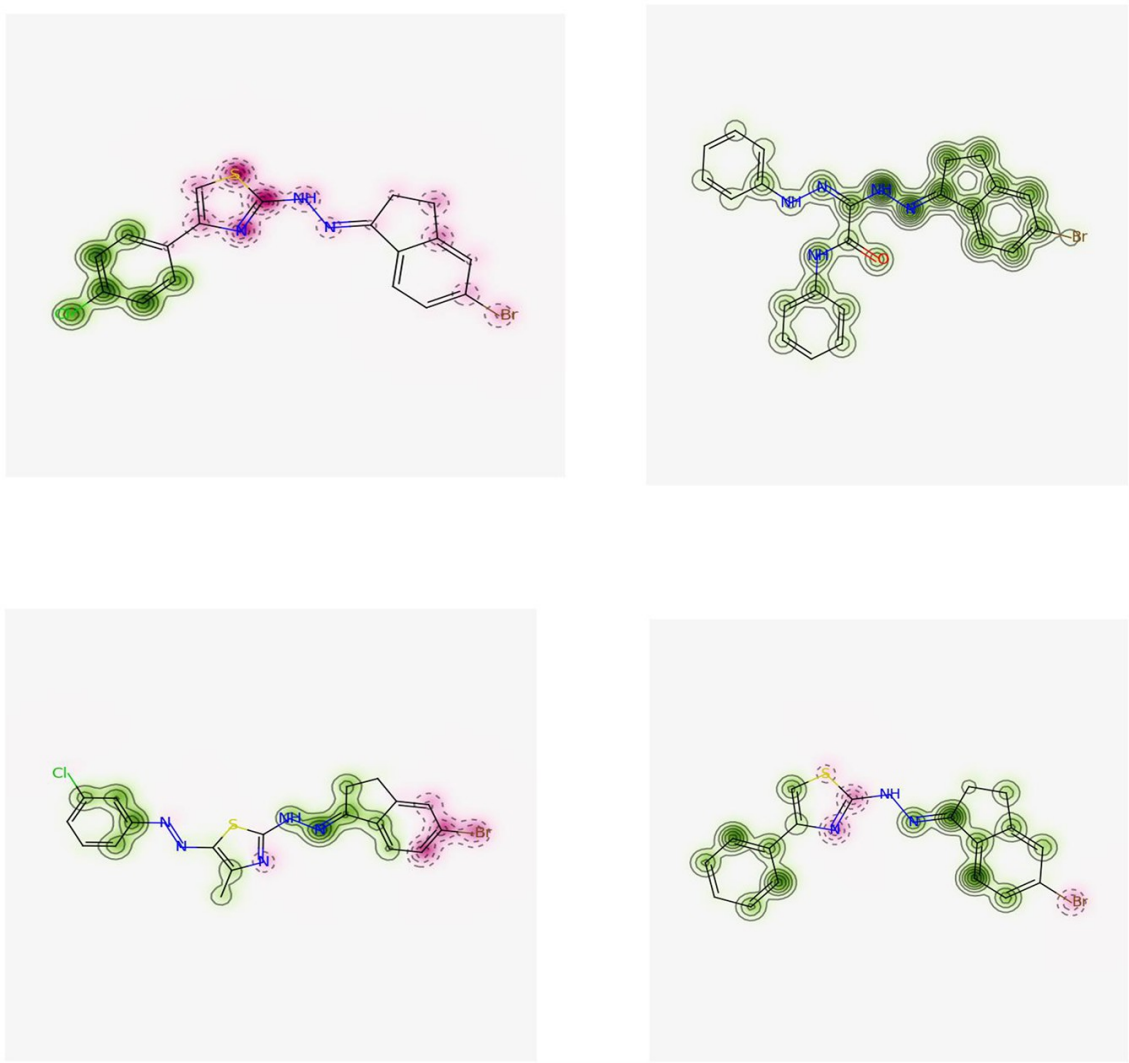
Interpretation of probability of toxicity for compounds 10d, 16, 6d, and 10a.

*C-1- Pred-hERG*. Chemically similar compounds often bind biologically diverse protein targets, and protein structures do not always recognize identical ligands. Pharmacological and off-target relationships among proteins and a ligand set similarity help to improve the machine learning confidence by interpolating the output prediction equalized by the compound similarity criteria. This pipeline help to improve the predictions of off-target drug effects, reducing the false-negative error. The chemical similarity is one of the essential concepts in cheminformatics. One commonly used to calculate these similarity algorithm measures is the 2D Tanimoto algorithm. The resulting Tanimoto coefficient is fingerprint-based, encoding each molecule to a fingerprint "bit" position (MACCS), with each bit recording the presence ("1") or absence ("0") of a fragment of the molecule (Figs 9–13).

Probability map of HERG of **10d**, **16**, **6d**, and **10a**. The more depth lines and the more intense green color imply a higher positive contribution of an atom or a fragment to the hERG blockage, while pink means that it contributes to decreasing hERG blockage and gray means no contribution.

**Fig 14:**
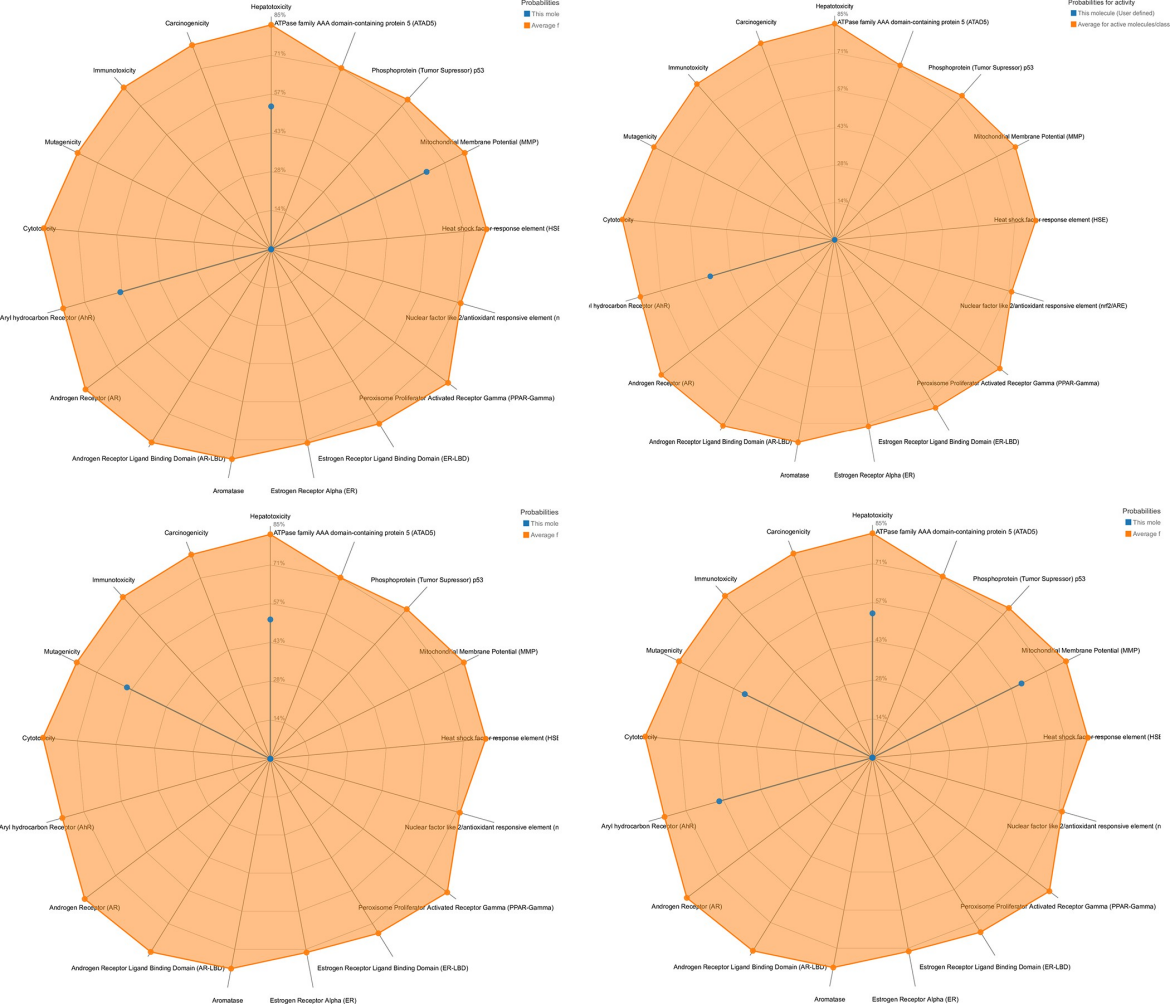
Toxicity radar for compounds 10d, 16, 6d and 10a.

*C-2*. *Pro-ToxII*. The ProTox-II (Fig 14 & Table 10) showed that the four compounds are predicted to have oral LD_50_ values ranging from 335 to 3500 mg/kg in a rat model with (1 s,4 s)-Eucalyptol bearing the highest values and quercetin holding the lowest one [57, 58].

**Table 10:** The predicted toxicity for 10d, 16, 6d, and 10a using: (a) ProTox-II and (b) Pred-hERG software.

	10d	16	6d	10a
Pro-ToxII				
**Predicted LD50 (mg/kg)**	335	3500	1000	300
**Predicted toxicity class**	3	5	4	3
**Average similarity (%)**	46.52	47.3	33.02	44.28
**Prediction accuracy (%)**	54.26%	54.26%	23	54.26
**Pred-hERG**				
**Prediction/Potency**	Weak or Moderate	Weak or Moderate	strong	Weak or Moderate
**Confidence (%)**	60	60	70	60
**Applicability domain (AD)**	No (Value = 0.24 and limit = 0.26)	No (Value = 0.24 and limit = 0.26)	No (Value = 0.24 and limit = 0.26)	No Value = 0.22 and limit = 0.26

## 4. Conclusion

Herein, the new series of thiazole and formazan linked to 5-Bromo-indan and their structures were assured based on all possible analytical techniques. Five derivatives were precipitated on a nanosized scale. The anticancer activity of the tested derivatives indicated that one derivative, **10d** showed activity more than the two reference drugs 49used in the case of SNU-16, while the IC50 of five derivatives was better than the two benchmarks used in the case of COLO205. All potent derivatives showed a strong fit with the active site of the two tested proteins (gastric cancer (PDB = **2BID**) and colon cancer (PDB = **2A4L**)) in the molecular docking study. The Pharmacophore and ADME studies of the new derivatives showed that most derivatives revealed promising bioactivity, which indicates the drug-likeness properties against kinase inhibitors, protease, and enzyme inhibitors. In addition, the ProTox-II showed that the four compounds **10d**, **16**, **6d**, and **10a** are predicted to have oral LD_50_ values ranging from 335 to 3500 mg/kg in a rat model with (1 s,4 s)-Eucalyptol bearing the highest values and quercetin holding the lowest one. Pred-hERG model played an important rule as SAR tool with predication of the most common like drugs like the new series synthesized with the interpretation of Probability of toxicity for compounds.

## Supporting information

S1 File All spectral charts and the pictures of instruments were listed in the suplementry file. (DOCX)
