# Supplementary material for: Indenyl-thiazole and indenyl-formazan derivatives: Synthesis, anticancer screening studies, molecular-docking, and pharmacokinetic/ molin-spiration properties
Source: PLoS One. 2023 Mar 1;18(3):e0274459. doi: 10.1371/journal.pone.0274459 (PMC9977057; doi:10.1371/journal.pone.0274459)

**Indenyl-thiazole and indenyl-formazan derivatives: Synthesis, Anticancer screening studies, Molecular-docking, and pharmacokinetic/ Molin-spiration properties**

**Ghaidaa H. Alfaifi^a^, Thoraya A. Farghaly^a*^ and** [**Magda H. Abdellattif**](https://www.researchgate.net/profile/Magda_Abdellattif?_sg%5B0%5D=NAhZxRf8YRslLR99QakoIQhjQ6e7dueC770c7OKbsRX2nTZrxr1T5KrT-9ybcLiUXVoecWk.24vjok_eOouNhBqq_kqhFxGcCvZHbNTaSaiBesI5jQrAyiB3hN5LzLo7RsxCClT3gO3rvrNP4b5DSwExeKxc0Q&_sg%5B1%5D=bg5GfSHzGM1n9nVJLCxiElrjCPj5kt0g-nN7TTA0y98rVJprPI4c2aotBGLkxB7RGO4OpSo.ITzVYjSfMOvyfehxRVKW3yyDAACQ8m4s-oXNsBaEJAwP49J8uHVOYX-BAlHvDmJEVU1JEIcSFk4p4aSHHUPrOQ)**^b^**

^a)^ *Chemistry Department, Faculty of Applied Sciences, Umm Al-Qura University, Makkah, Saudi Arabia*

^b)^ *Department of Chemistry, College of Science, Taif University, P. O. Box 11099, Taif 21944, Saudi Arabia*

*Corresponding author e-mail:* [*thoraya-f@hotmail.com*](mailto:thoraya-f@hotmail.com)*; tamohamed@uqu.edu.sa*


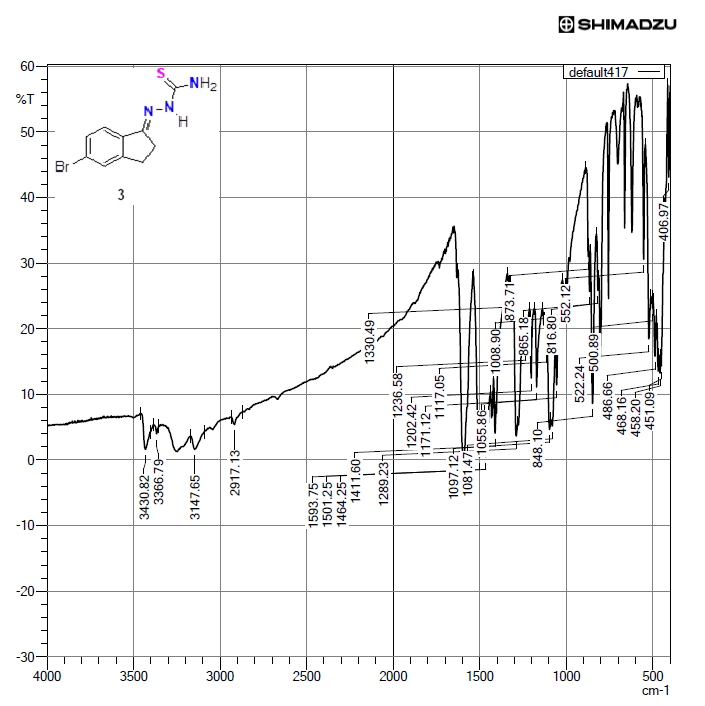


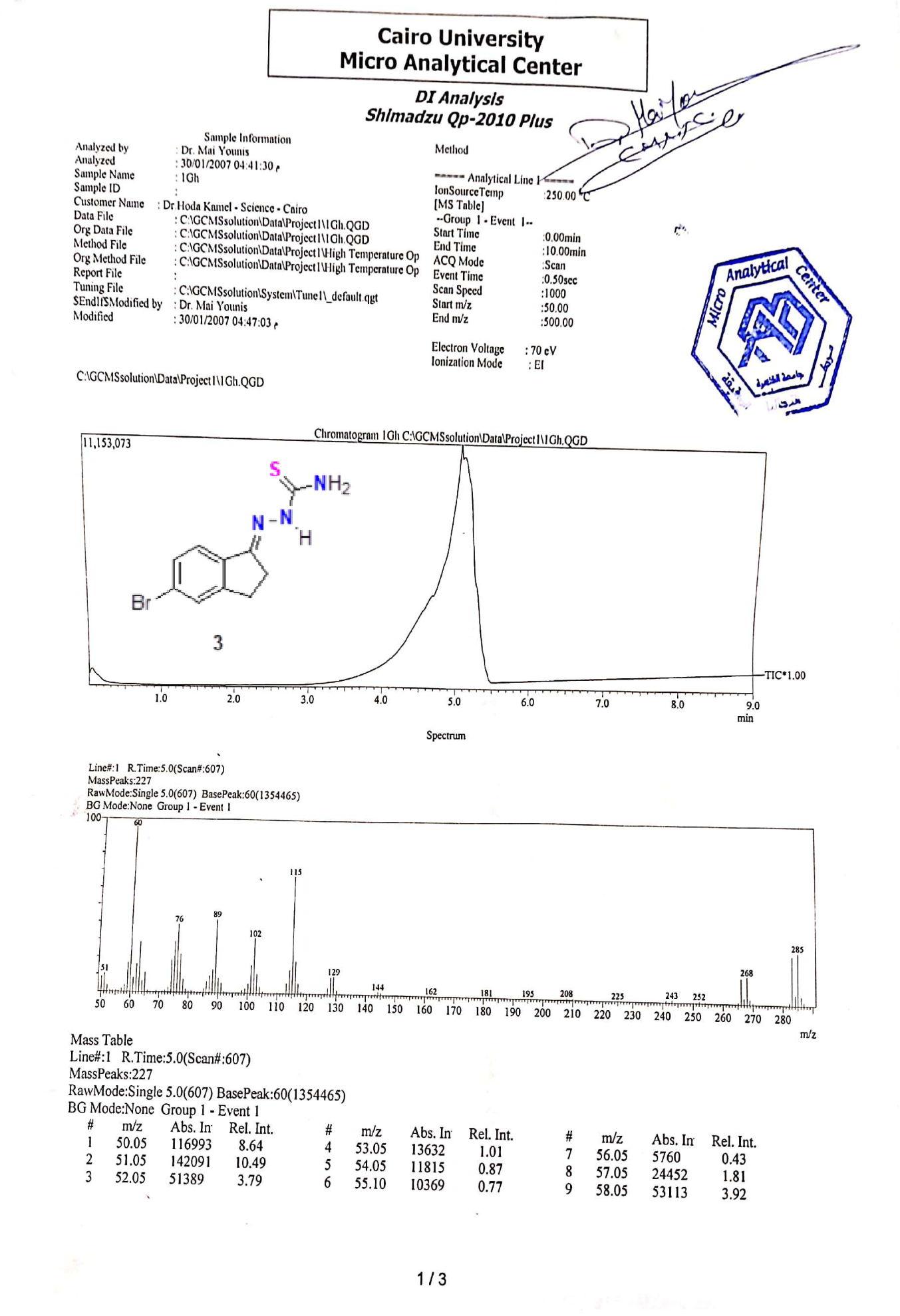


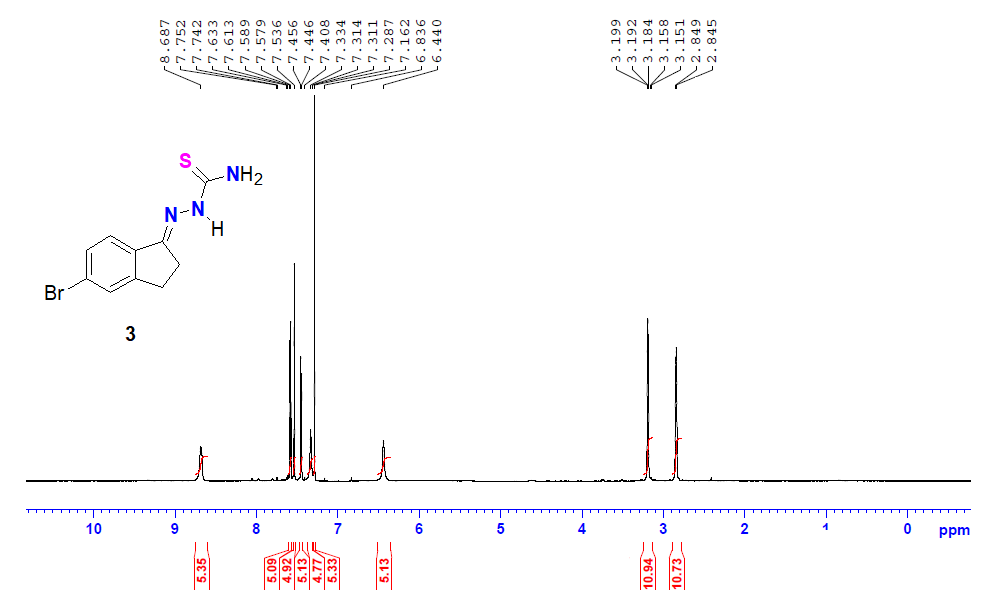


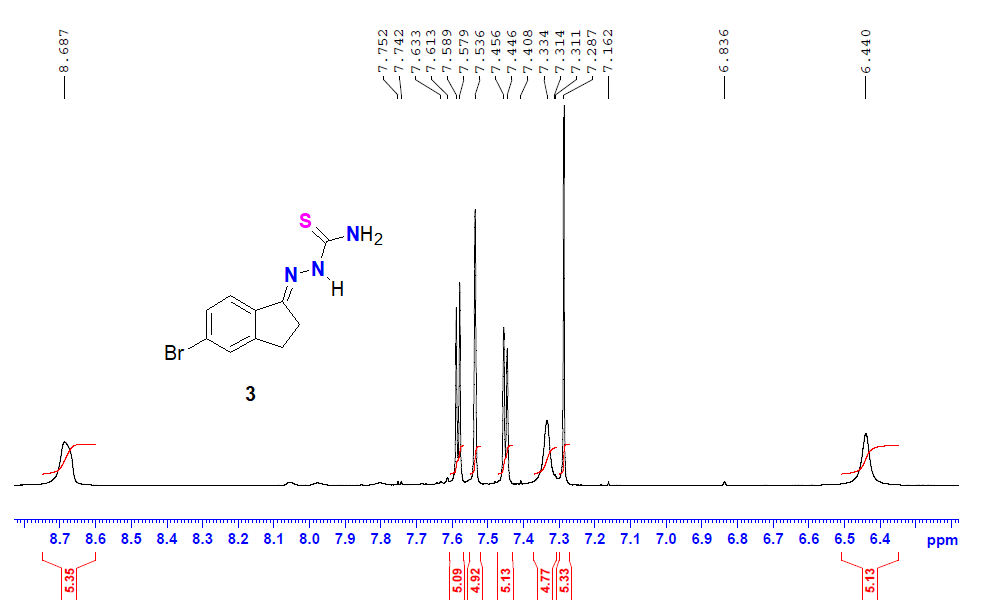


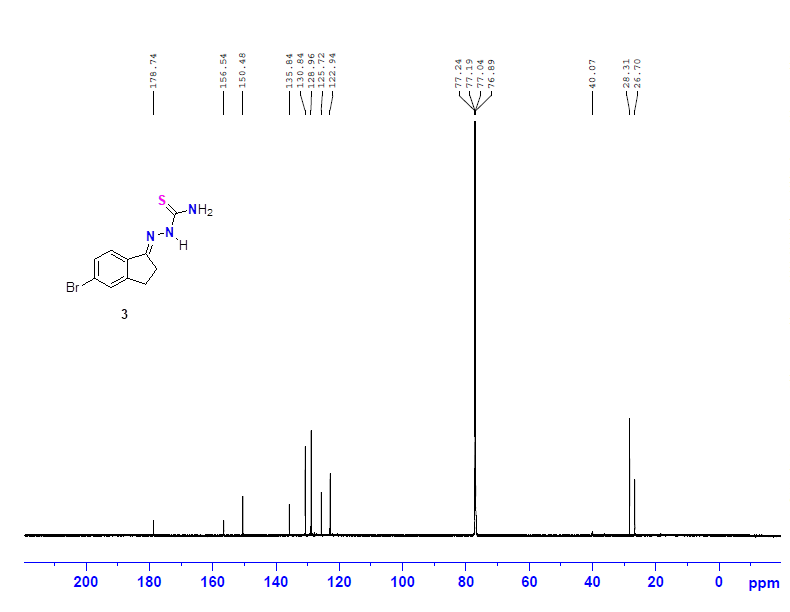

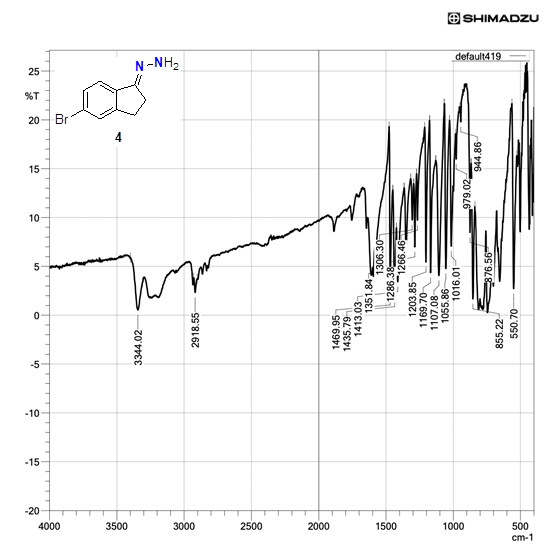


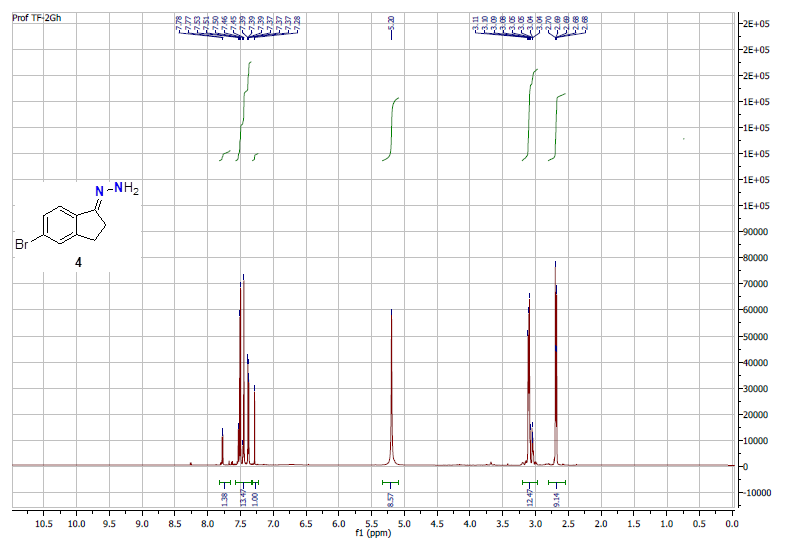


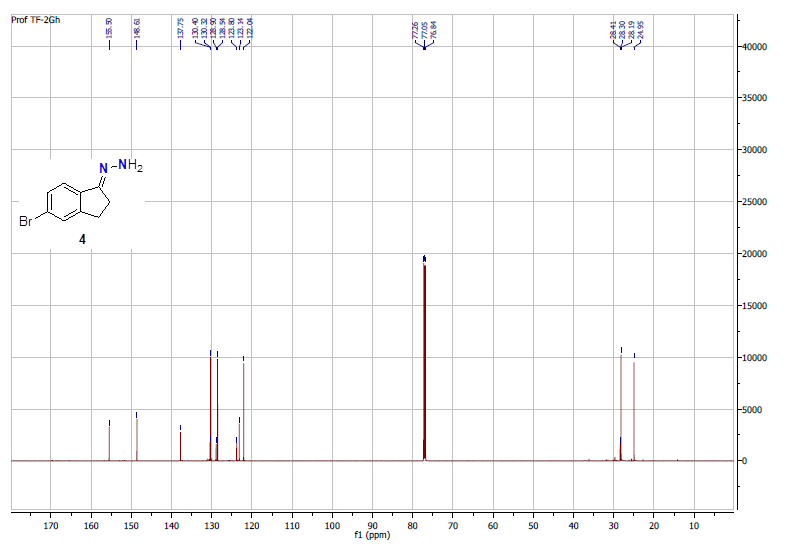


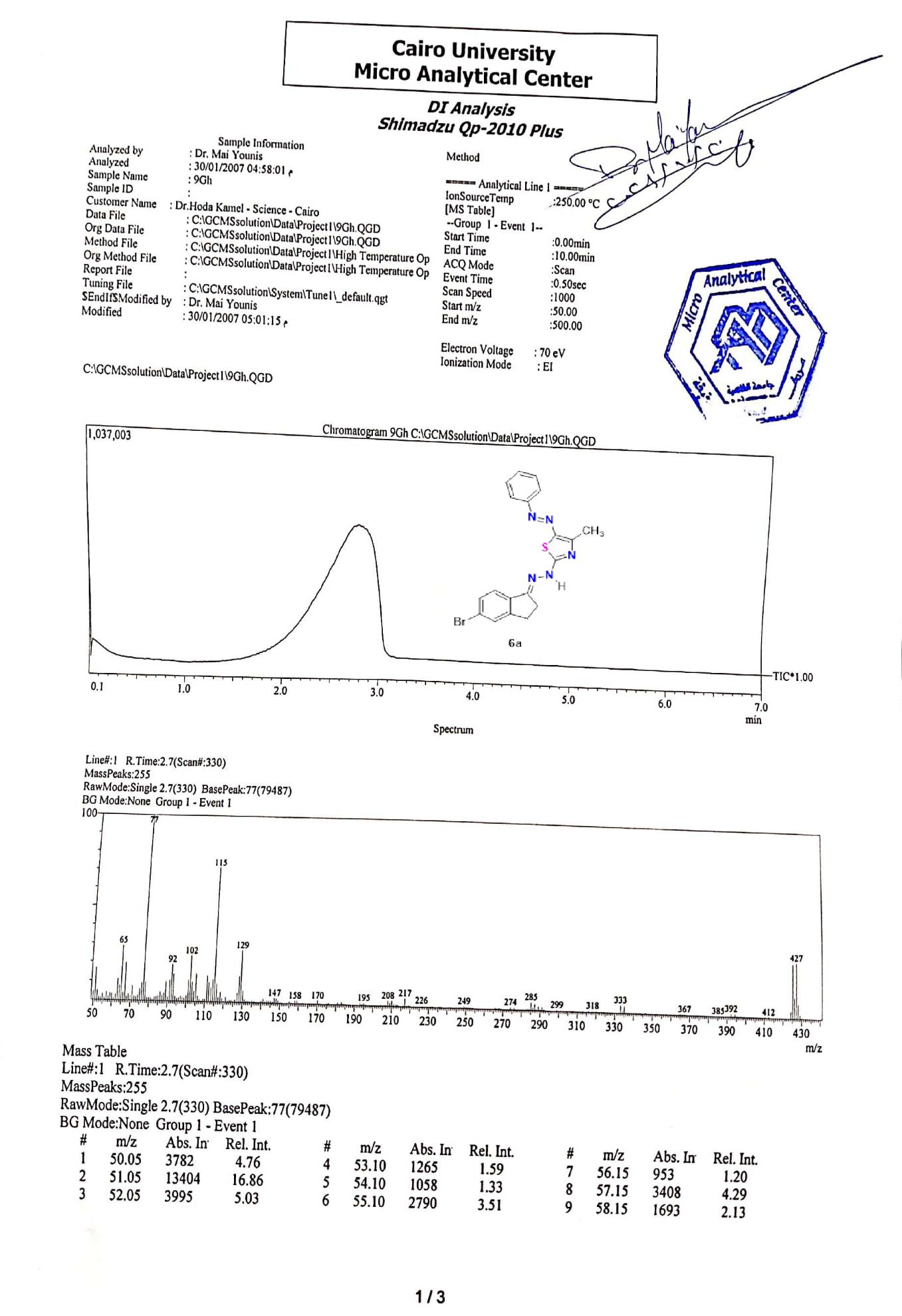


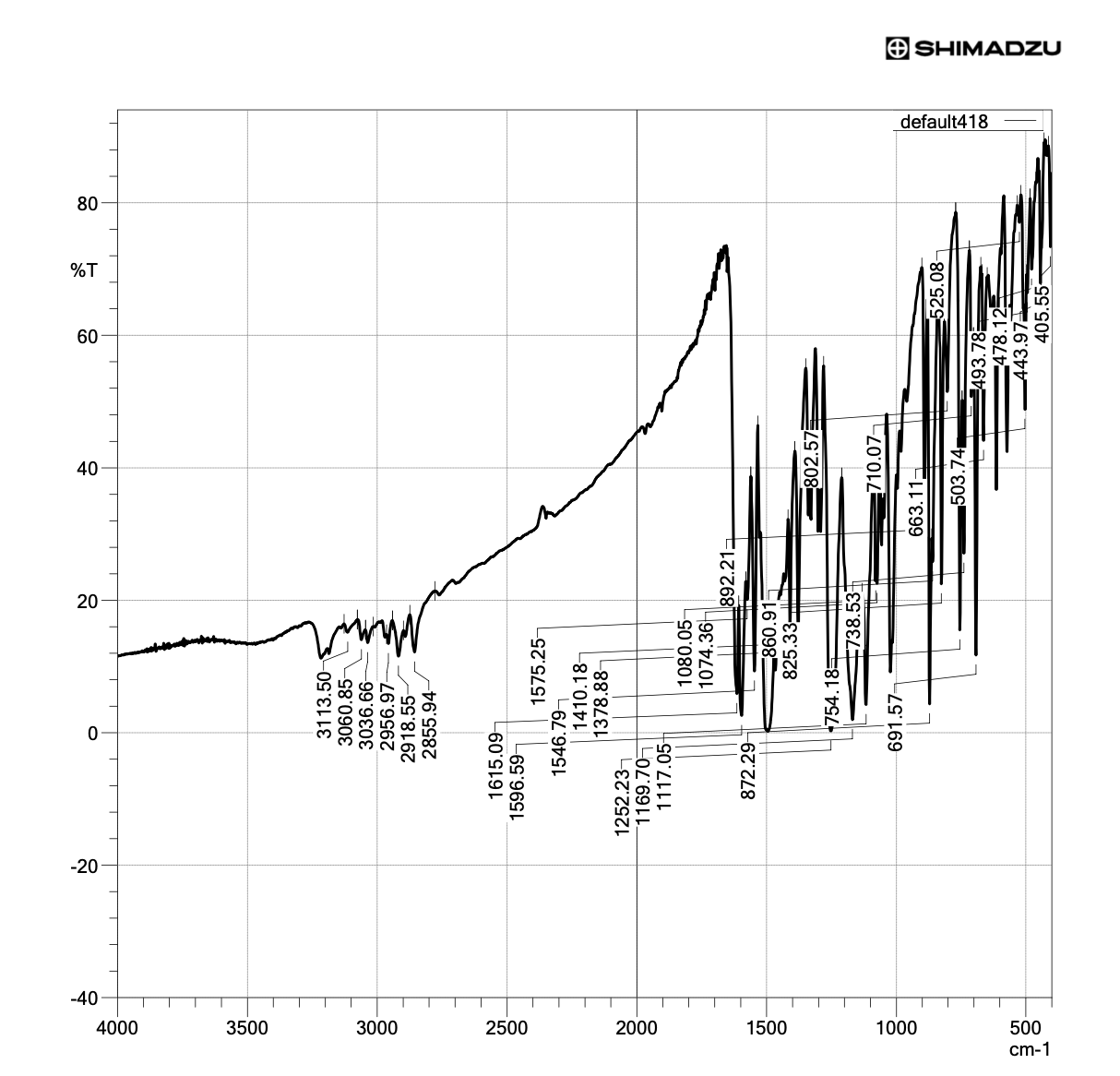

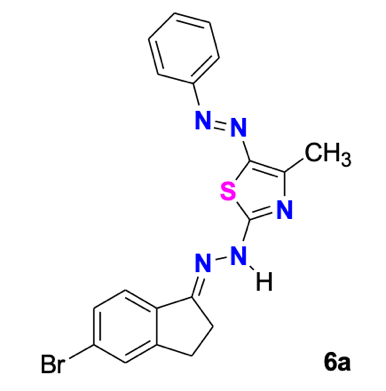


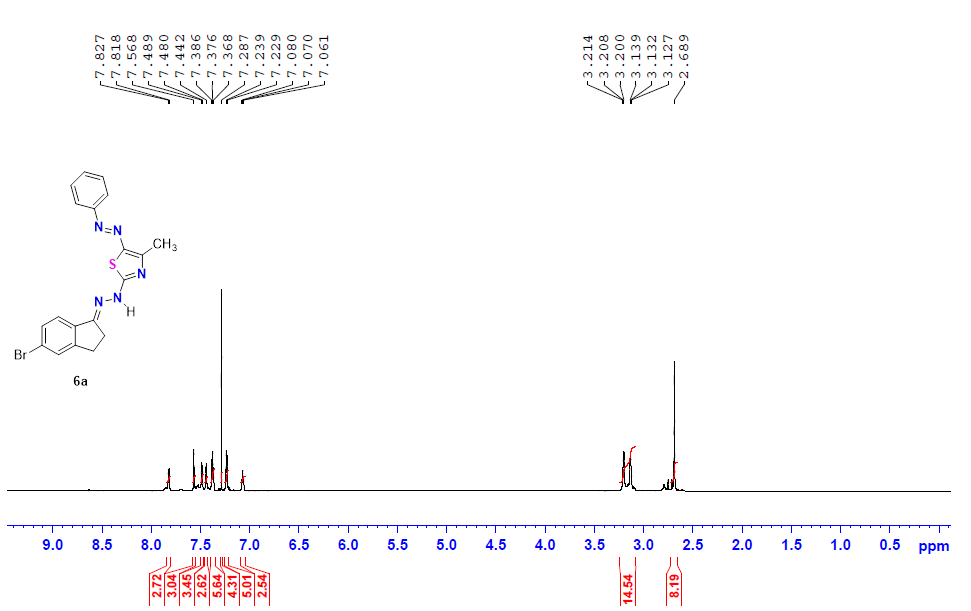


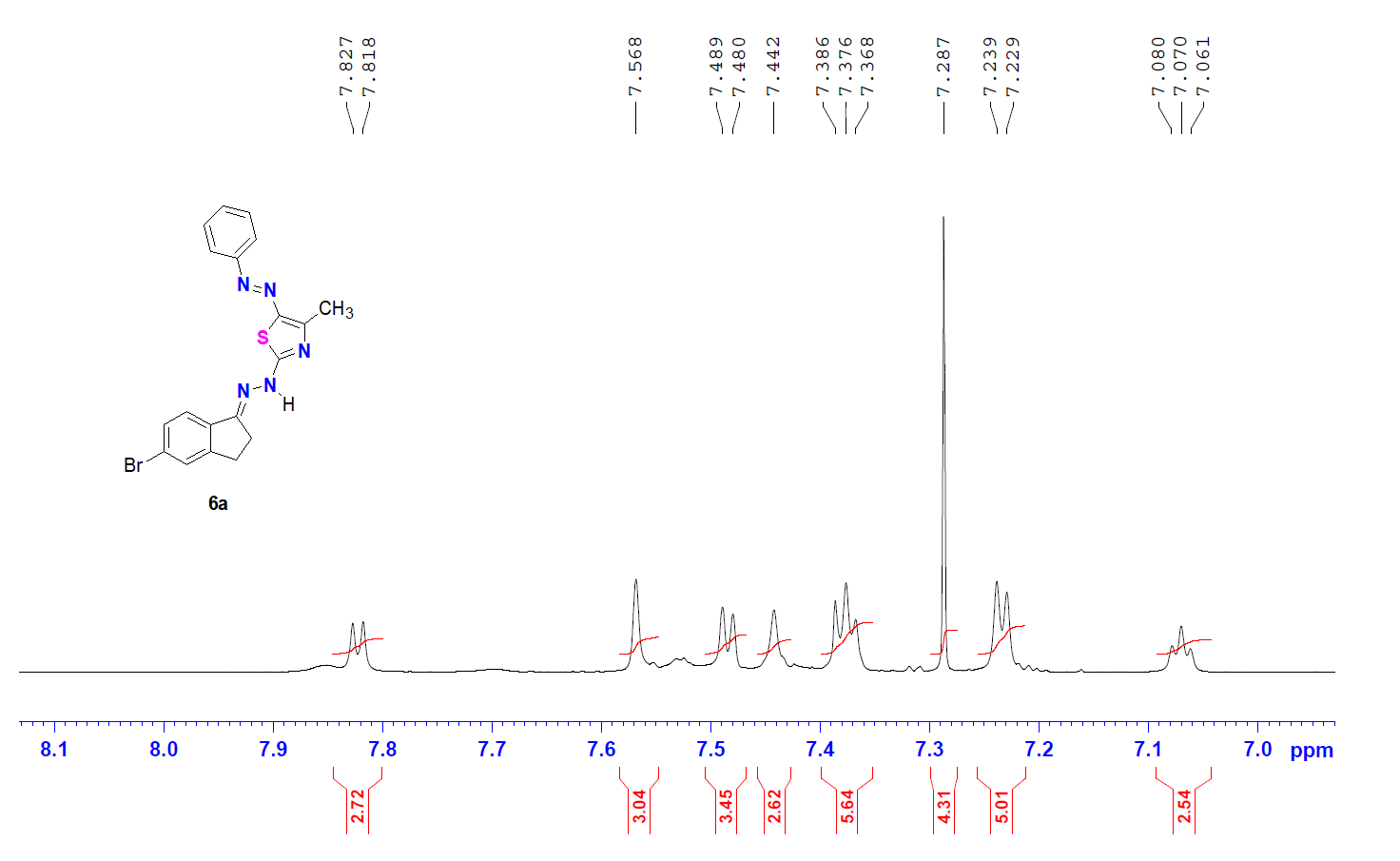


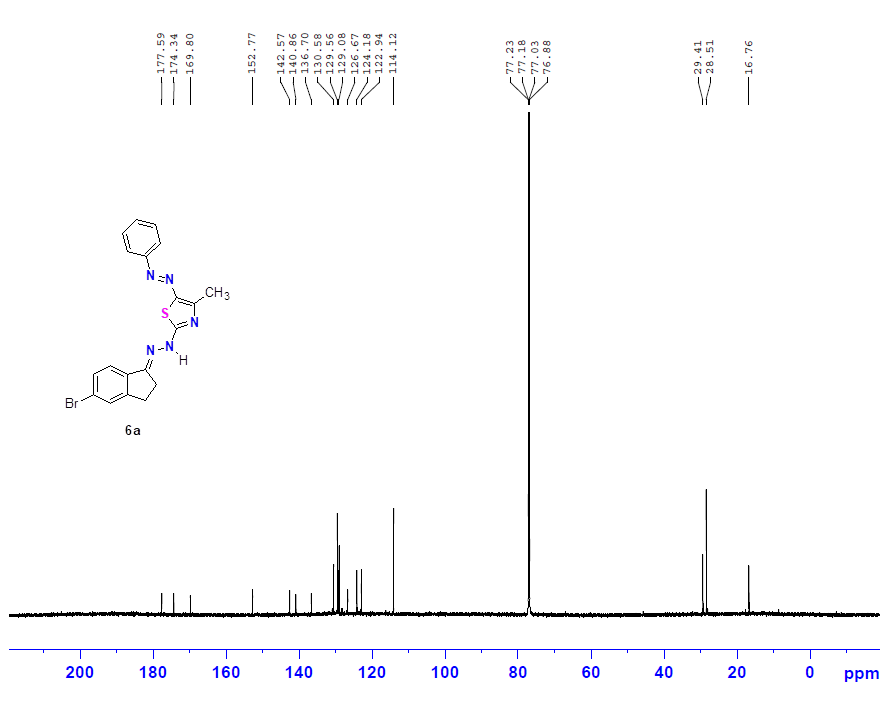


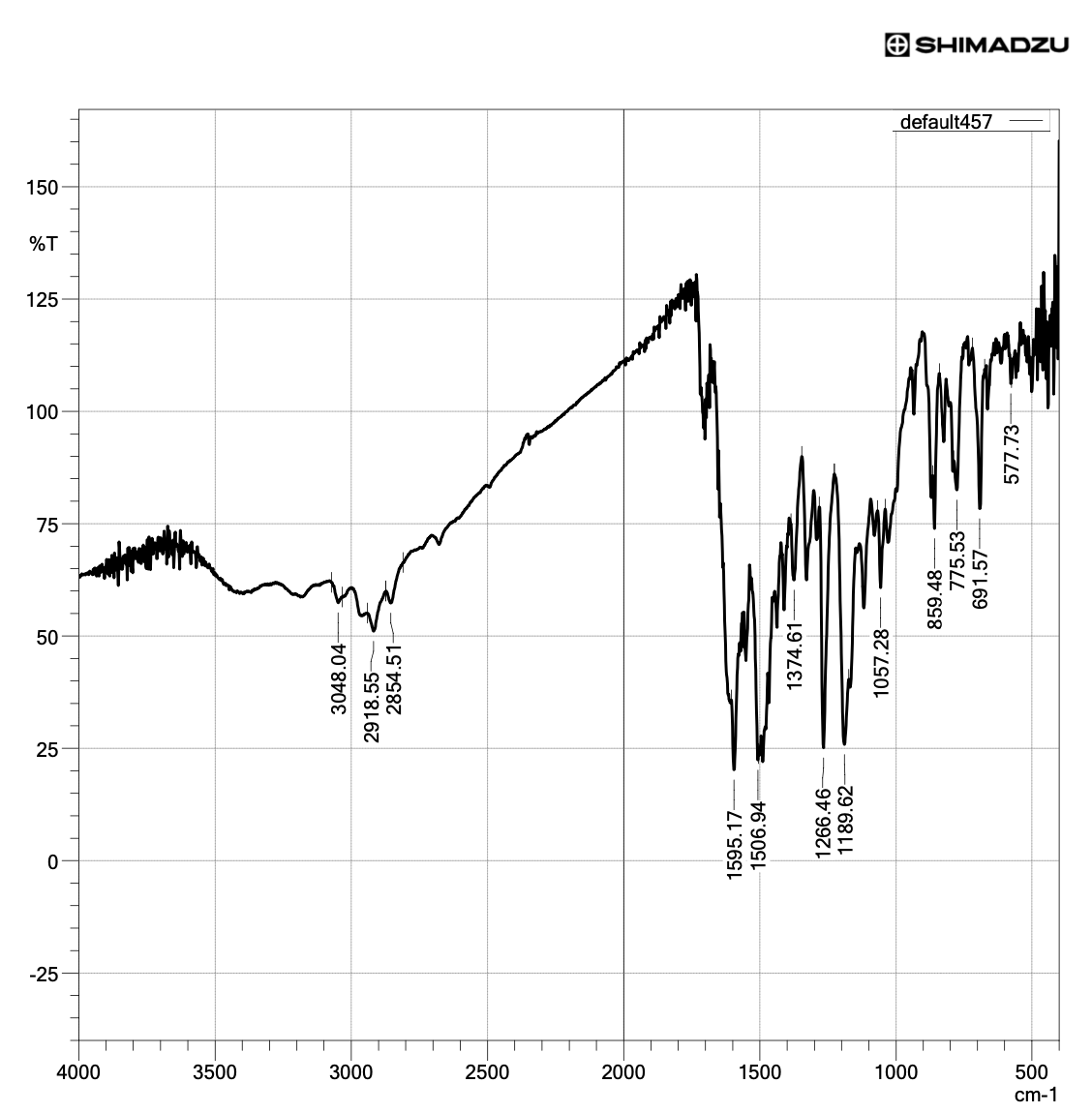

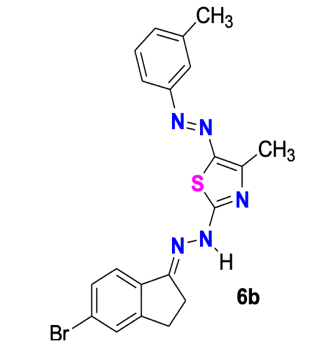


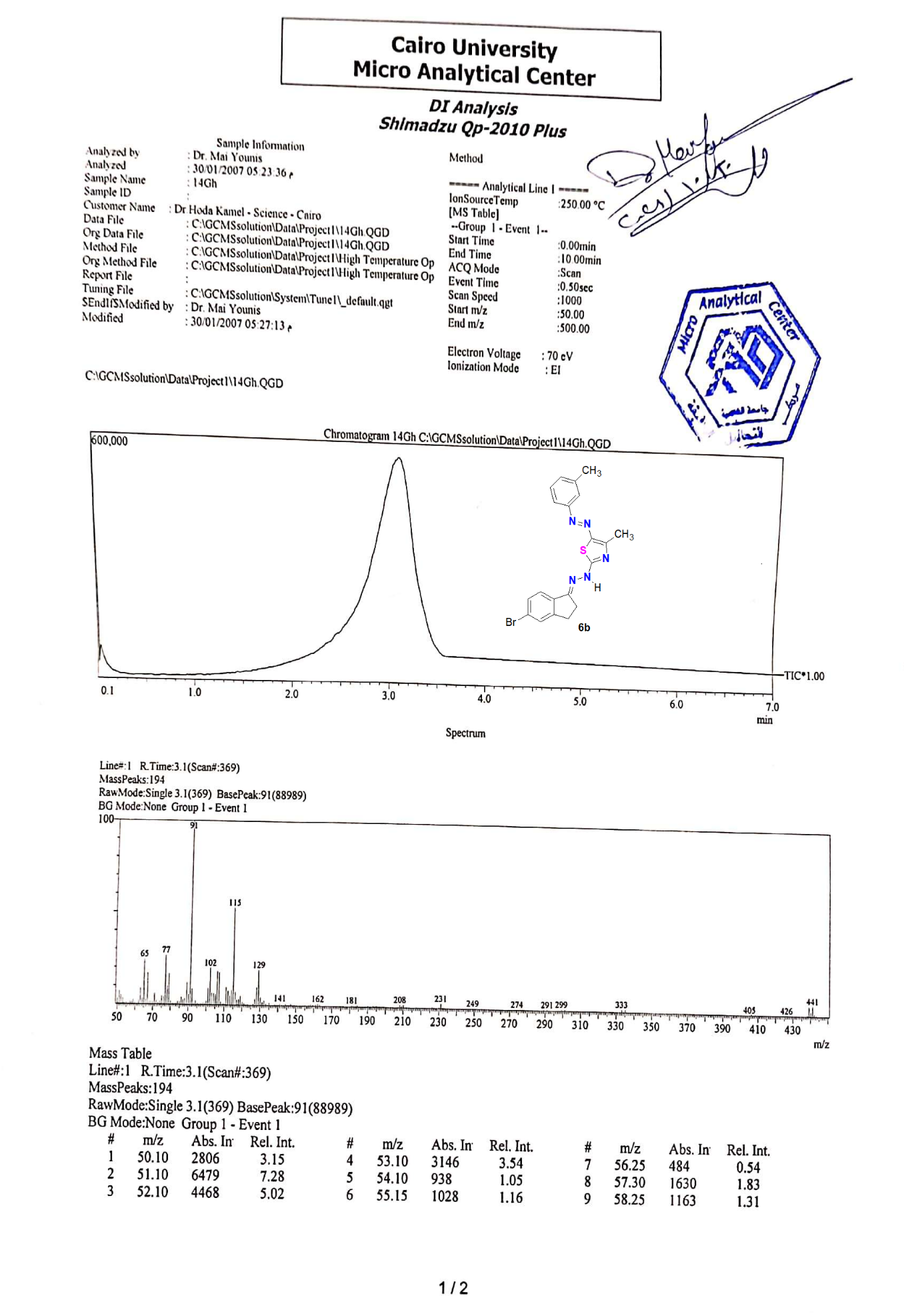


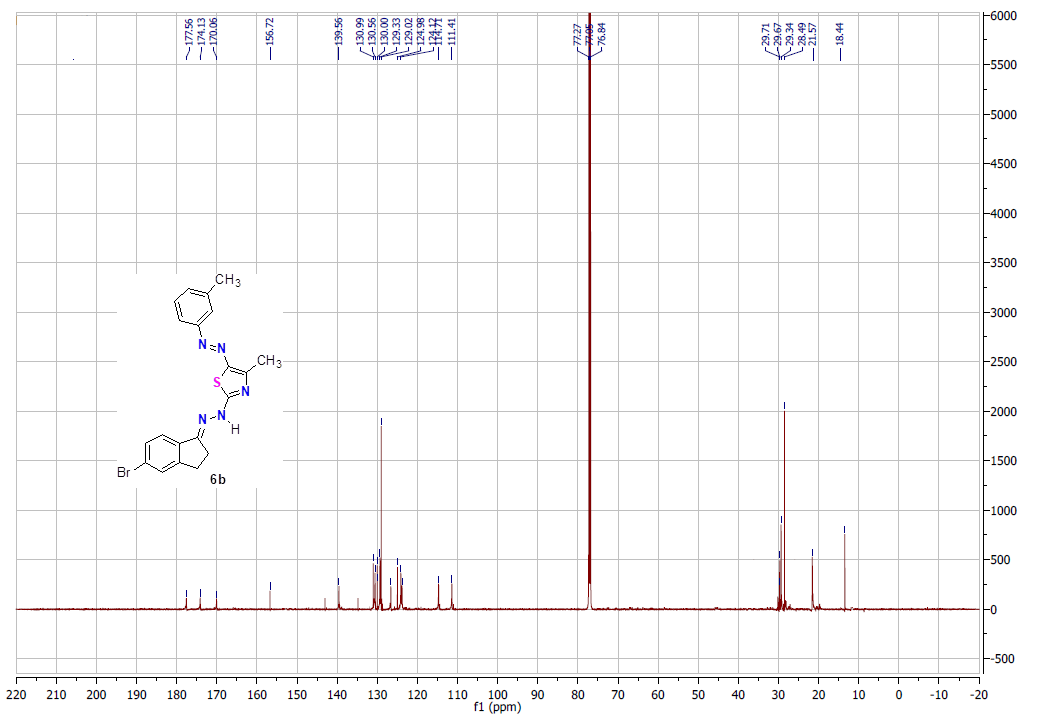


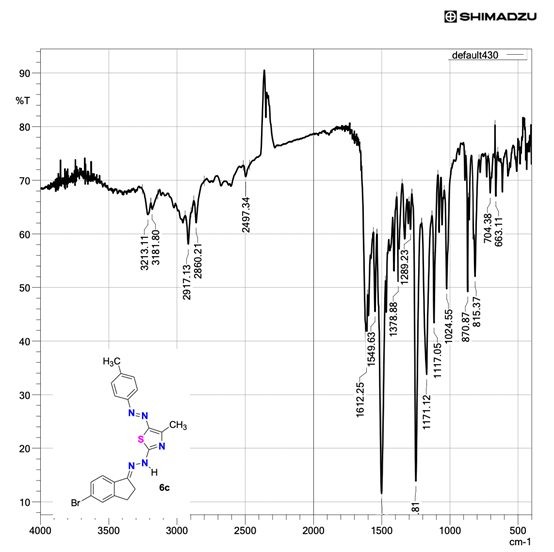


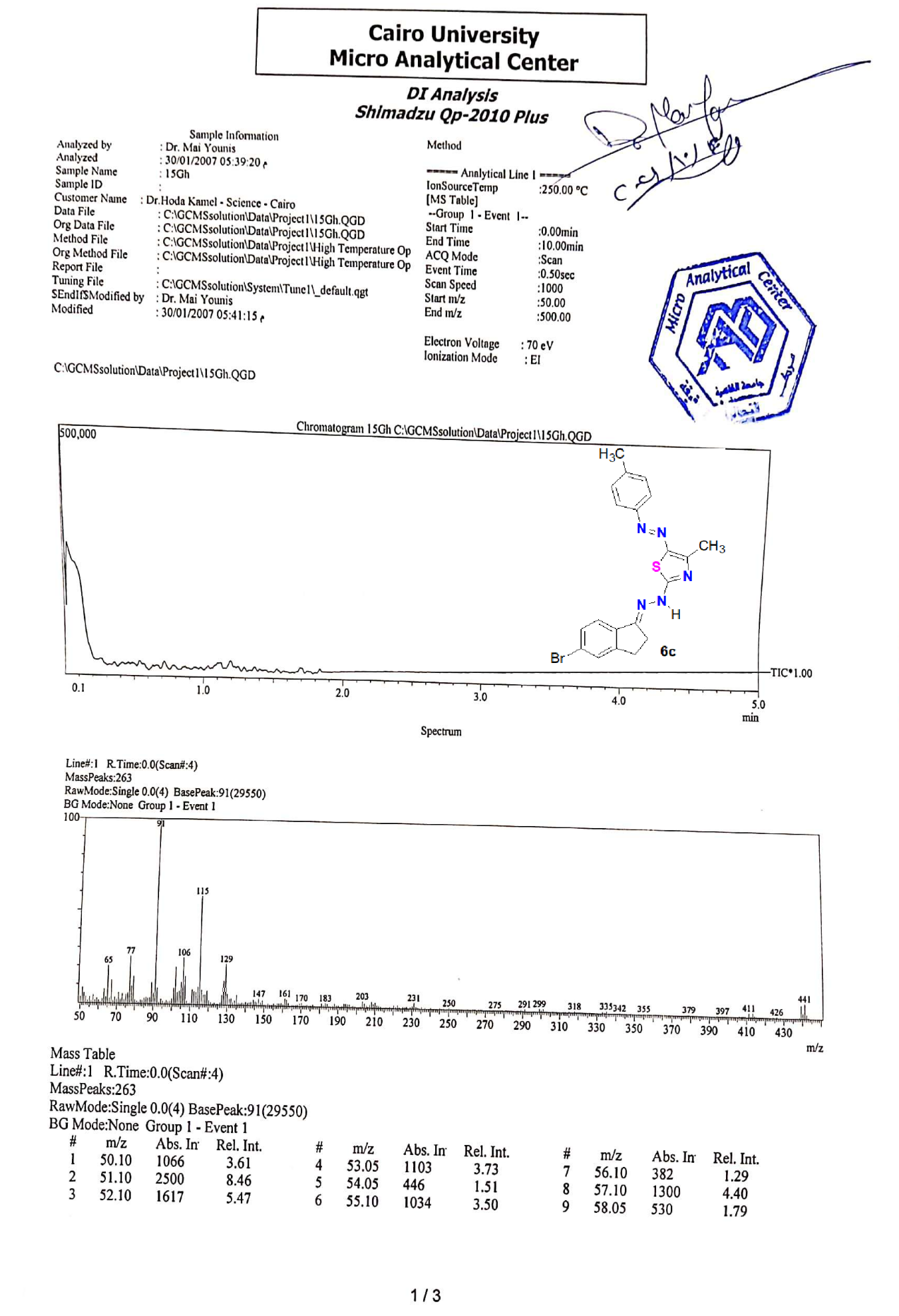


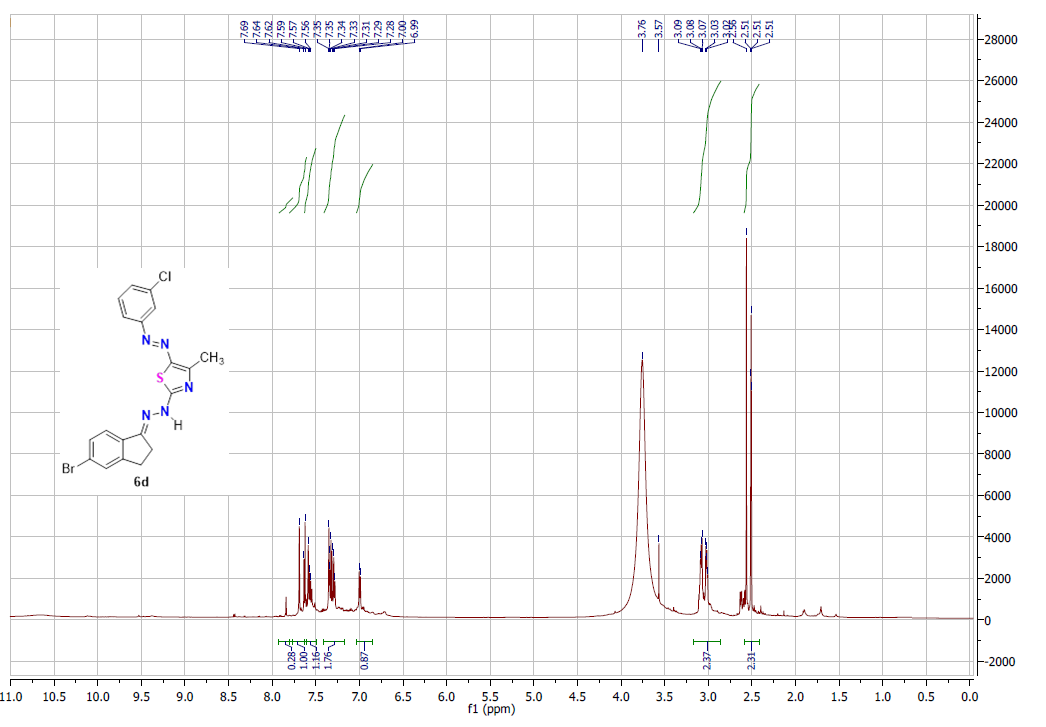

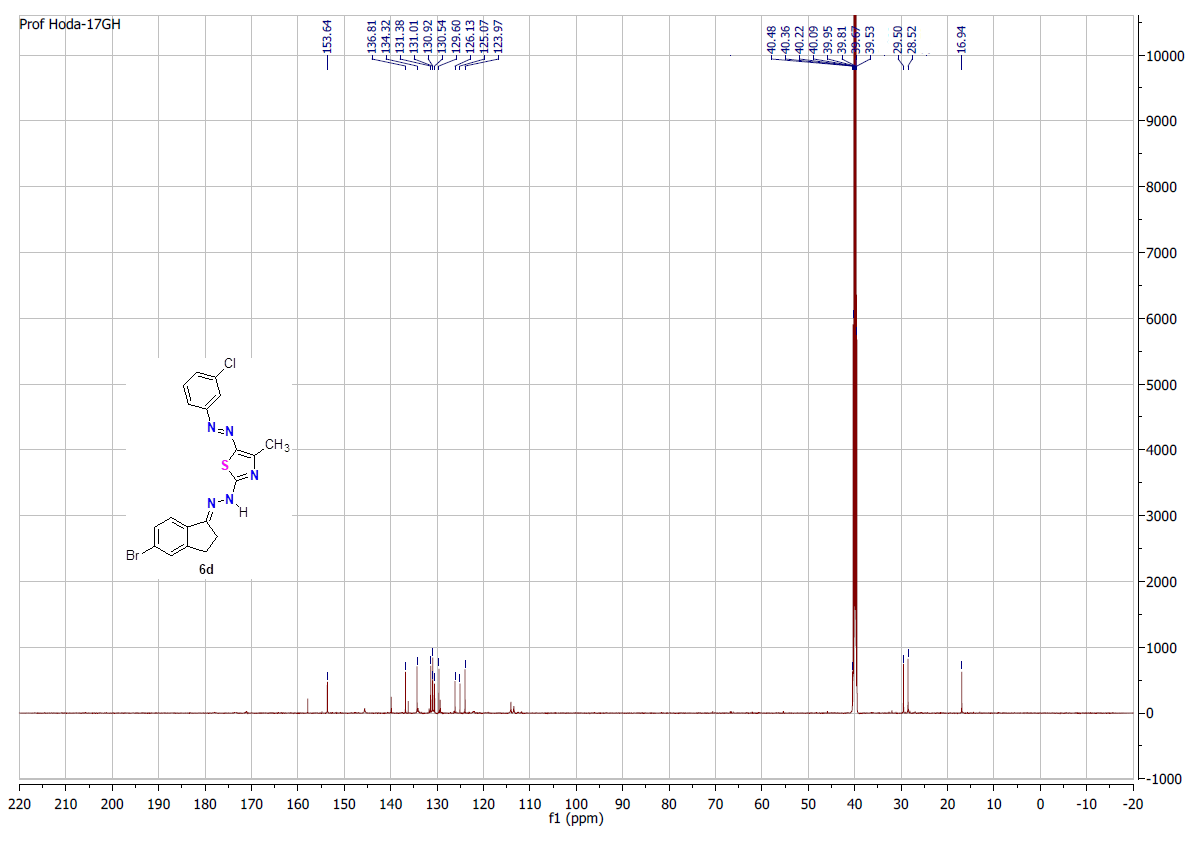


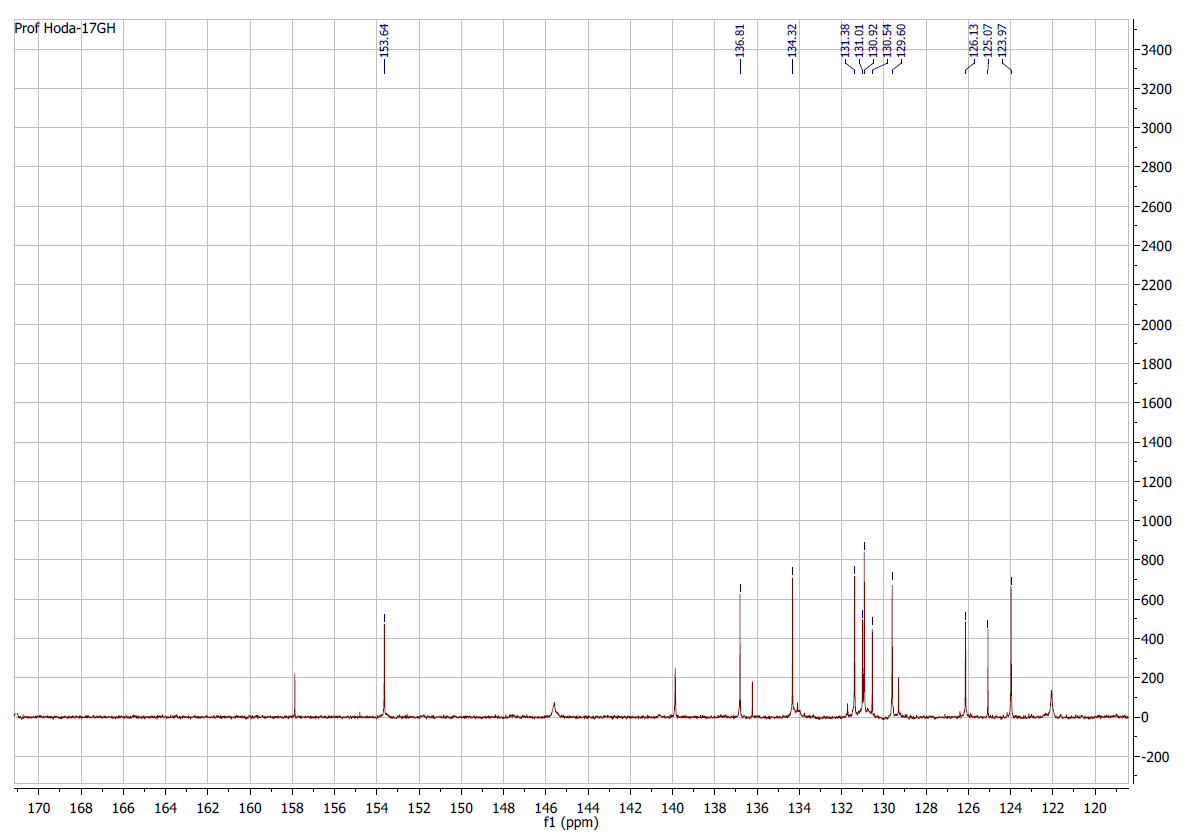


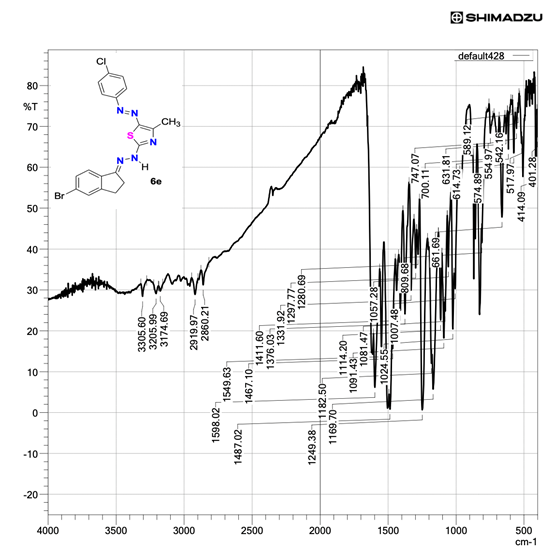


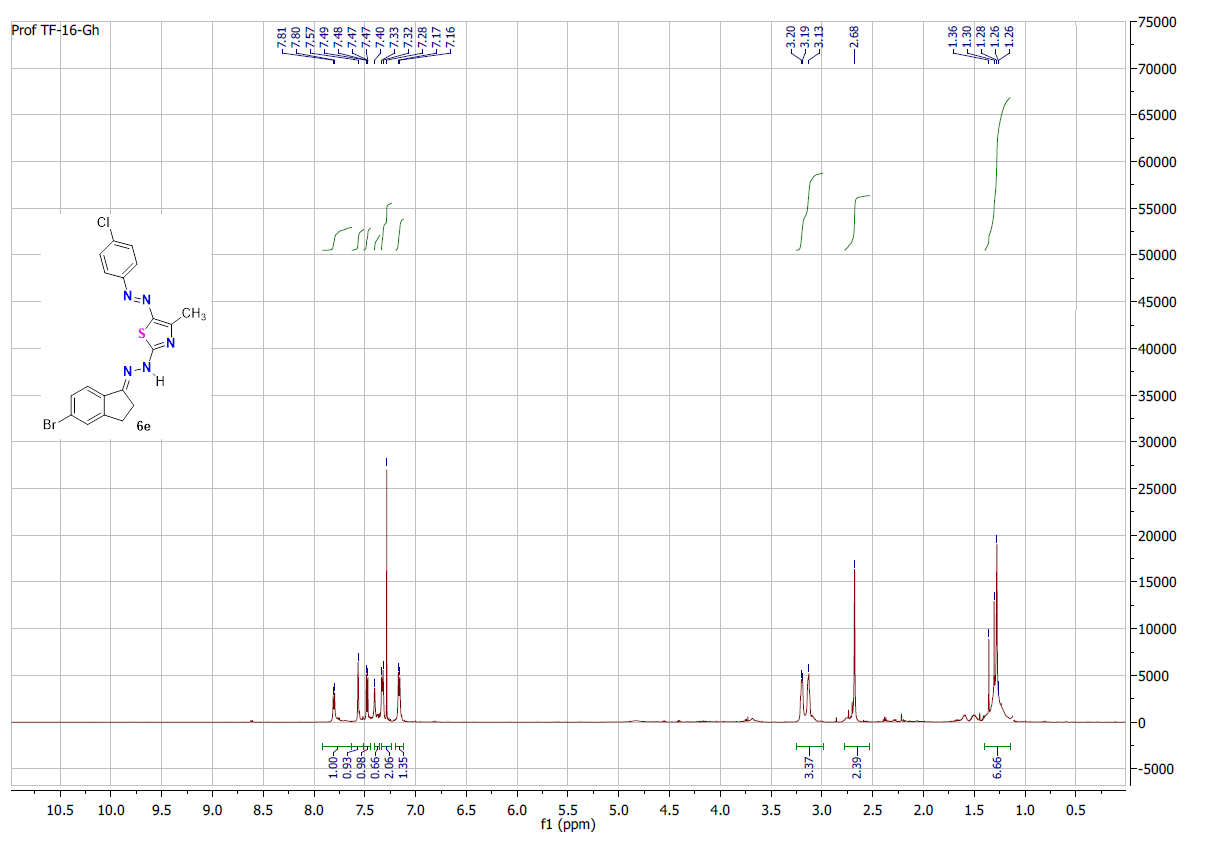


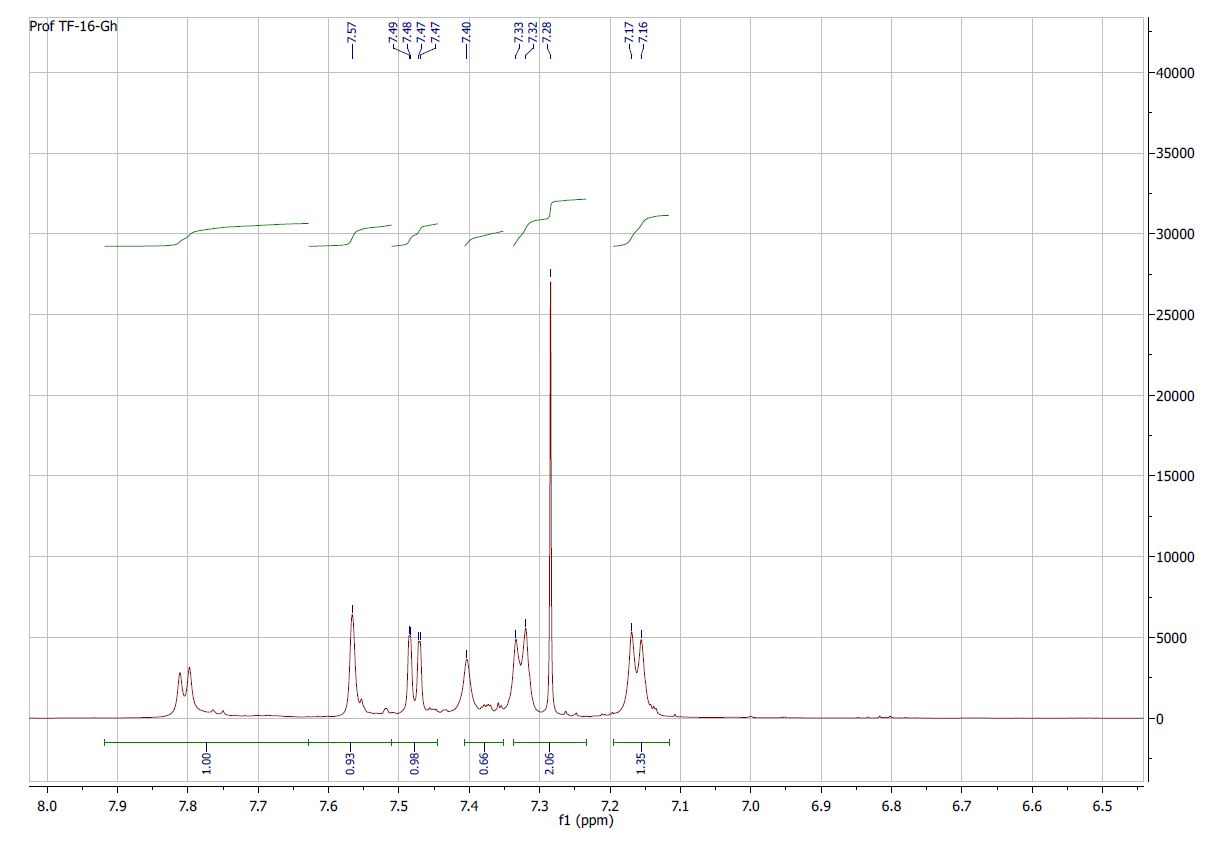


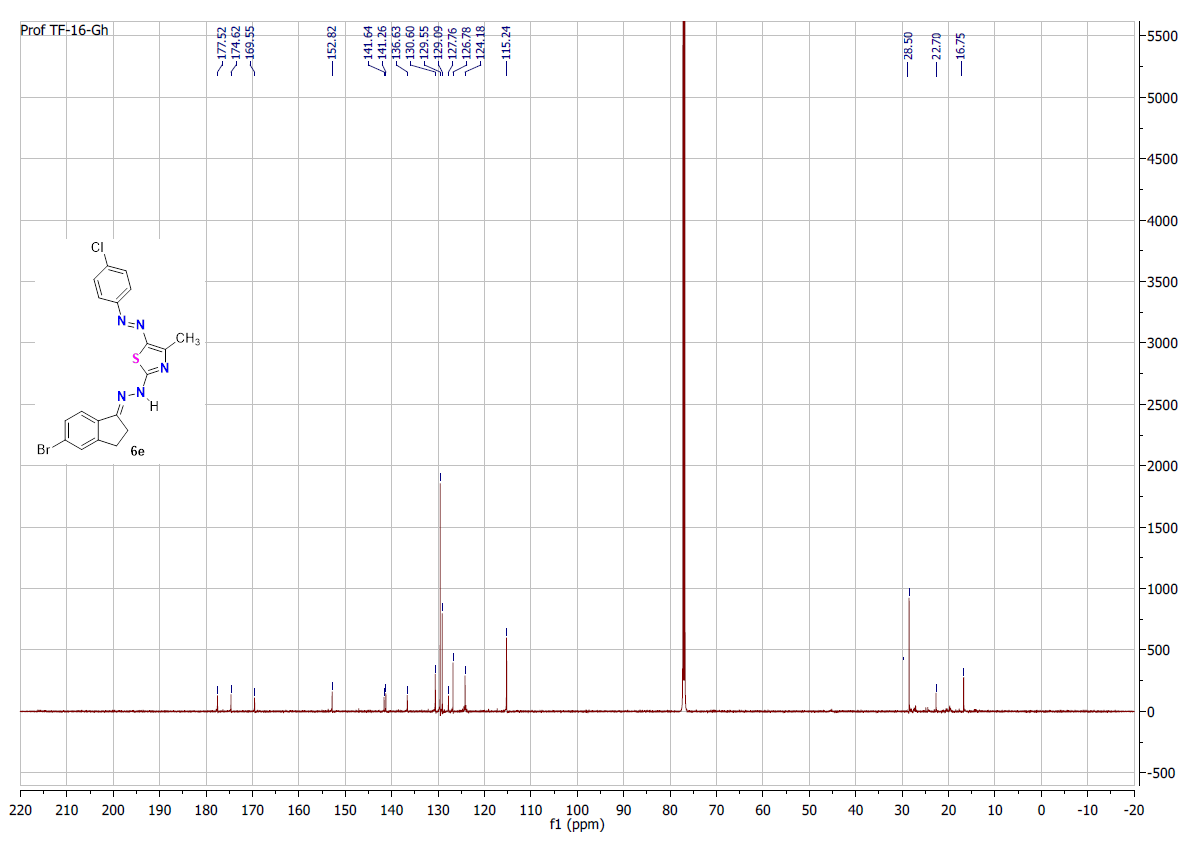


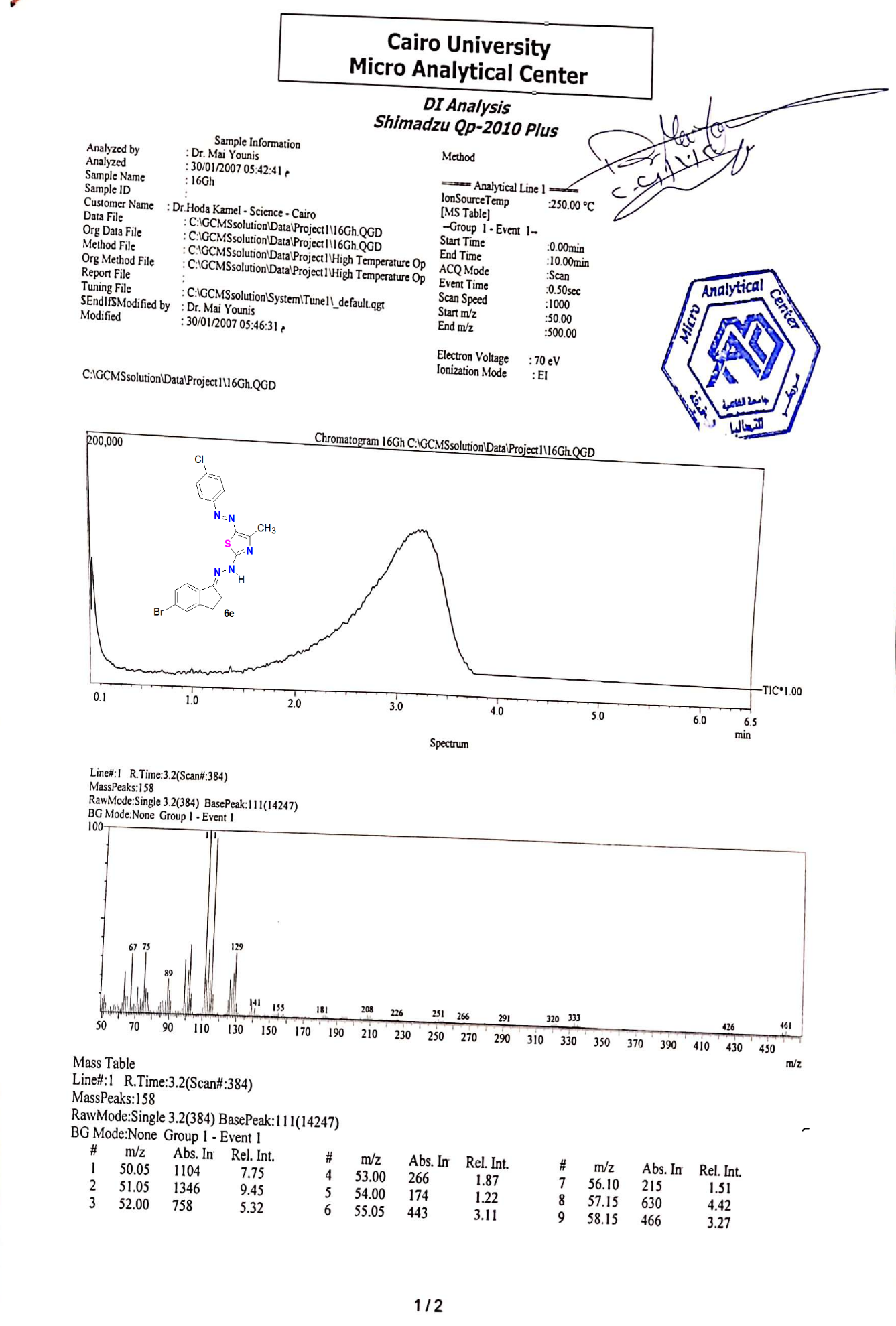


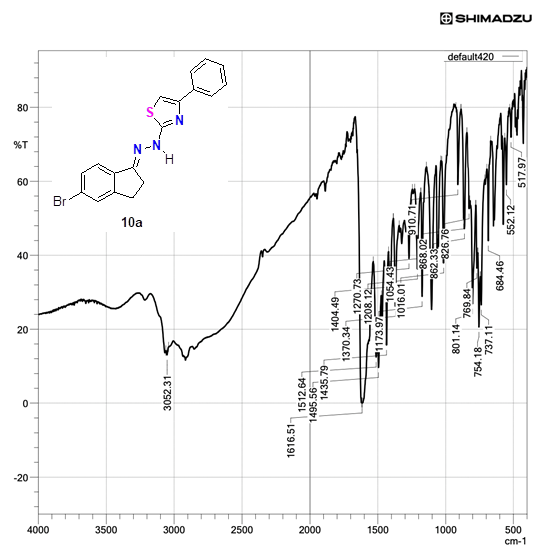


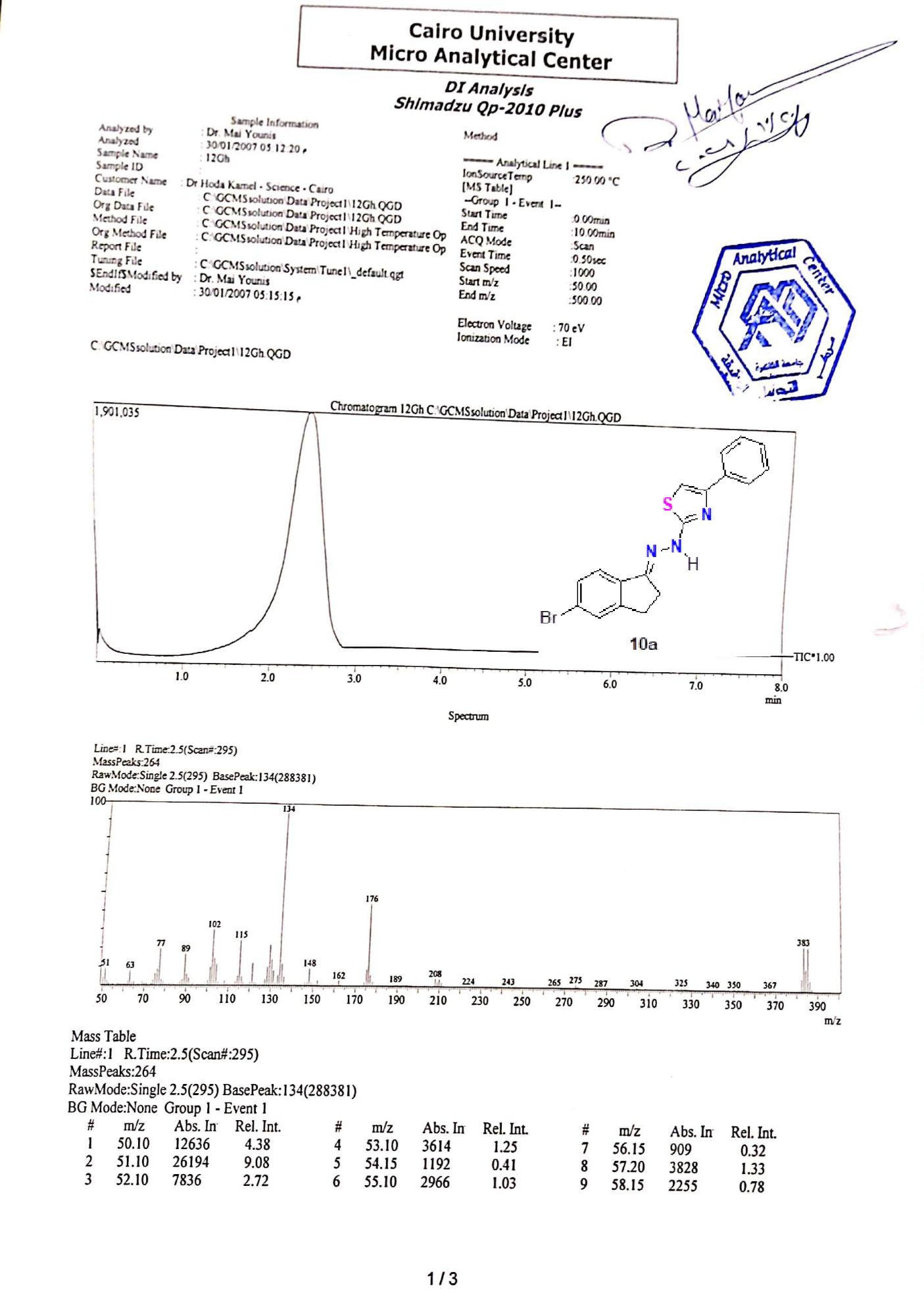


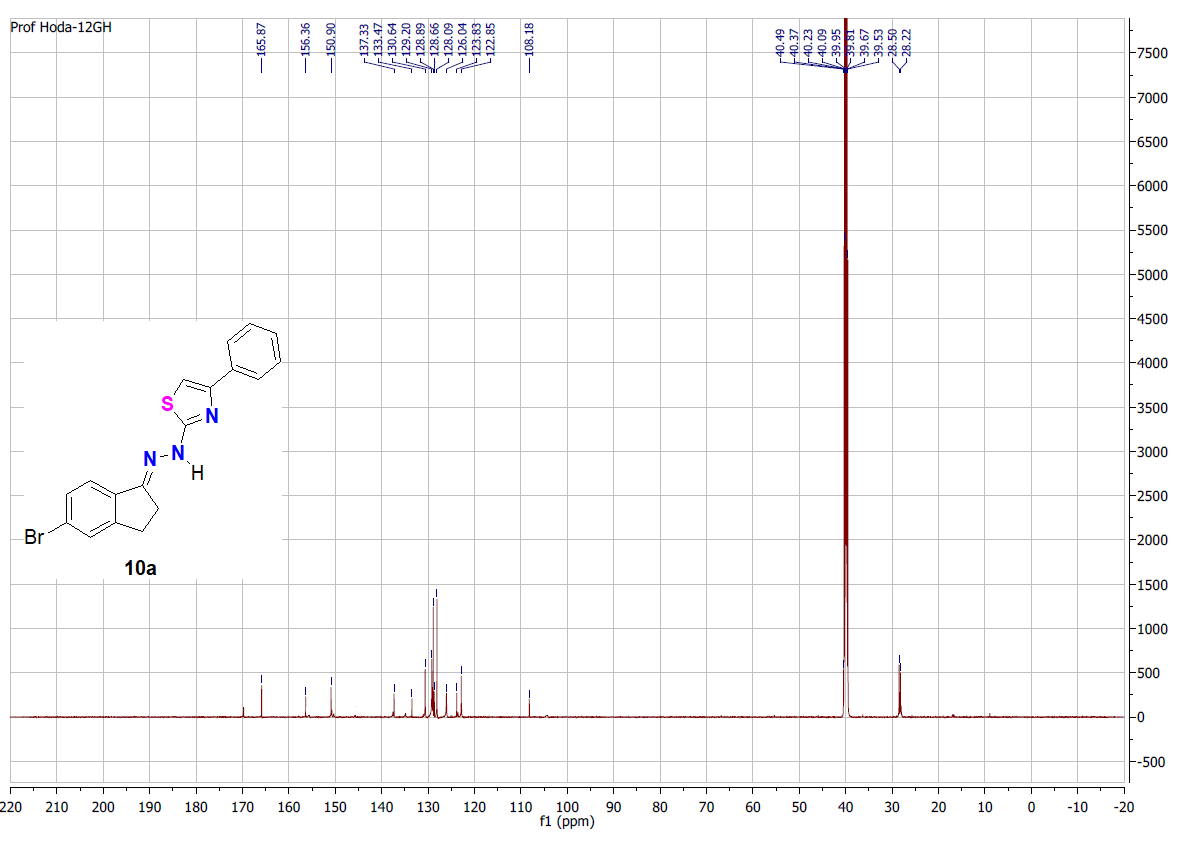

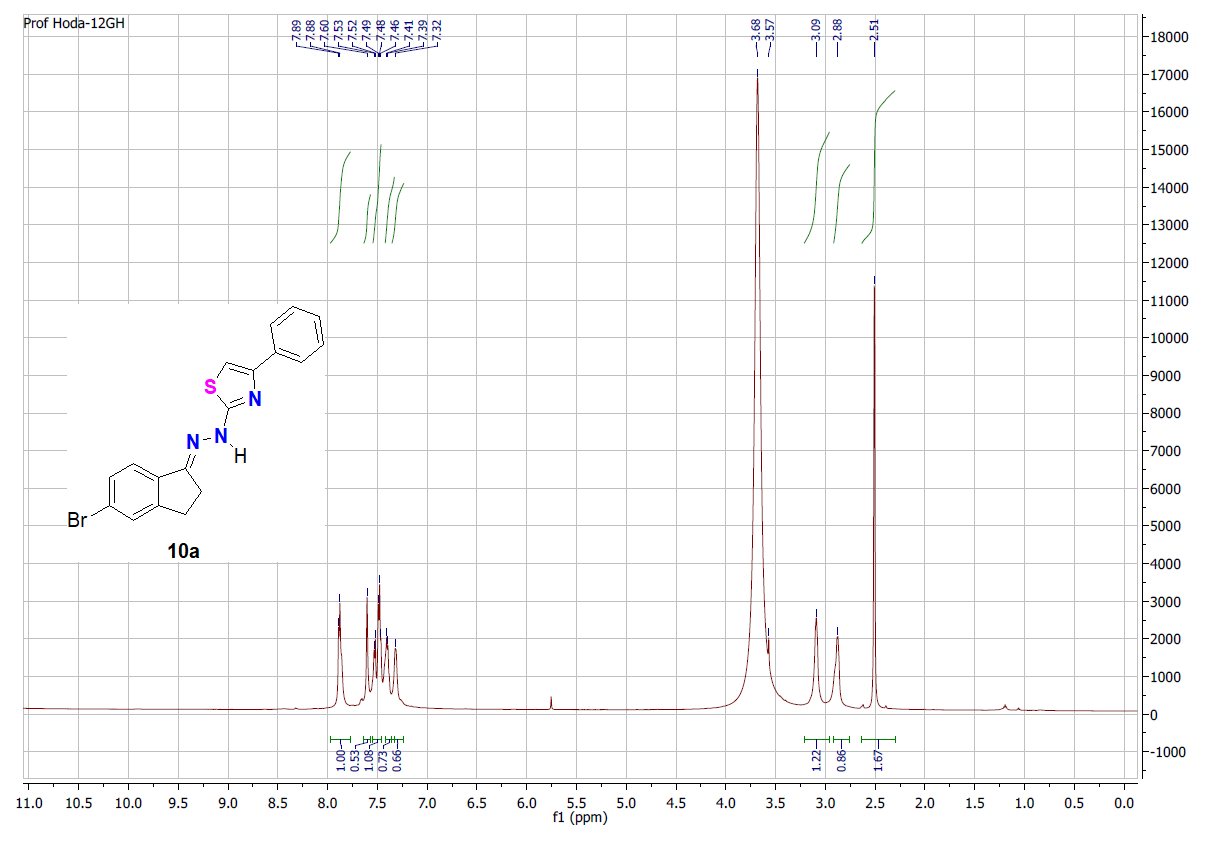


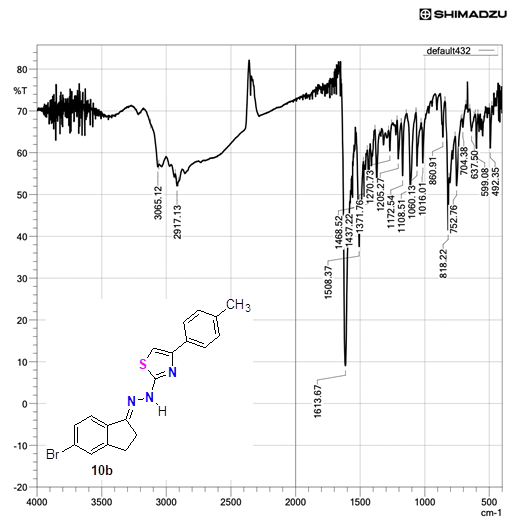


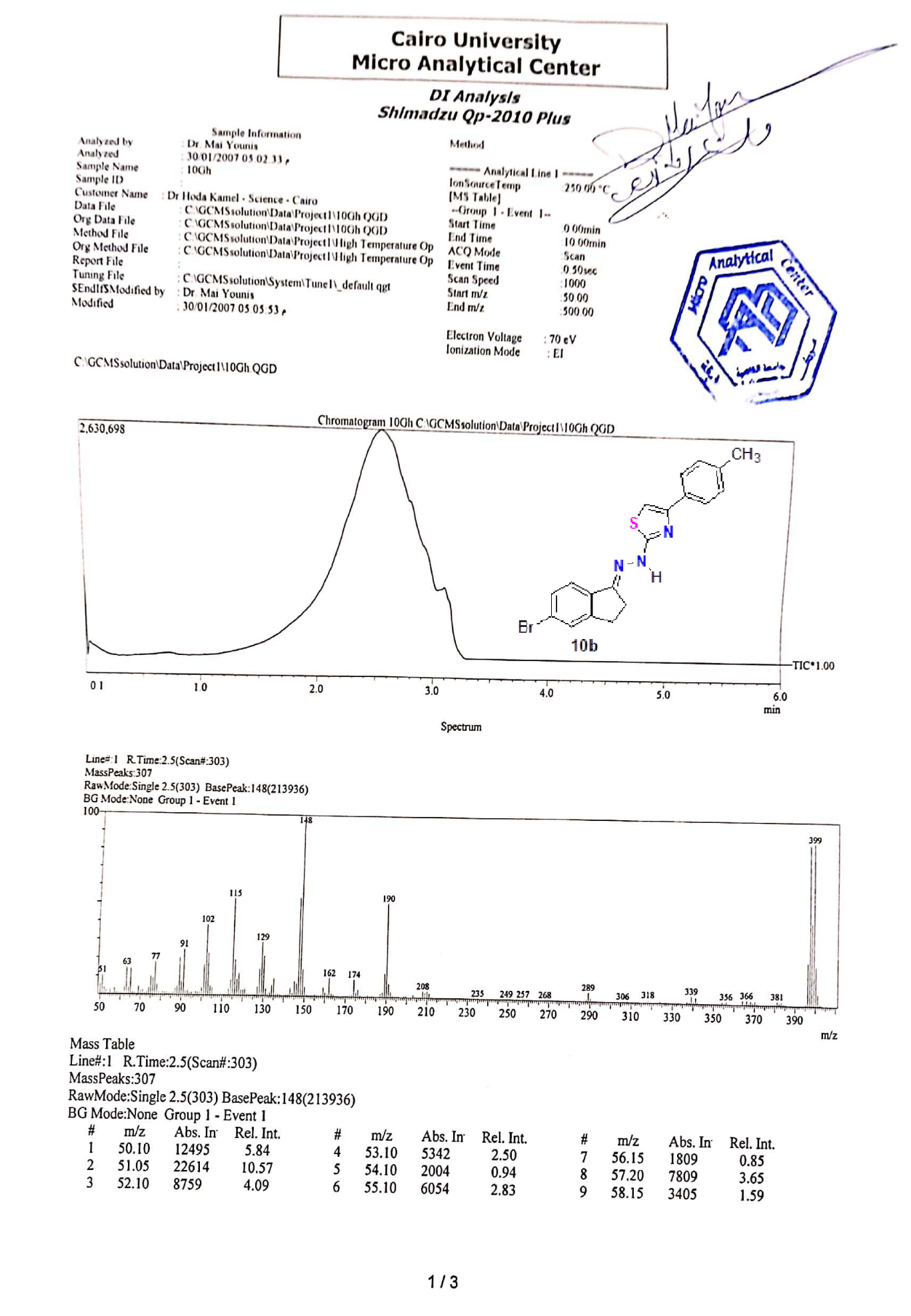


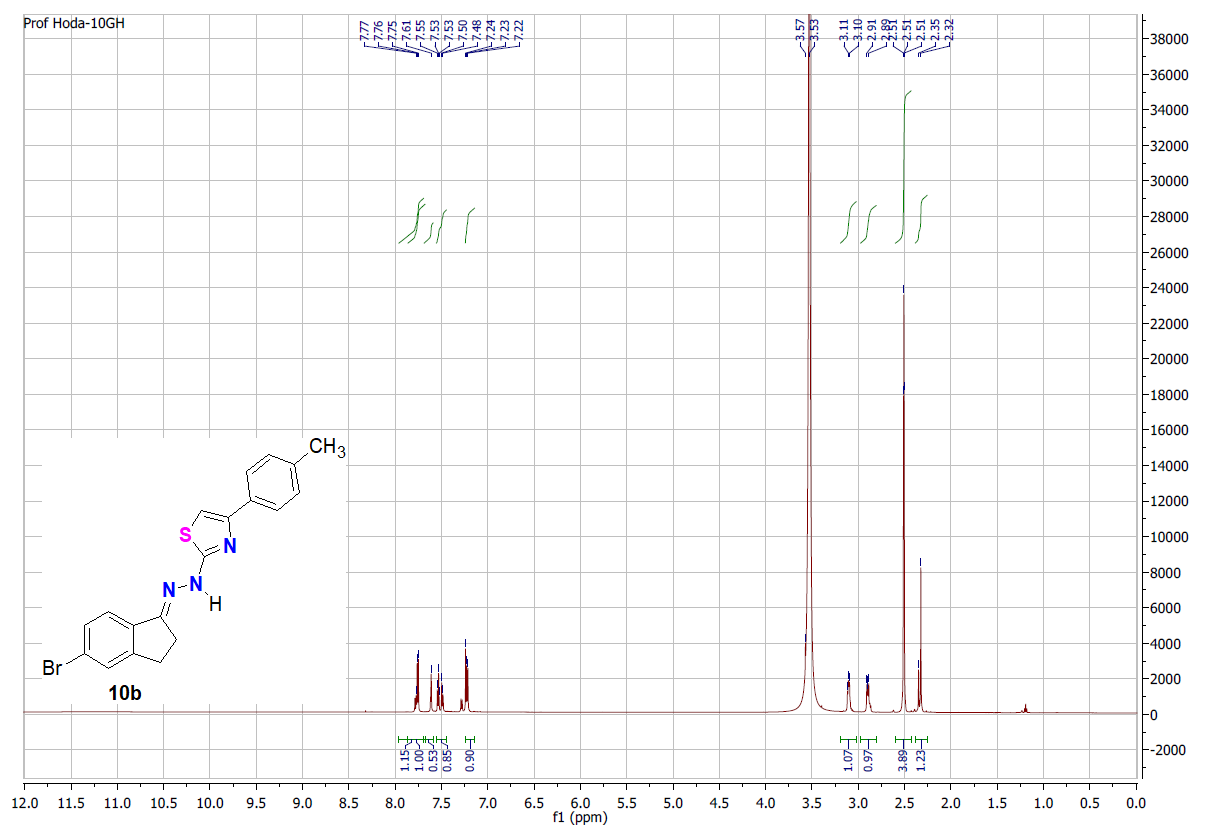


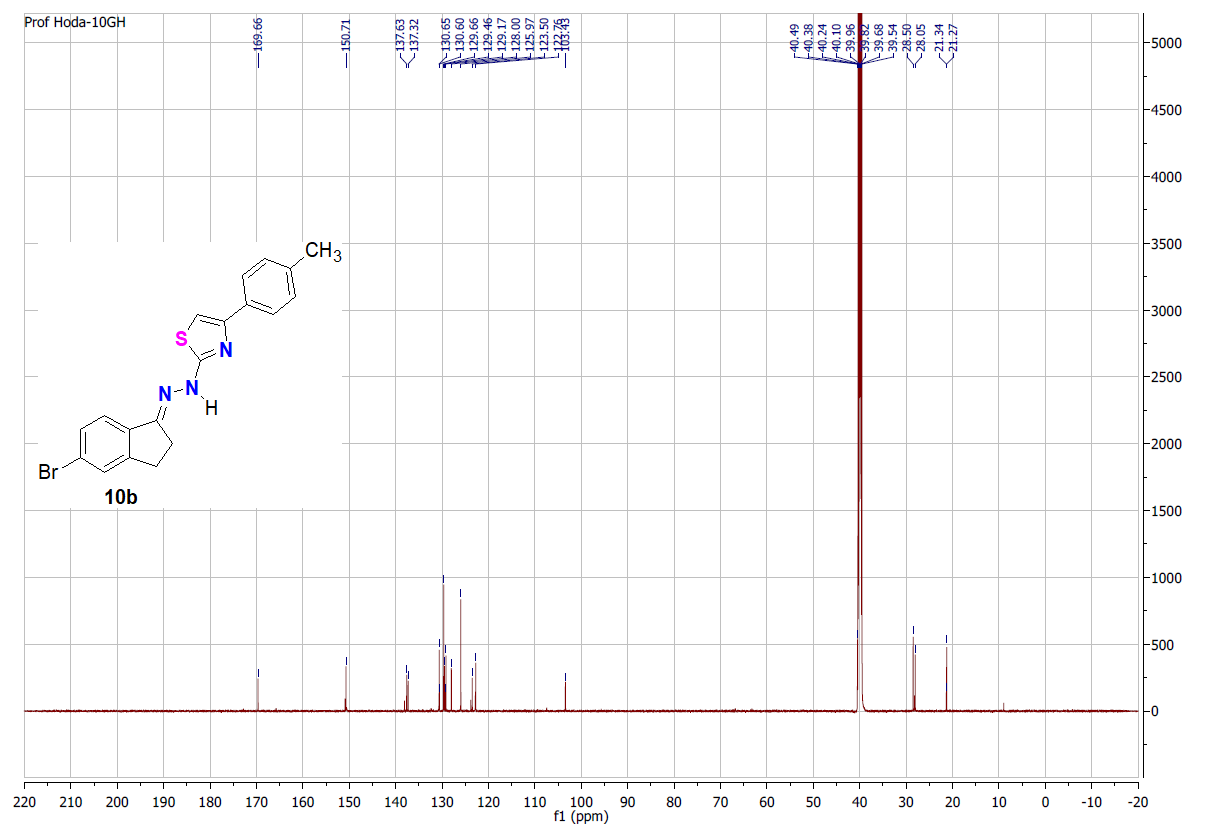


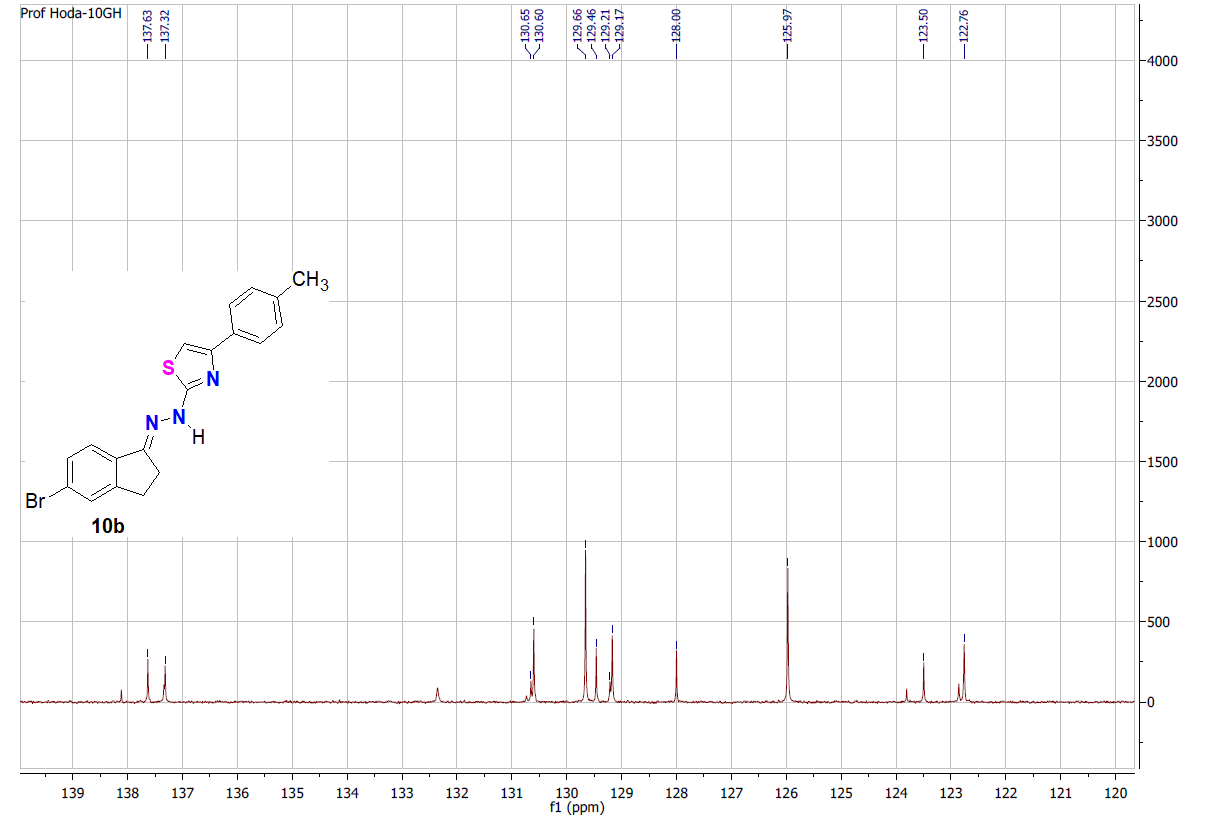


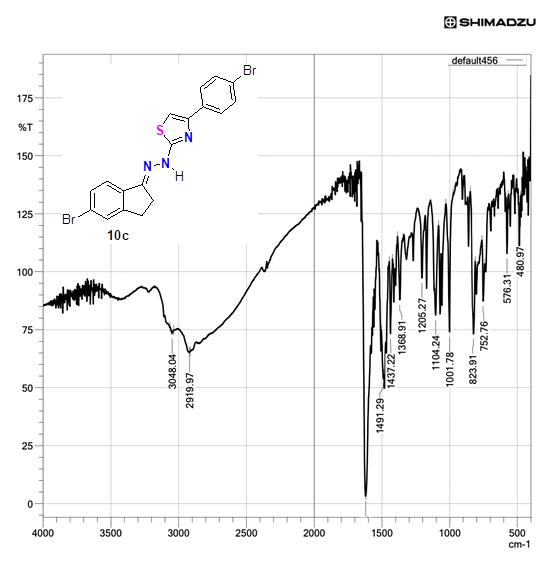


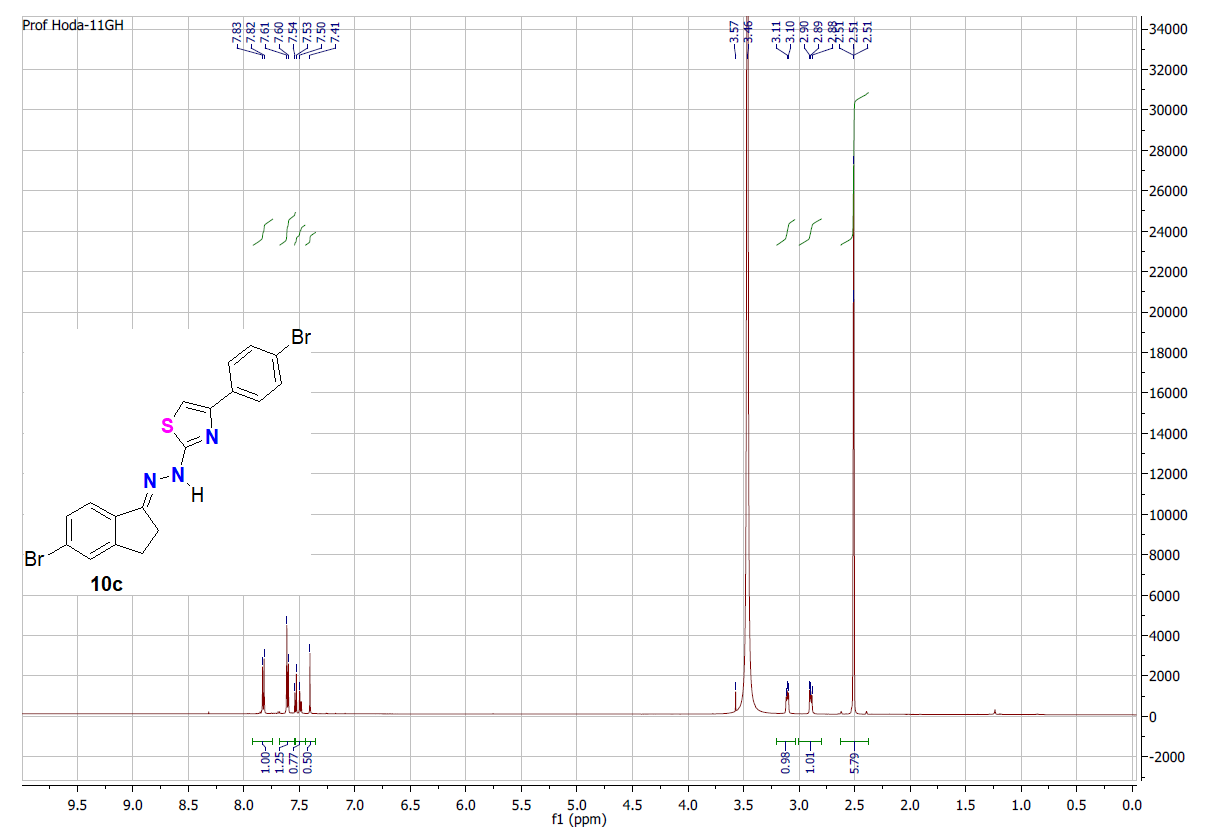


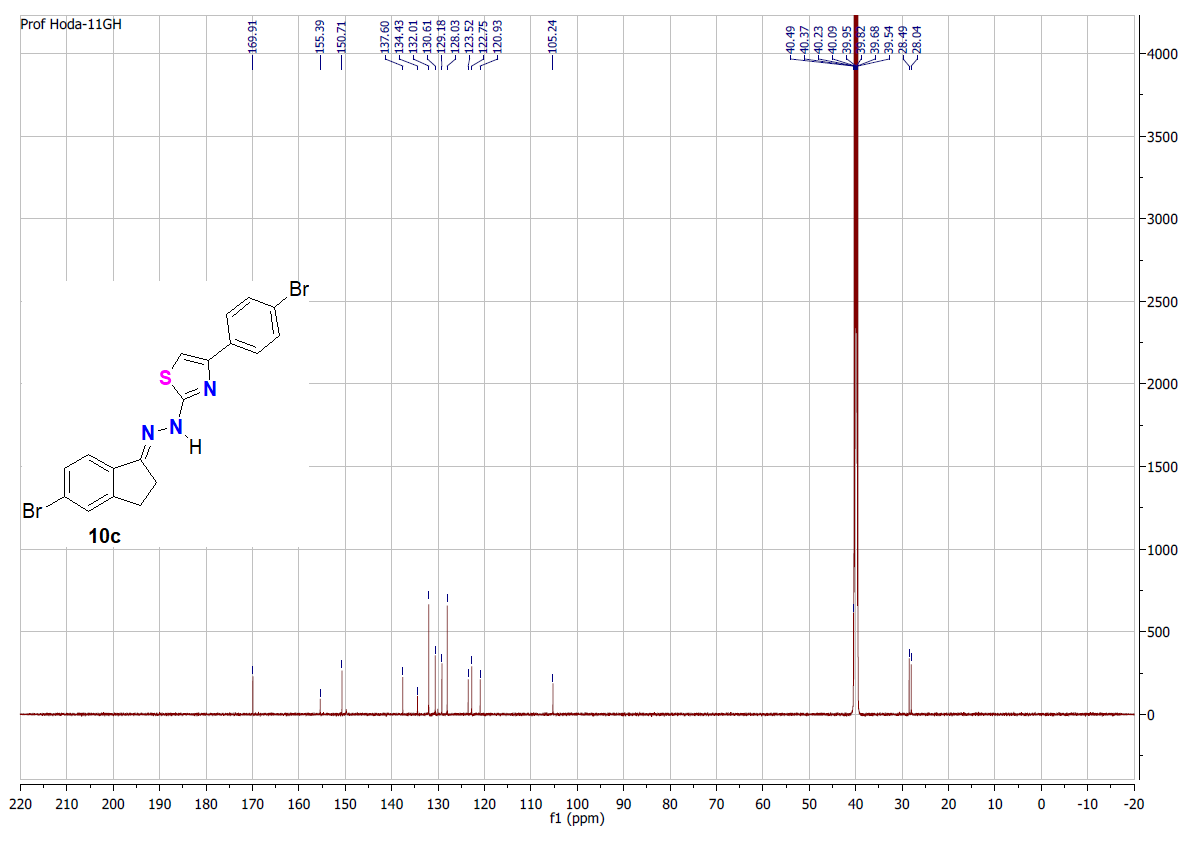


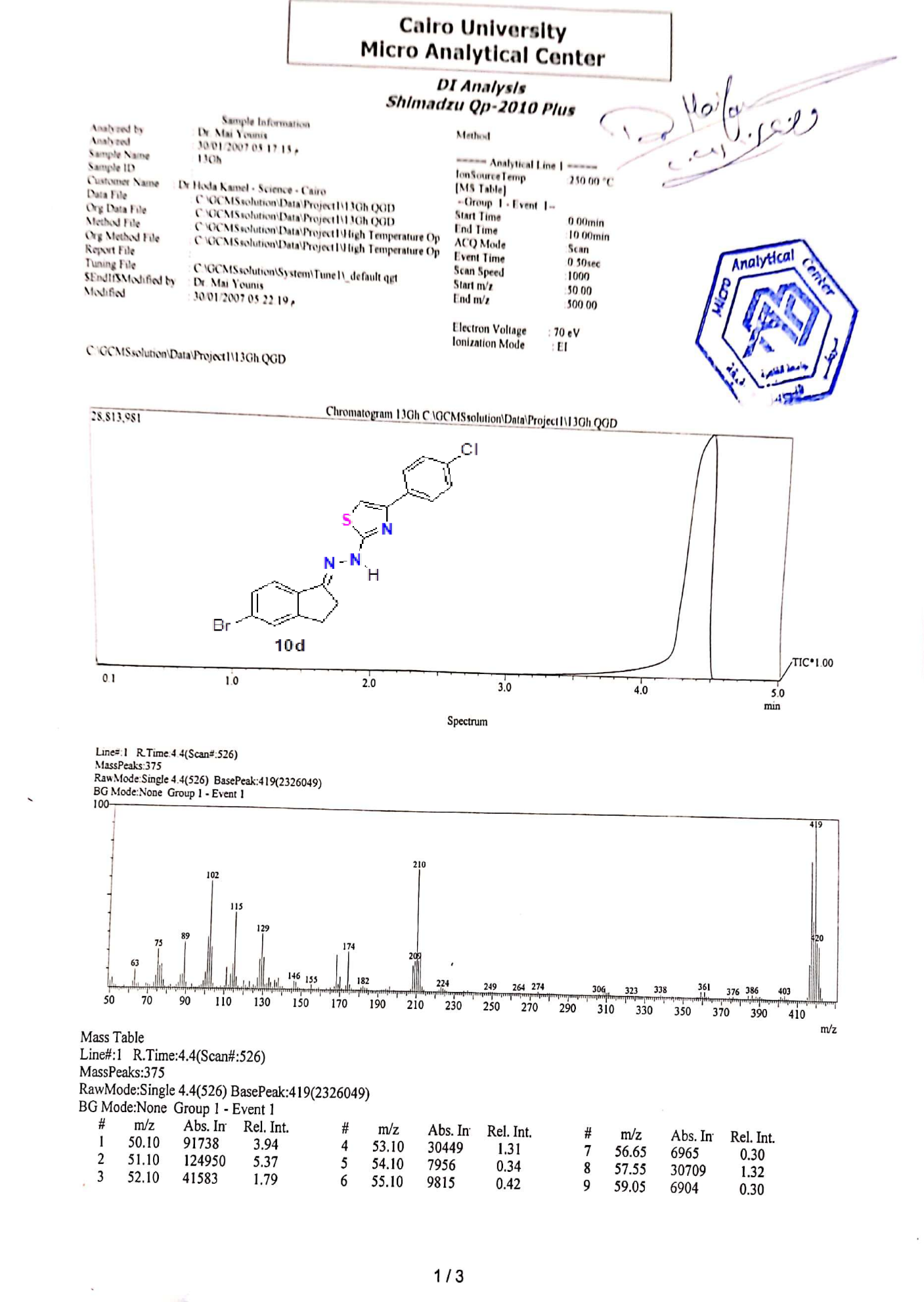


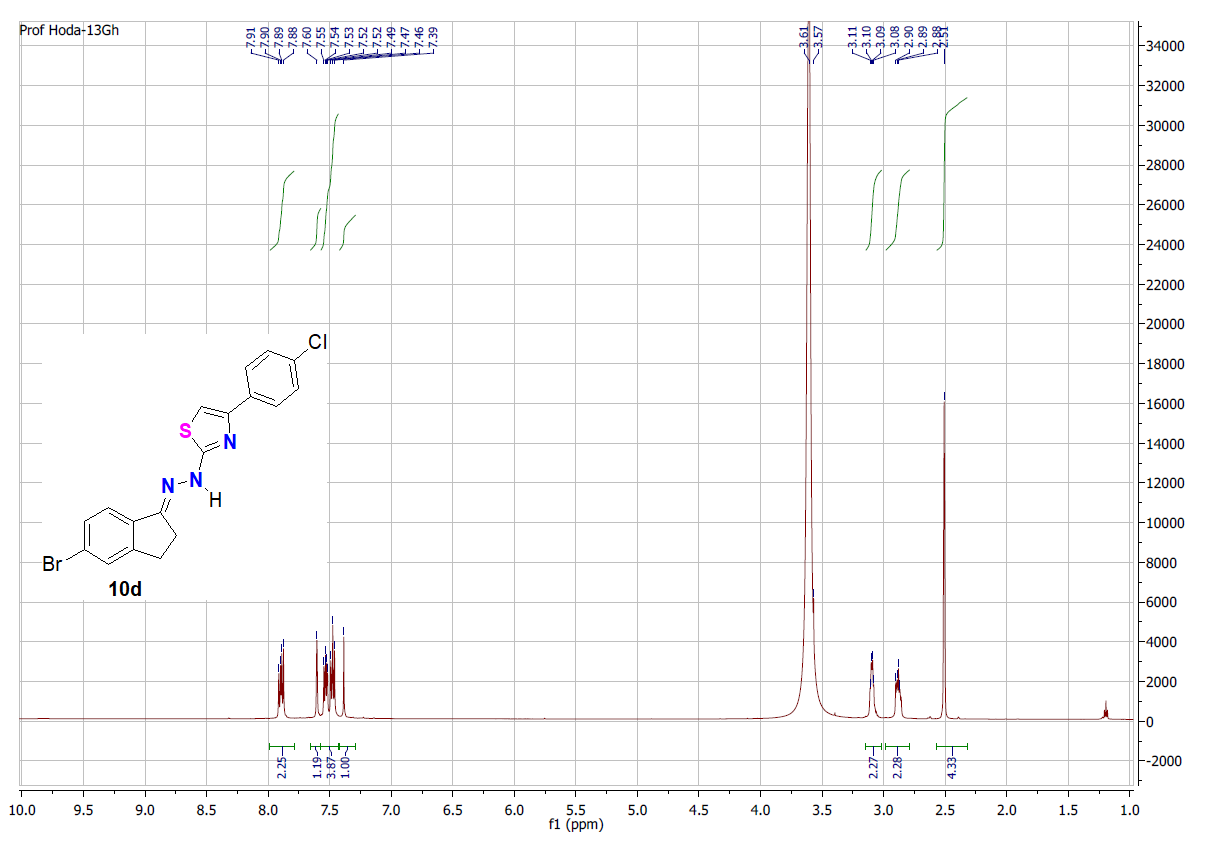


**
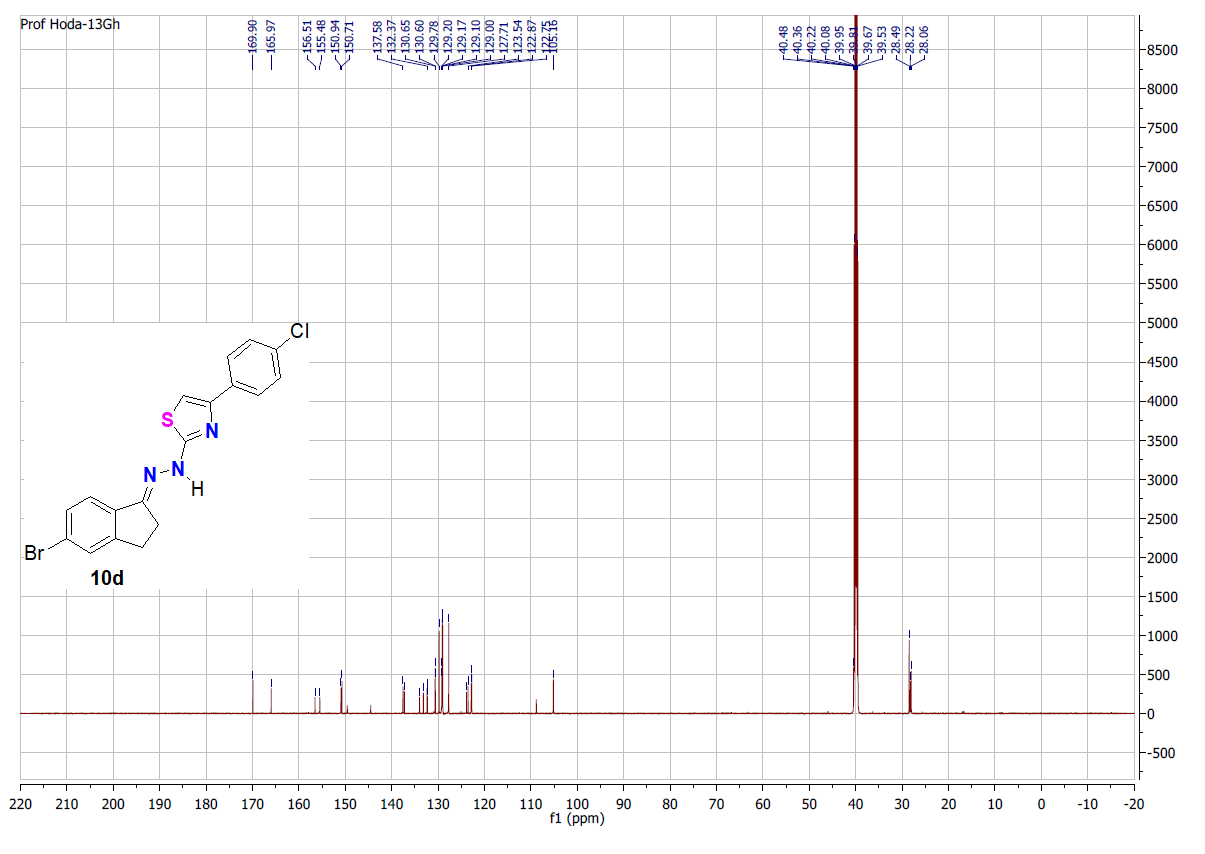
**


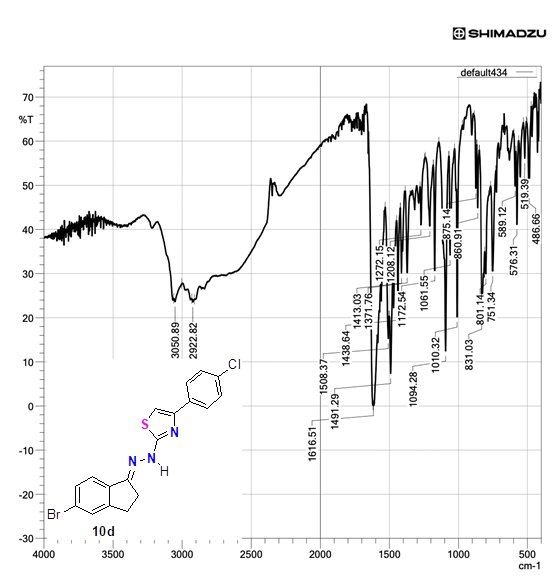


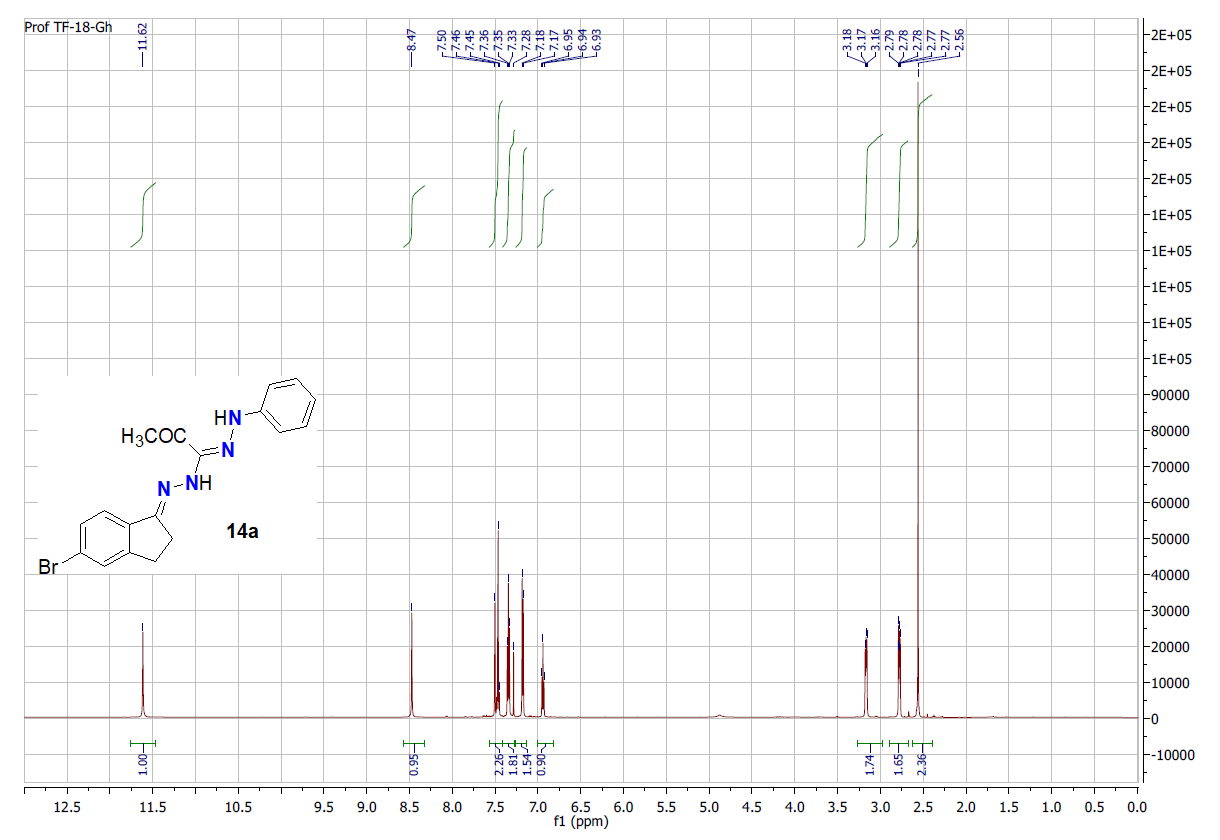

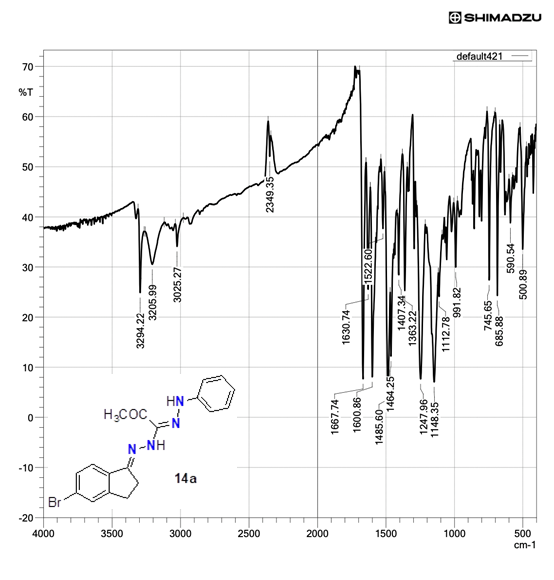


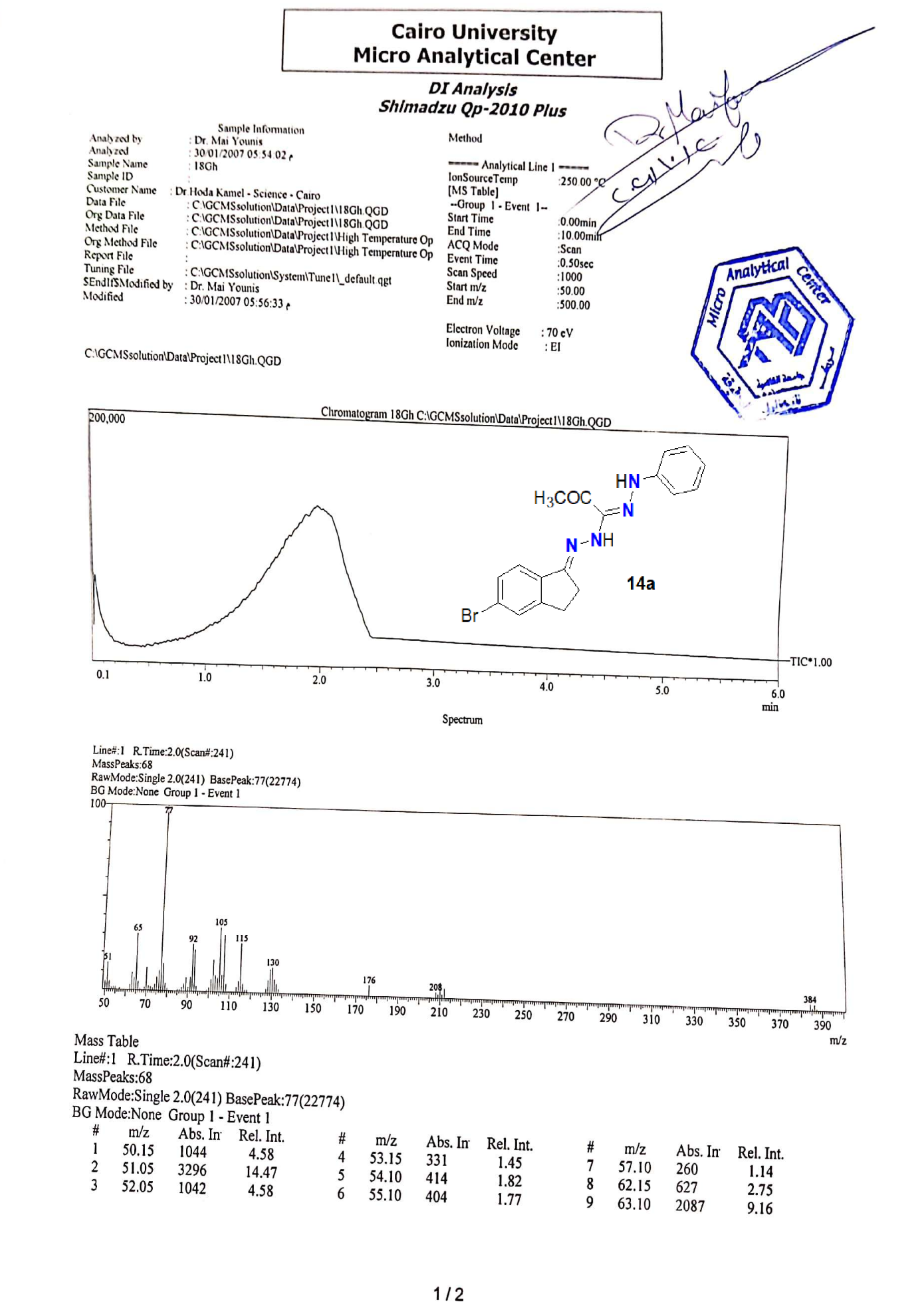


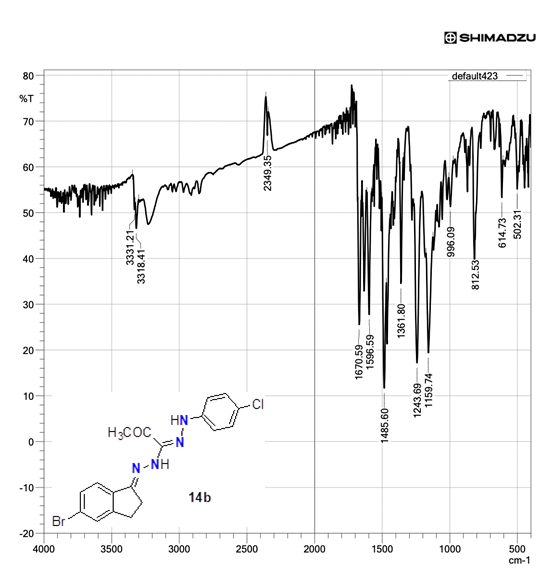


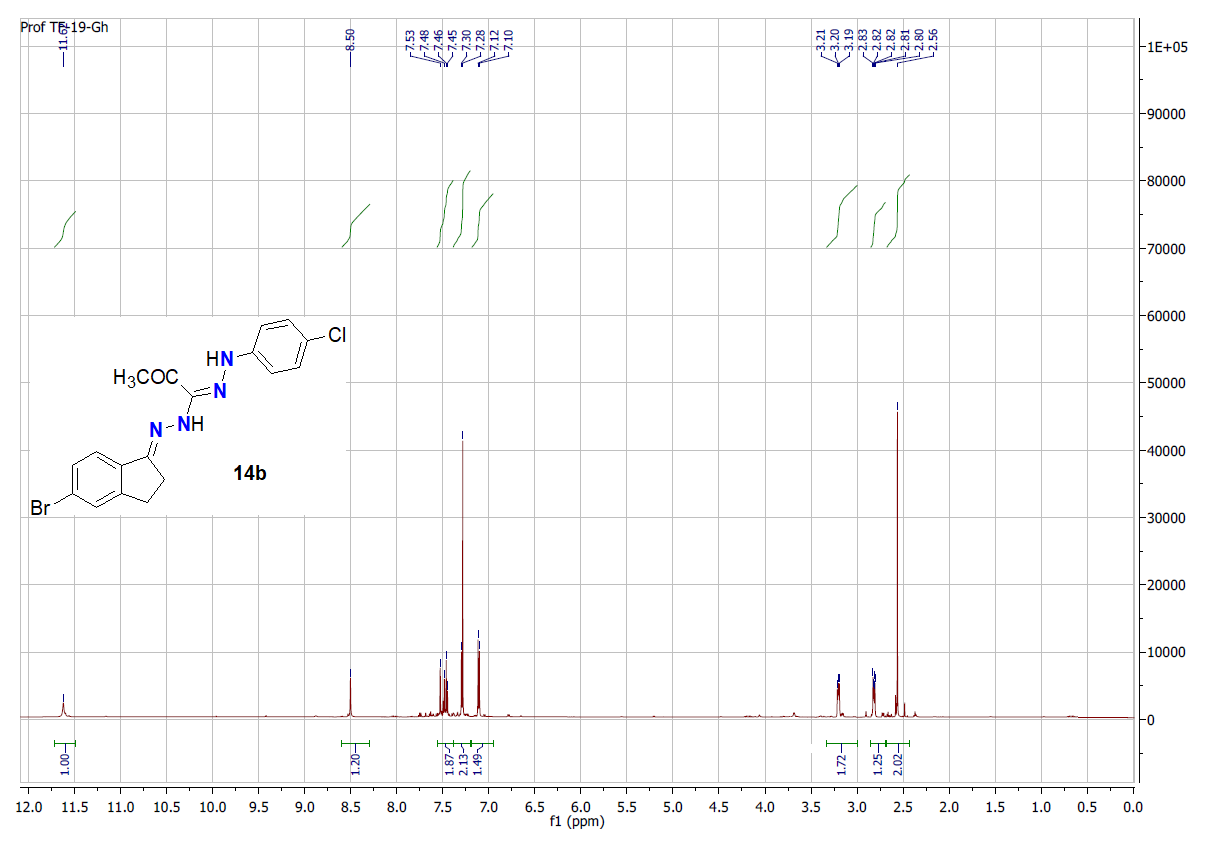


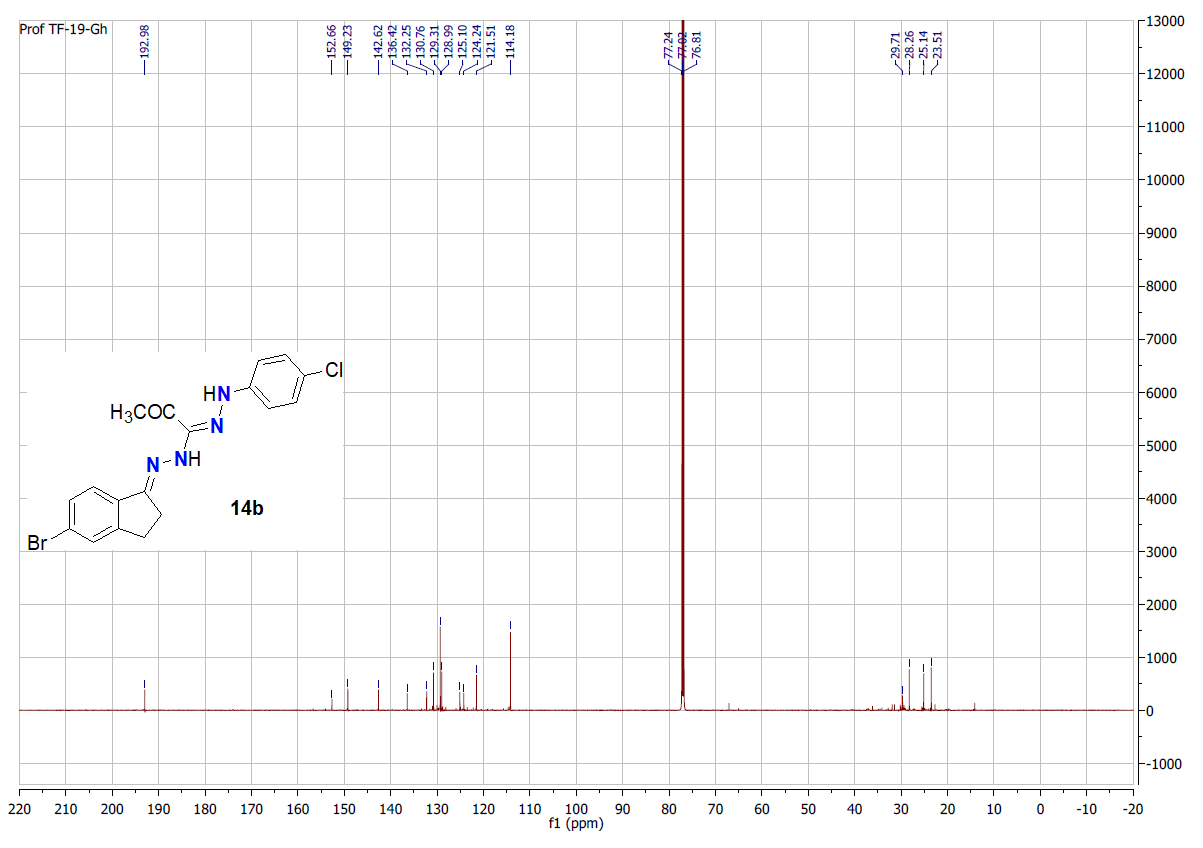


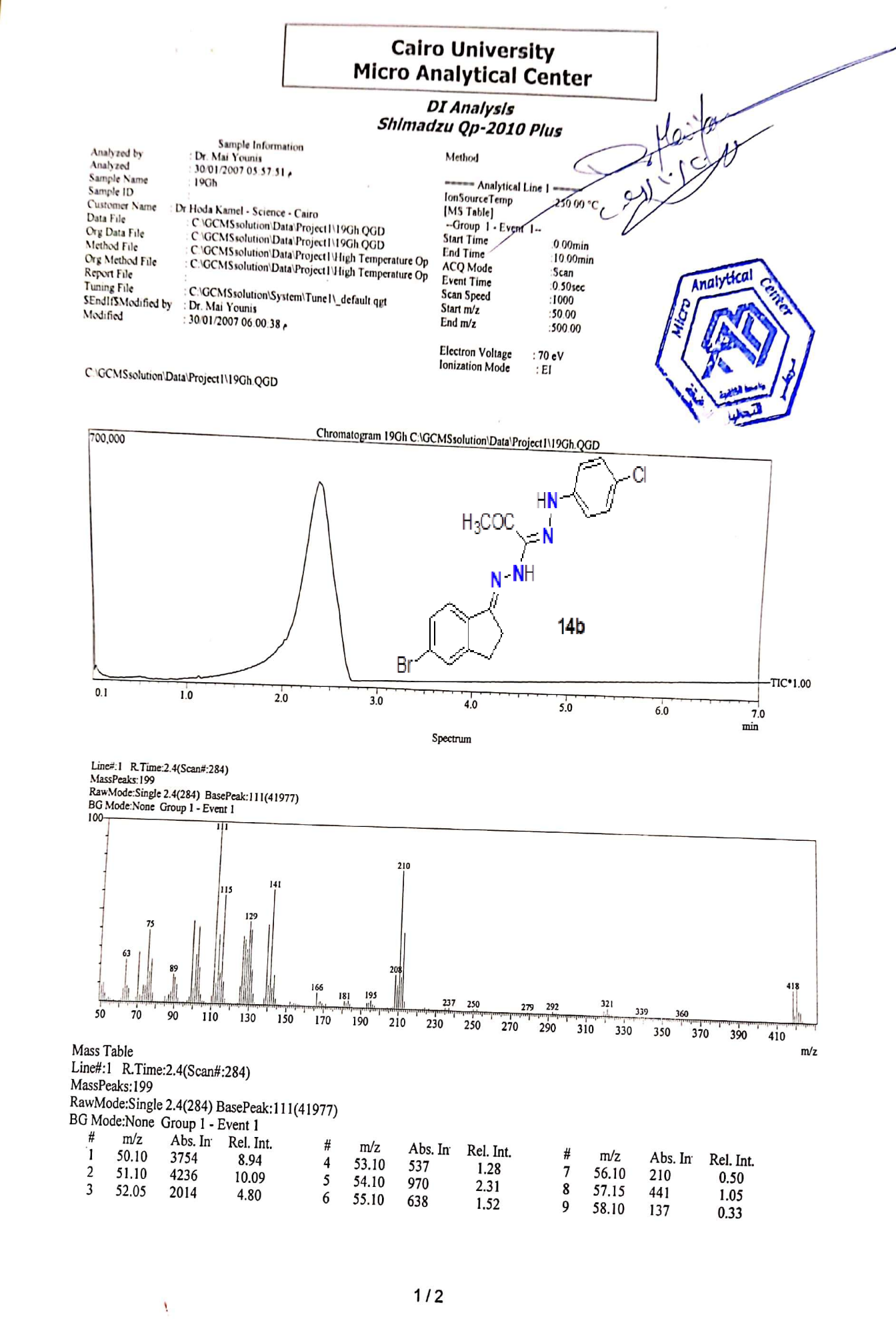


**
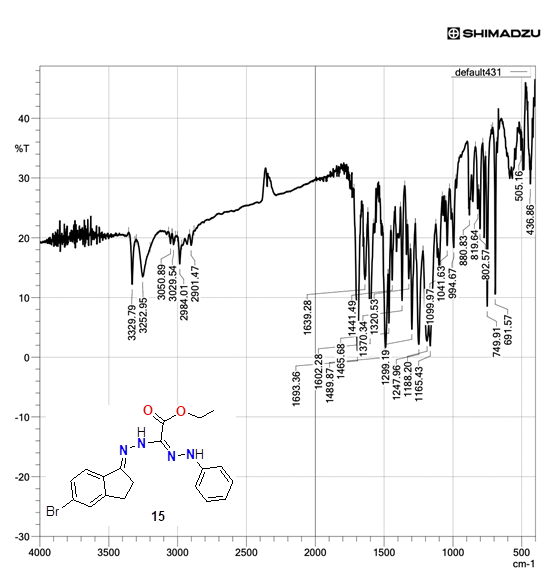
**


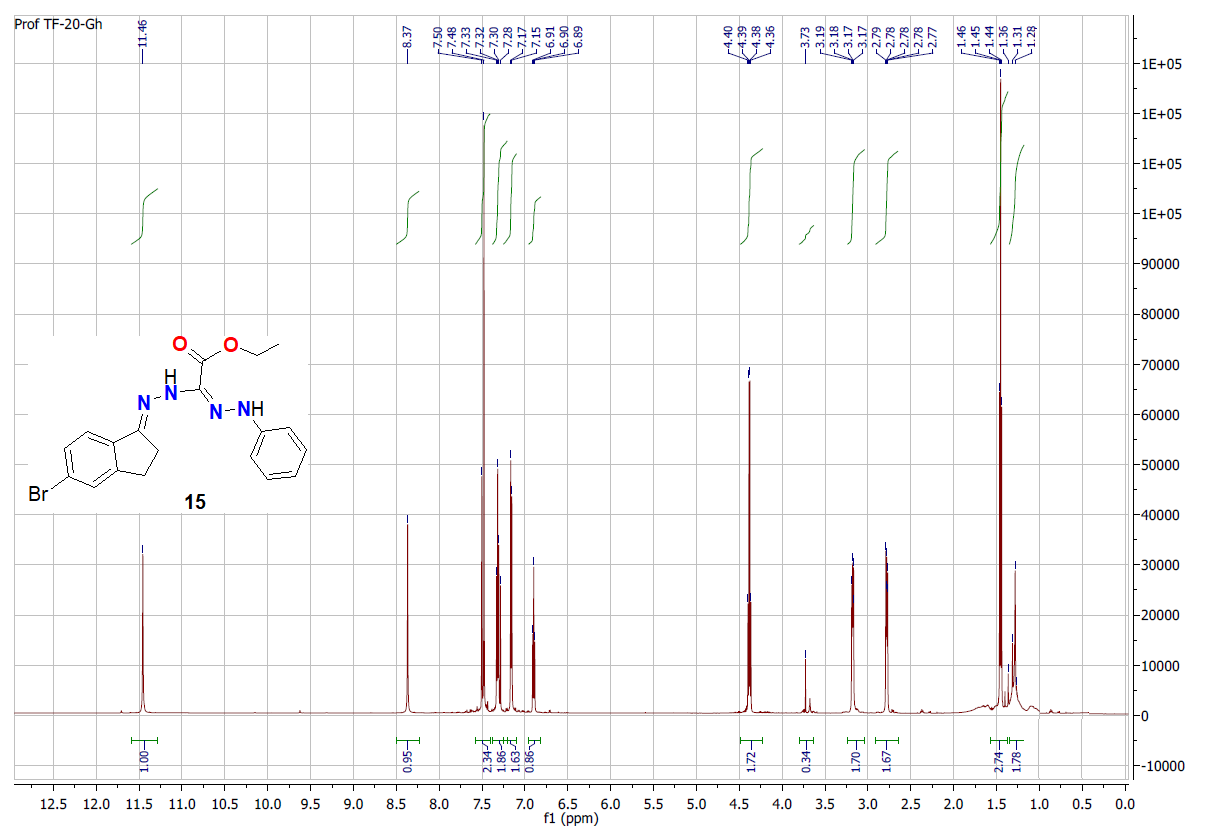

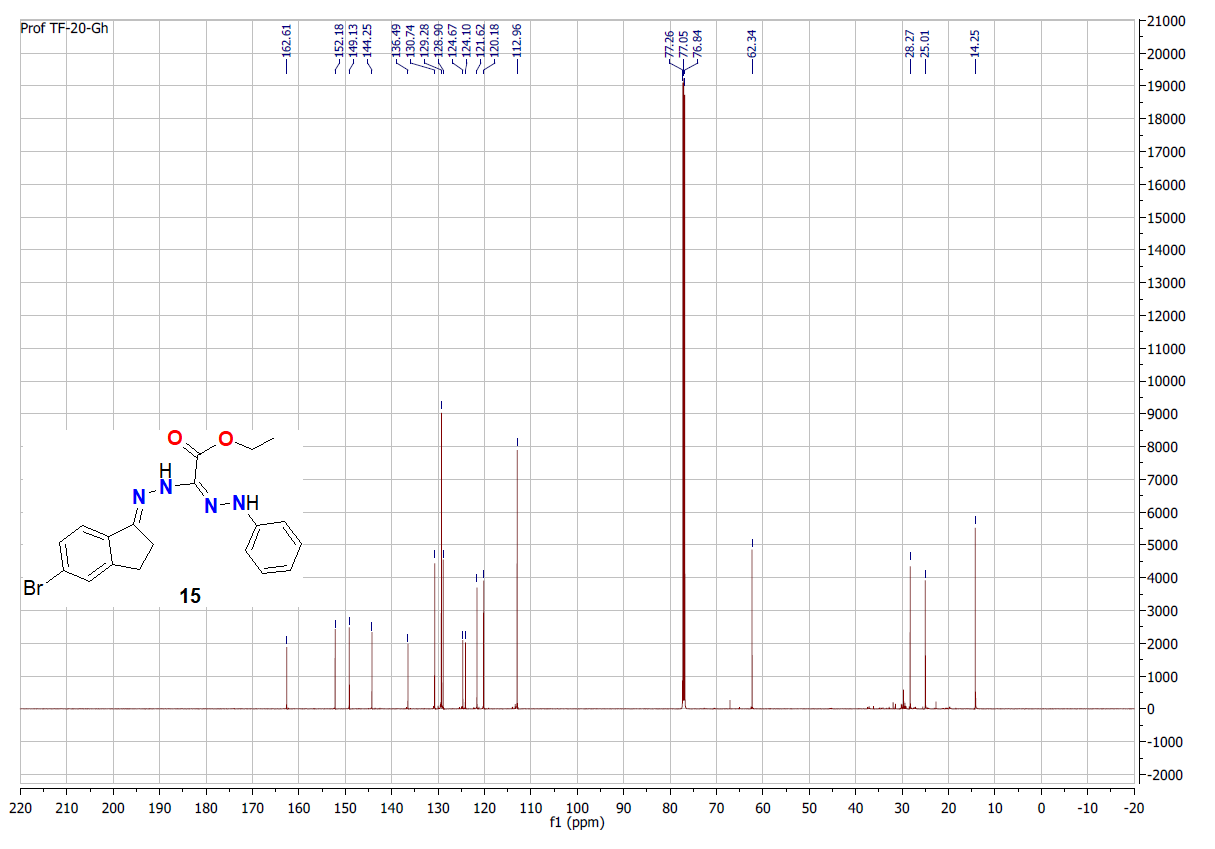


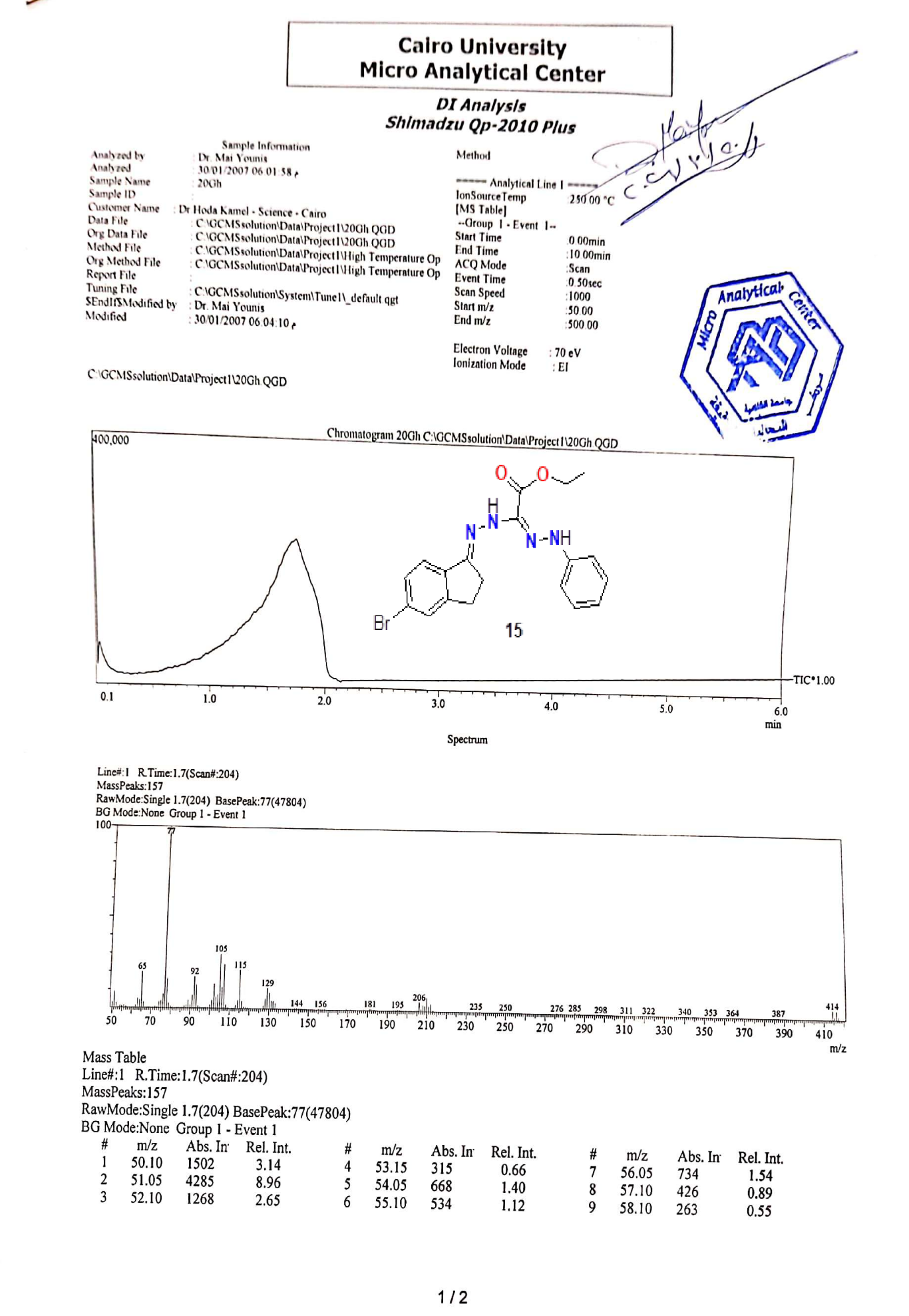


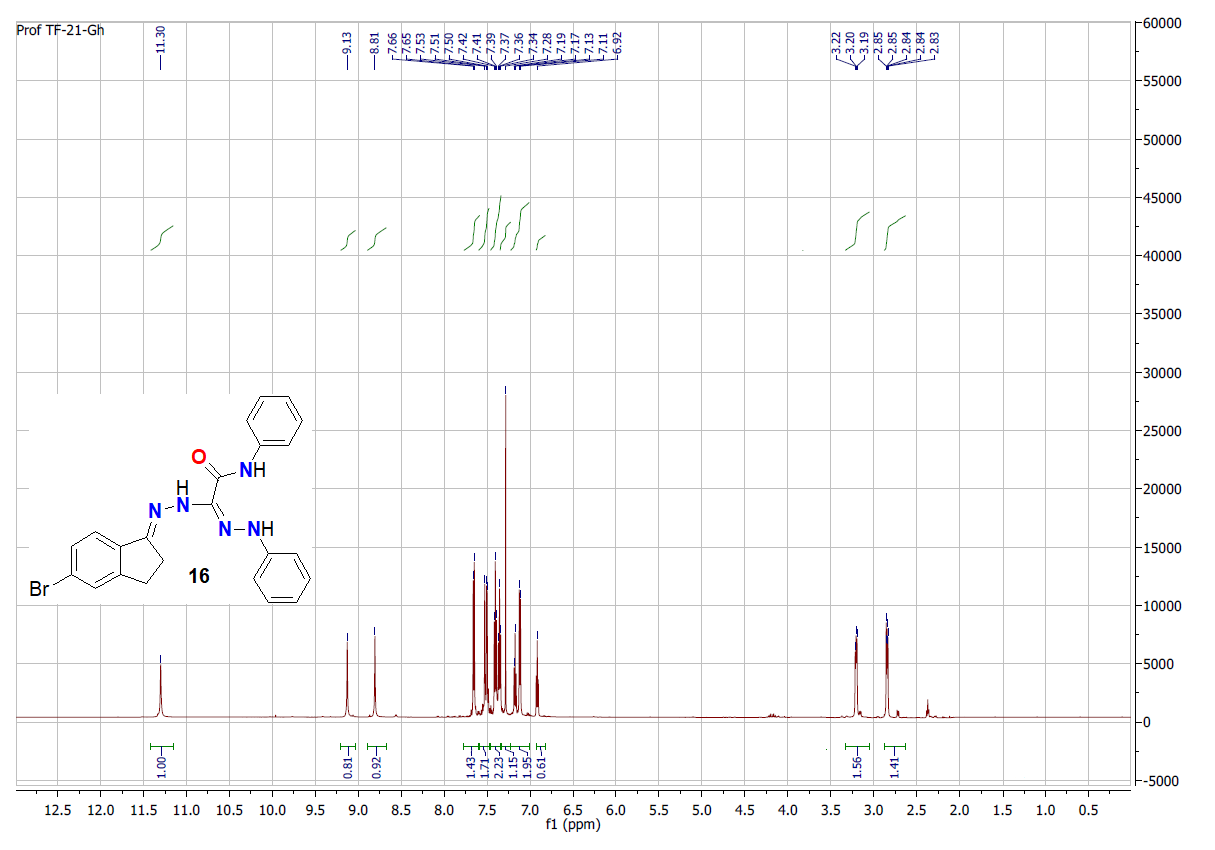

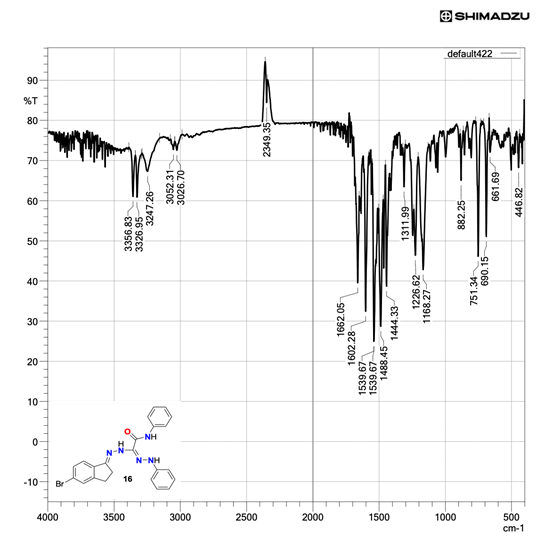


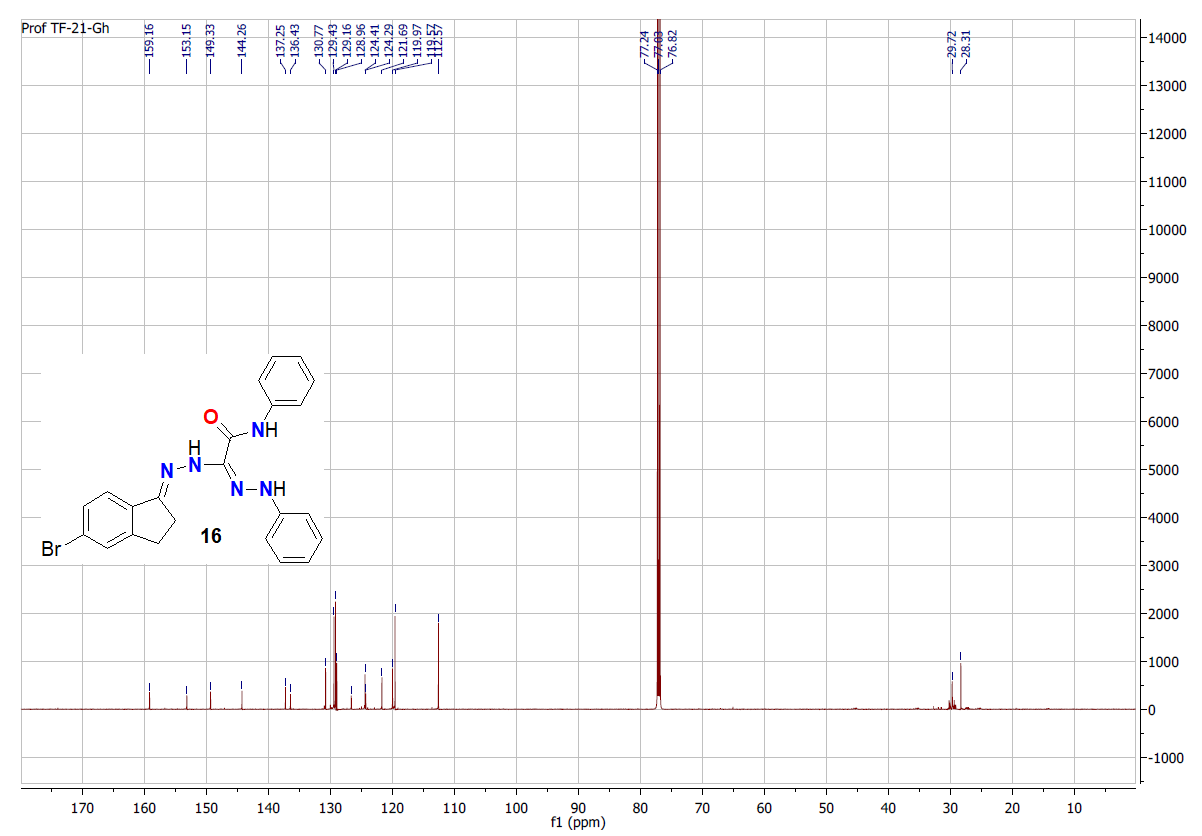


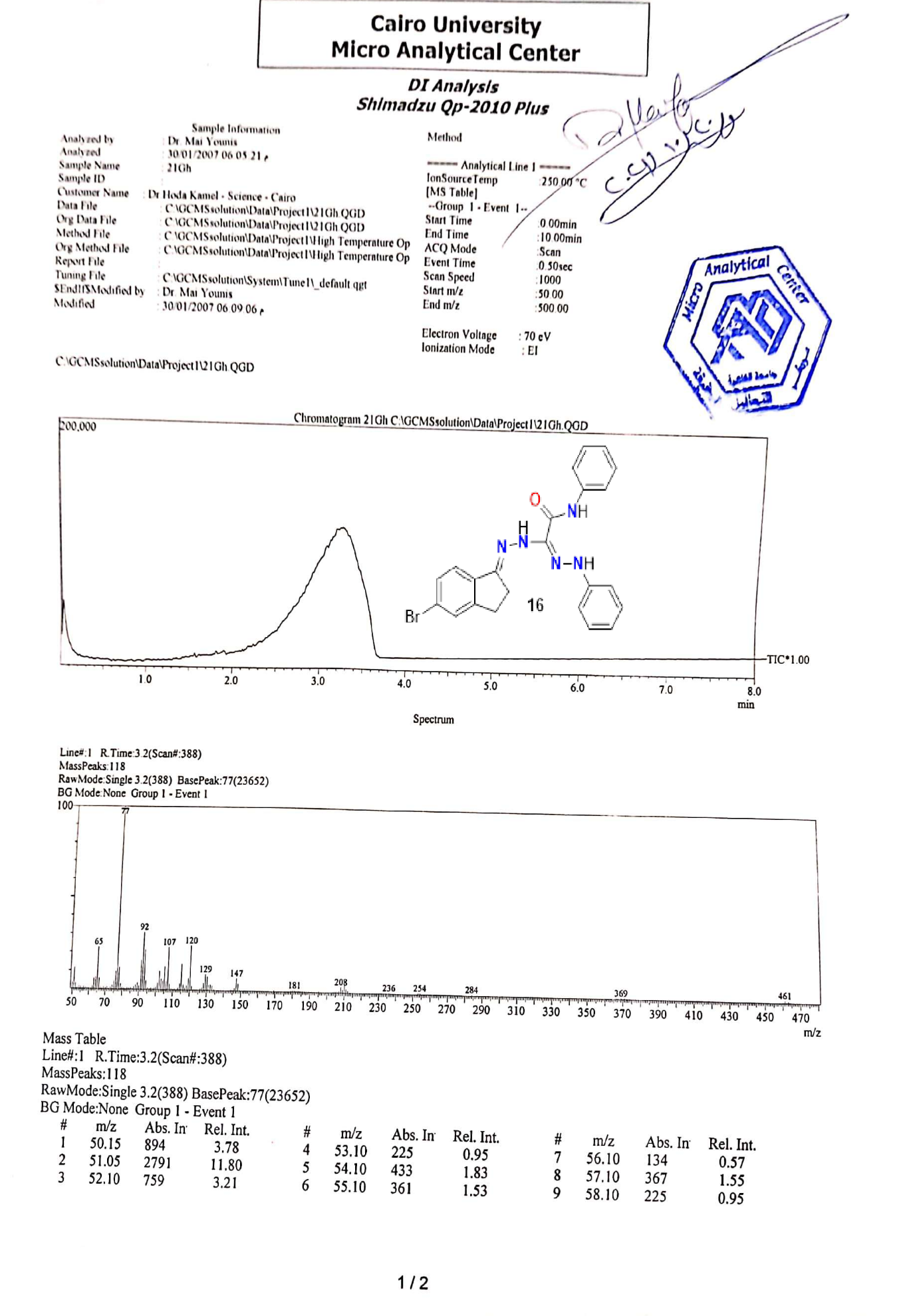


| COLON cancer : COLO205 | | | | | | | |
| --- | --- | --- | --- | --- | --- | --- | --- |
| **3** | **conc** | **Run1** | **Run2** | **Run3** | **mean** | **% Viability** | **% Inhibition** |
|  | **0** | 0.981 | 0.976 | 0.992 | 0.983 | 100 | 0 |
|  | **6.25** | 0.837 | 0.387 | 0.837 | 0.837 | 85.13 | 14.87 |
|  | **12.5** | 0.533 | 0.524 | 0.518 | 0.525 | 53.42 | 46.58 |
|  | **25** | 0.354 | 0.354 | 0.354 | 0.354 | 36.04 | 63.96 |
|  | **50** | 0.257 | 0.251 | 0.251 | 0.253 | 25.77 | 74.23 |
|  | **100** | 0.072 | 0.072 | 0.072 | 0.072 | 7.86 | 92.14 |
| **4** | **conc** | **Run1** | **Run2** | **Run3** | **mean** | **% Viability** | **% Inhibition** |
|  | **0** | 1.71 | 1.75 | 1.69 | 1.73 | 100 | 0 |
|  | **6.25** | 0.981 | 0.981 | 0.981 | 0.981 | 56.74 | 43.26 |
|  | **12.5** | 0.761 | 0.763 | 0.765 | 0.763 | 43.88 | 56.12 |
|  | **25** | 0.45 | 0.441 | 0.441 | 0.444 | 25.64 | 74.36 |
|  | **50** | 0.188 | 0.188 | 0.188 | 0.188 | 10.86 | 89.14 |
|  | **100** | 0.145 | 0.148 | 0.148 | 0.147 | 8.5 | 91.5 |
| **6a** | **conc** | **Run1** | **Run2** | **Run3** | **mean** | **% Viability** | **% Inhibition** |
|  | **0** | 0.91 | 0.912 | 0.908 | 0.91 | 100 | 0 |
|  | **6.25** | 0.779 | 0.771 | 0.772 | 0.774 | 85.1 | 14.9 |
|  | **12.5** | 0.603 | 0.609 | 0.606 | 0606 | 66.6 | 33.4 |
|  | **25** | 0.307 | 0.309 | 0.308 | 0.308 | 33.89 | 66.11 |
|  | **50** | 0.201 | 0.203 | 0.214 | 0.206 | 22.61 | 77.39 |
|  | **100** | 0.06 | 0.06 | 0.06 | 0.06 | 6.55 | 93.45 |
| **6b** | **conc** | **Run1** | **Run2** | **Run3** | **mean** | **% Viability** | **% Inhibition** |
|  | **0** | 1.91 | 1.91 | 1.91 | 1.91 | 100 | 0 |
|  | **6.25** | 1.28 | 1.23 | 1.6 | 1.29 | 67.4 | 32.6 |
|  | **12.5** | 0.88 | 0.88 | 0.88 | 0.88 | 46.07 | 53.96 |
|  | **25** | 0.394 | 0.397 | 0.4 | 0.397 | 20.8 | 79.2 |
|  | **50** | 0.181 | 0.174 | 0.173 | 0.176 | 9.19 | 90.81 |
|  | **100** | 0.129 | 0.124 | 0.122 | 0.125 | 6.55 | 93.45 |
| **6c** | **conc** | **Run1** | **Run2** | **Run3** | **mean** | **% Viability** | **% Inhibition** |
|  | **0** | 1.31 | 1.301 | 1.301 | 1.304 | 100 | 0 |
|  | **6.25** | 0.76 | 0.74 | 0.78 | 0.76 | 58.31 | 41.69 |
|  | **12.5** | 0.56 | 0.56 | 0.56 | 0.56 | 41.67 | 58.33 |
|  | **25** | 0.272 | 0.275 | 0.269 | 0.272 | 20.86 | 79.14 |
|  | **50** | 0.099 | 0.099 | 0.099 | 0.099 | 7.6 | 92.4 |
|  | **100** | 0.063 | 0.061 | 0.062 | 0.062 | 4.7 | 95.3 |
| **6d** | **conc** | **Run1** | **Run2** | **Run3** | **mean** | **% Viability** | **% Inhibition** |
|  | **0** | 1.59 | 1.63 | 1.61 | 1.61 | 100 | 0 |
|  | **6.25** | 0.96 | 0.93 | 0.93 | 0.94 | 58.4 | 41.6 |
|  | **12.5** | 0.742 | 0.742 | 0.742 | 0.742 | 46.1 | 53.9 |
|  | **25** | 0.505 | 0.505 | 0.505 | 0.505 | 31.4 | 68.6 |
|  | **50** | 0.181 | 0.163 | 0.172 | 0.172 | 10.7 | 89.3 |
|  | **100** | 0.089 | 0.089 | 0.089 | 0.089 | 5.5 | 94.5 |
| **6e** | **conc** | **Run1** | **Run2** | **Run3** | **mean** | **% viability** | **% inhibition** |
|  | **0** | 1.098 | 1.092 | 1.092 | 1.094 | 100 | 0 |
|  | **6.25** | 0.626 | 0.62 | 0.62 | 0.622 | 56.88 | 43.12 |
|  | **12.5** | 0.421 | 0.421 | 0.421 | 0.421 | 38.47 | 61.53 |
|  | **25** | 0.149 | 0.149 | 0.149 | 0.149 | 13.6 | 86.4 |
|  | **50** | 0.073 | 0.07 | 0.071 | 0.071 | 6.53 | 93.47 |
|  | **100** | 0.048 | 0.048 | 0.048 | 0.048 | 4.34 | 95.66 |
| **10a** | **conc** | **Run1** | **Run2** | **Run3** | **mean** | **% Viability** | **% Inhibition** |
|  | **0** | 0.772 | 0.783 | 0.788 | 0.781 | 100 | 0 |
|  | **6.25** | 0.401 | 0.44 | 0.449 | 0.43 | 54.92 | 45.08 |
|  | **12.5** | 0.38 | 0.36 | 0.40 | 0.38 | 48.53 | 51.47 |
|  | **25** | 0.181 | 0.181 | 0.181 | 0.181 | 23.2 | 76.8 |
|  | **50** | 0.083 | 0.089 | 0.089 | 0.087 | 11.1 | 88.9 |
|  | **100** | 0.045 | 0.047 | 0.046 | 0.046 | 5.99 | 94.01 |
| **10b** | **conc** | **Run1** | **Run2** | **Run3** | **mean** | **% Viability** | **% Inhibition** |
|  | **0** | 0.7 | 0.7 | 0.7 | 0.7 | 100 | 0 |
|  | **6.25** | 0.54 | 0.51 | 0.48 | 0.51 | 72.84 | 27.16 |
|  | **12.5** | 0.373 | 0.375 | 0.374 | 0.374 | 53.42 | 46.58 |
|  | **25** | 0.163 | 0.151 | 0.166 | 0.16 | 22.88 | 77.12 |
|  | **50** | 0.077 | 0.077 | 0.077 | 0.077 | 10.94 | 89.06 |
|  | **100** | 0.039 | 0.036 | 0.039 | 0.038 | 5.45 | 94.55 |
| **10c** | **conc** | **Run1** | **Run2** | **Run3** | **mean** | **% Viability** | **% Inhibition** |
|  | **0** | 1.2 | 1.007 | 0.85 | 1.019 | 100 | 0 |
|  | **6.25** | 0.845 | 0.845 | 0.845 | 0.845 | 82.98 | 17.02 |
|  | **12.5** | 0.524 | 0.521 | 0.536 | 0.527 | 51.75 | 48.25 |
|  | **25** | 0.346 | 0.355 | 0.355 | 0.352 | 34.57 | 65.43 |
|  | **50** | 0.25 | 0.25 | 0.25 | 0.25 | 24.52 | 75.48 |
|  | **100** | 0.034 | 0.036 | 0.032 | 0.034 | 3.36 | 93.64 |
| **10d** | **conc** | **Run1** | **Run2** | **Run3** | **mean** | **% Viability** | **% Inhibition** |
|  | **0** | 0.984 | 0.97 | 1.004 | 0.986 | 100 | 0 |
|  | **6.25** | 0.724 | 0.724 | 0.724 | 0.724 | 73.47 | 26.53 |
|  | **12.5** | 0.502 | 0.502 | 0.502 | 0.502 | 50.91 | 49.09 |
|  | **25** | 0.264 | 0.264 | 0.264 | 0.264 | 26.78 | 73.22 |
|  | **50** | 0.085 | 0.093 | 0.093 | 0.09 | 9.09 | 90.91 |
|  | **100** | 0.058 | 0.054 | 0.056 | 0.056 | 5.64 | 94.36 |
| **14a** | **conc** | **Run1** | **Run2** | **Run3** | **mean** | **% Viability** | **% Inhibition** |
|  | **0** | 0.896 | 0.81 | 0.967 | 0.891 | 100 | 0 |
|  | **6.25** | 0.664 | 0.803 | 0.684 | 0.717 | 80.52 | 19.48 |
|  | **12.5** | 0.404 | 0.408 | 0.406 | 0.406 | 45.67 | 54.33 |
|  | **25** | 0.162 | 0.179 | 0.169 | 0.17 | 18.96 | 81.04 |
|  | **50** | 0.067 | 0.067 | 0.067 | 0.067 | 7.51 | 92.49 |
|  | **100** | 0.033 | 0.033 | 0.033 | 0.033 | 3.66 | 96.34 |
| **14b** | **conc** | **Run1** | **Run2** | **Run3** | **mean** | **% Viability** | **% Inhibition** |
|  | **0** | 0.706 | 0.713 | 0.759 | 0.726 | 100 | 0 |
|  | **6.25** | 0.556 | 0.556 | 0.556 | 0.556 | 76.6 | 23.4 |
|  | **12.5** | 0.315 | 0.312 | 0.313 | 0.313 | 43.16 | 56.84 |
|  | **25** | 0.152 | 0.152 | 0.152 | 0.152 | 21.04 | 78.96 |
|  | **50** | 0.057 | 0.073 | 0.059 | 0.063 | 8.74 | 91.26 |
|  | **100** | 0.029 | 0.023 | 0.038 | 0.03 | 4.13 | 95.87 |
| **15** | **conc** | **Run1** | **Run2** | **Run3** | **mean** | **% Viability** | **% Inhibition** |
|  | **0** | 0.716 | 0.713 | 0.759 | 0.73 | 100 | 0 |
|  | **6.25** | 0.587 | 0.582 | 0.563 | 0.577 | 79.04 | 20.95 |
|  | **12.5** | 0.425 | 0.427 | 0.421 | 0.424 | 58.08 | 41.92 |
|  | **25** | 0.172 | 0.162 | 0.134 | 0.156 | 21.4 | 78.63 |
|  | **50** | 0.067 | 0.073 | 0.059 | 0.066 | 9.04 | 90.96 |
|  | **100** | 0.029 | 0.023 | 0.028 | 0.027 | 3.699 | 96.301 |
| **16** | **conc** | **Run1** | **Run2** | **Run3** | **mean** | **% Viability** | **% Inhibition** |
|  | **0** | 0.921 | 0.934 | 0.933 | 0.93 | 100 | 0 |
|  | **6.25** | 0.742 | 0.716 | 0.718 | 0.725 | 77.96 | 22.04 |
|  | **12.5** | 0.529 | 0.511 | 0.533 | 0.524 | 56.34 | 43.66 |
|  | **25** | 0.183 | 0.181 | 0.181 | 0.182 | 19.57 | 80.43 |
|  | **50** | 0.057 | 0.073 | 0.059 | 0.063 | 6.77 | 93.23 |
|  | **100** | 0.045 | 0.047 | 0.042 | 0.045 | 4.84 | 95.16 |
| **cis** | **conc** | **Run1** | **Run2** | **Run3** | **mean** | **% Viability** | **% Inhibition** |
|  | **0** | 0.896 | 0.81 | 0.967 | 0.891 | 100 | 0 |
|  | **6.25** | 0.664 | 0.803 | 0.684 | 0.717 | 80.52 | 19.48 |
|  | **12.5** | 0.404 | 0.408 | 0.406 | 0.406 | 45.67 | 54.33 |
|  | **25** | 0.162 | 0.179 | 0.169 | 0.17 | 18.96 | 81.04 |
|  | **50** | 0.067 | 0.067 | 0.067 | 0.067 | 7.51 | 92.49 |
|  | **100** | 0.033 | 0.033 | 0.033 | 0.033 | 3.66 | 96.34 |
| **ref** | **conc** | **Run1** | **Run2** | **Run3** | **mean** | **% Viability** | **% Inhibition** |
|  | **0** | 0.931 | 0.931 | 0.931 | 0.931 | 100 | 0 |
|  | **6.25** | 0.617 | 0.617 | 0.617 | 0.617 | 66.31 | 33.69 |
|  | **12.5** | 0.343 | 0.343 | 0.343 | 0.343 | 36.89 | 63.11 |
|  | **25** | 0.176 | 0.176 | 0.176 | 0.176 | 18.94 | 81.06 |
|  | **50** | 0.037 | 0.037 | 0.037 | 0.037 | 4.01 | 95.99 |
|  | **100** | 0.01 | 0.01 | 0.01 | 0.01 | 1.09 | 98.91 |

| **Gastric (SNU-16)**  **Patch number (ATCC CRL-5822)** | | | | | | | |
| --- | --- | --- | --- | --- | --- | --- | --- |
| **3** | **Conc.** | **Run1** | **Run2** | **Run3** | **mean** | **% Viability** | **% Inhibition** |
|  | **0** | 0.231 | 0.231 | 0.231 | 0.231 | 100 | 0 |
|  | **6.25** | 0.05 | 0.05 | 0.05 | 0.05 | 78.65 | 21.35 |
|  | **12.5** | 0.095 | 0.095 | 0.095 | 0.095 | 58.94 | 41.06 |
|  | **25** | 0.13 | 0.13 | 0.13 | 0.13 | 43.58 | 56.42 |
|  | **50** | 0.185 | 0.185 | 0.185 | 0.185 | 19.95 | 80.05 |
|  | **100** | 0.208 | 0.208 | 0.208 | 0.208 | 10.04 | 89.96 |
| **4** | **Conc.** | **Run1** | **Run2** | **Run3** | **Mean** | **% Viability** | **% Inhibition** |
|  | **0** | 0.538 | 0.538 | 0.538 | 0.538 | 100 | 0 |
|  | **6.25** | 0.085 | 0.085 | 0.085 | 0.085 | 84.34 | 15.66 |
|  | **12.5** | 0.153 | 0.153 | 0.153 | 0.153 | 71.56 | 28.44 |
|  | **25** | 0.275 | 0.275 | 0.275 | 0.275 | 48.73 | 51.27 |
|  | **50** | 0.42 | 0.42 | 0.42 | 0.42 | 21.81 | 78.19 |
|  | **100** | 0.475 | 0.475 | 0.475 | 0.475 | 11.73 | 88.27 |
| **6a** | **Conc.** | **Run1** | **Run2** | **Run3** | **Mean** | **% Viability** | **% Inhibition** |
|  | **0** | 0.629 | 0.629 | 0.629 | 0.629 | 100 | 0 |
|  | **6.25** | 0.52 | 0.52 | 0.52 | 0.52 | 82.56 | 17.44 |
|  | **12.5** | 0.42 | 0.42 | 0.42 | 0.42 | 66.86 | 33.14 |
|  | **25** | 0.276 | 0.276 | 0.276 | 0.276 | 43.87 | 56.13 |
|  | **50** | 0.13 | 0.13 | 0.13 | 0.13 | 20.92 | 79.08 |
|  | **100** | 0.053 | 0.053 | 0.053 | 0.053 | 8.31 | 91.69 |
| **6b** | **Conc.** | **Run1** | **Run2** | **Run3** | **Mean** | **% Viability** | **% Inhibition** |
|  | **0** | 0.861 | 0.861 | 0.861 | 0.861 | 100 | 0 |
|  | **6.25** | 0.203 | 0.203 | 0.203 | 0.203 | 76.32 | 23.68 |
|  | **12.5** | 0.39 | 0.39 | 0.39 | 0.39 | 54.61 | 45.39 |
|  | **25** | 0.615 | 0.615 | 0.615 | 0.615 | 28.55 | 71.45 |
|  | **50** | 0.712 | 0.712 | 0.712 | 0.712 | 17.21 | 82.79 |
|  | **100** | 0.78 | 0.78 | 0.78 | 0.78 | 9.39 | 90.61 |
| **6c** | **Conc.** | **Run1** | **Run2** | **Run3** | **mean** | **% Viability** | **% Inhibition** |
|  | **0** | 0.59 | 0.59 | 0.59 | 0.59 | 100 | 0 |
|  | **6.25** | 0.14 | 0.14 | 0.14 | 0.14 | 75.92 | 24.08 |
|  | **12.5** | 0.254 | 0.254 | 0.254 | 0.254 | 56.94 | 43.06 |
|  | **25** | 0.414 | 0.414 | 0.414 | 0.414 | 29.89 | 70.11 |
|  | **50** | 0.48 | 0.48 | 0.48 | 0.48 | 18.75 | 81.25 |
|  | **100** | 0.532 | 0.532 | 0.532 | 0.532 | 9.73 | 90.27 |
| **6d** | **Conc.** | **Run1** | **Run2** | **Run3** | **Mean** | **% Viability** | **% Inhibition** |
|  | **0** | 1.034 | 1.034 | 1.034 | 1.034 | 100 | 0 |
|  | **6.25** | 0.18 | 0.18 | 0.18 | 0.18 | 82.56 | 17.44 |
|  | **12.5** | 0.343 | 0.343 | 0.343 | 0.343 | 66.86 | 33.14 |
|  | **25** | 0.58 | 0.58 | 0.58 | 0.58 | 43.87 | 56.13 |
|  | **50** | 0.82 | 0.82 | 0.82 | 0.82 | 20.62 | 79.38 |
|  | **100** | 0.917 | 0.917 | 0.917 | 0.917 | 11.31 | 88.69 |
| **6e** | **Conc.** | **Run1** | **Run2** | **Run3** | **Mean** | **% Viability** | **% Inhibition** |
|  | **0** | 0.903 | 0.903 | 0.903 | 0.903 | 100 | 0 |
|  | **6.25** | 0.717 | 0.717 | 0.717 | 0.717 | 79.36 | 20.64 |
|  | **12.5** | 0.55 | 0.55 | 0.55 | 0.55 | 60.88 | 39.12 |
|  | **25** | 0.409 | 0.409 | 0.409 | 0.409 | 45.63 | 54.37 |
|  | **50** | 0.18 | 0.18 | 0.18 | 0.18 | 19.88 | 80.12 |
|  | **100** | 0.09 | 0.09 | 0.09 | 0.09 | 10.01 | 93.99 |
| **10a** | **Conc.** | **Run1** | **Run2** | **Run3** | **Mean** | **% Viability** | **% Inhibition** |
|  | **0** | 0.436 | 0.436 | 0.436 | 0.436 | 100 | 0 |
|  | **6.25** | 0.342 | 0.342 | 0.342 | 0.342 | 78.42 | 21.58 |
|  | **12.5** | 0.25 | 0.25 | 0.25 | 0.25 | 56.85 | 43.15 |
|  | **25** | 0.19 | 0.19 | 0.19 | 0.19 | 43.52 | 56.48 |
|  | **50** | 0.087 | 0.087 | 0.087 | 0.087 | 19.91 | 81.09 |
|  | **100** | 0.043 | 0.043 | 0.043 | 0.043 | 9.87 | 92.13 |
| **10b** | **Conc.** | **Run1** | **Run2** | **Run3** | **Mean** | **% Viability** | **% Inhibition** |
|  | **0** | 0.508 | 0.508 | 0.508 | 0.508 | 100 | 0 |
|  | **6.25** | 0.409 | 0.409 | 0.409 | 0.409 | 80.6 | 19.64 |
|  | **12.5** | 0.299 | 0.299 | 0.299 | 0.299 | 58.78 | 41.22 |
|  | **25** | 0.22 | 0.22 | 0.22 | 0.22 | 42.62 | 57.38 |
|  | **50** | 0.085 | 0.085 | 0.085 | 0.085 | 16.78 | 83.22 |
|  | **100** | 0.044 | 0.044 | 0.044 | 0.044 | 8.55 | 91.45 |
| **10c** | **Conc.** | **Run1** | **Run2** | **Run3** | **mean** | **% Viability** | **% Inhibition** |
|  | **0** | 0.789 | 0.789 | 0.789 | 0.789 | 100 | 0 |
|  | **6.25** | 0.665 | 0.665 | 0.665 | 0.665 | 84.34 | 15.66 |
|  | **12.5** | 0.564 | 0.564 | 0.564 | 0.564 | 71.56 | 28.44 |
|  | **25** | 0.328 | 0.328 | 0.328 | 0.328 | 48.43 | 51.57 |
|  | **50** | 0.17 | 0.17 | 0.17 | 0.17 | 21.5 | 78.5 |
|  | **100** | 0.052 | 0.052 | 0.052 | 0.052 | 6.63 | 93.37 |
| **10d** | **Conc.** | **Run1** | **Run2** | **Run3** | **mean** | **% Viability** | **% Inhibition** |
|  | **0** | 1.304 | 1.301 | 1.301 | 1.304 | 100 | 0 |
|  | **6.25** | 0.76 | 0.78 | 0.74 | 0.76 | 58.31 | 41.69 |
|  | **12.5** | 0.56 | 0.56 | 0.56 | 0.56 | 41.67 | 58.33 |
|  | **25** | 0.272 | 0.269 | 0.275 | 0.272 | 20.86 | 79.14 |
|  | **50** | 0.099 | 0.099 | 0.099 | 0.099 | 7.6 | 92.4 |
|  | **100** | 0.062 | 0.062 | 0.061 | 0.062 | 4.7 | 95.3 |
| **14a** | **Conc.** | **Run1** | **Run2** | **Run3** | **mean** | **% Viability** | **% Inhibition** |
|  | **0** | 0.924 | 0.924 | 0.924 | 0.924 | 100 | 0 |
|  | **6.25** | 0.205 | 0.205 | 0.205 | 0.205 | 85.44 | 14.56 |
|  | **12.5** | 0.555 | 0.555 | 0.555 | 0.555 | 60.12 | 39.88 |
|  | **25** | 0.244 | 0.244 | 0.244 | 0.244 | 26.44 | 73.56 |
|  | **50** | 0.082 | 0.082 | 0.082 | 0.082 | 8.89 | 91.11 |
|  | **100** | 0.033 | 0.033 | 0.033 | 0.033 | 3.55 | 96.45 |
| **14b** | **Conc.** | **Run1** | **Run2** | **Run3** | **mean** | **% Viability** | **% Inhibition** |
|  | **0** | 0.85 | 1.007 | 1.2 | 1.019 | 100 | 0 |
|  | **6.25** | 0.845 | 0.845 | 0.845 | 0.845 | 82.98 | 17.02 |
|  | **12.5** | 0.536 | 0.521 | 0.524 | 0.527 | 51.75 | 48.25 |
|  | **25** | 0.355 | 0.355 | 0.346 | 0.352 | 34.57 | 65.43 |
|  | **50** | 0.25 | 0.25 | 0.25 | 0.25 | 24.52 | 75.48 |
|  | **100** | 0.032 | 0.036 | 0.034 | 0.034 | 3.36 | 93.64 |
| **15** | **Conc.** | **Run1** | **Run2** | **Run3** | **mean** | **% Viability** | **% Inhibition** |
|  | **0** | 1.031 | 1.031 | 1.031 | 1.031 | 100 | 0 |
|  | **6.25** | 0.782 | 0.782 | 0.782 | 0.782 | 75.92 | 24.08 |
|  | **12.5** | 0.683 | 0.684 | 0.682 | 0.683 | 66.39 | 33.61 |
|  | **25** | 0.302 | 0.302 | 0.302 | 0.302 | 29.31 | 70.69 |
|  | **50** | 0.108 | 0.108 | 0.108 | 0.108 | 10.57 | 89.43 |
|  | **100** | 0.046 | 0.046 | 0.046 | 0.046 | 4.52 | 95.48 |
| **16** | **Conc.** | **Run1** | **Run2** | **Run3** | **mean** | **% Viability** | **% Inhibition** |
|  | **0** | 1.61 | 1.63 | 1.59 | 1.61 | 100 | 0 |
|  | **6.25** | 0.93 | 0.93 | 0.96 | 0.93 | 61.99 | 38.01 |
|  | **12.5** | 0.742 | 0.742 | 0.742 | 0.742 | 48.4 | 51.6 |
|  | **25** | 0.505 | 0.505 | 0.505 | 0.505 | 20.5 | 79.5 |
|  | **50** | 0.172 | 0.163 | 0.181 | 0.172 | 7.77 | 92.23 |
|  | **100** | 0.089 | 0.089 | 0.089 | 0.089 | 4.31 | 95.69 |
| **Blank ref** | **Conc.** | **Run1** | **Run2** | **Run3** | **mean** | **% Viability** | **% Inhibition** |
|  | **0** | 0.811 | 0.811 | 0.811 | 0.811 | 100 | 0 |
|  | **6.25** | 0.558 | 0.558 | 0.558 | 0.558 | 68.75 | 31.25 |
|  | **12.5** | 0.234 | 0.234 | 0.234 | 0.234 | 28.91 | 71.09 |
|  | **25** | 0.134 | 0.134 | 0.134 | 0.134 | 16.58 | 83.42 |
|  | **50** | 0.03 | 0.03 | 0.03 | 0.03 | 3.67 | 96.33 |
|  | **100** | 0.008 | 0.008 | 0.008 | 0.008 | 0.95 | 99.05 |
| **Blank ref Cis** | **Conc.** | **Run1** | **Run2** | **Run3** | **mean** | **% Viability** | **% Inhibition** |
|  | **0** | 0.896 | 0.81 | 0.967 | 0.891 | 100 | 0 |
|  | **6.25** | 0.664 | 0.803 | 0.684 | 0.717 | 80.52 | 19.48 |
|  | **12.5** | 0.404 | 0.408 | 0.406 | 0.406 | 45.67 | 54.33 |
|  | **25** | 0.162 | 0.179 | 0.169 | 0.17 | 18.96 | 81.04 |
|  | **50** | 0.067 | 0.067 | 0.067 | 0.067 | 7.51 | 92.49 |
|  | **100** | 0.033 | 0.033 | 0.033 | 0.033 | 3.66 | 96.34 |


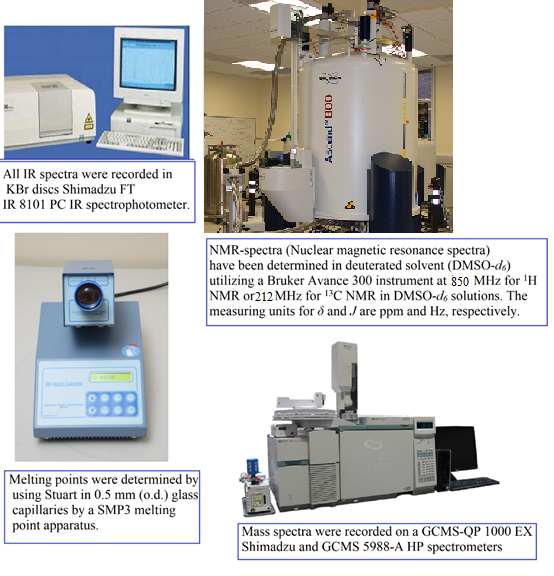

Supplement: S1 File — (DOCX) [file pone.0274459.s001.docx]
